# Supplementary material for: Landscape of Conditional eQTL in Dorsolateral Prefrontal Cortex and Co-localization with Schizophrenia GWAS
Source: Am J Hum Genet. 2018 May 24;102(6):1169–84. doi: 10.1016/j.ajhg.2018.04.011 (PMC5993513; doi:10.1016/j.ajhg.2018.04.011)
Supplement: Document S1. Figures S1–S18 [file mmc1.pdf]

The American Journal of Human Genetics, Volume *102*

Supplemental Data

Landscape of Conditional eQTL

in Dorsolateral Prefrontal Cortex

and Co-localization with Schizophrenia GWAS

Amanda Dobbyn, Laura M. Huckins, James Boocock, Laura G. Sloofman, Benjamin S. Glicksberg, Claudia Giambartolomei, Gabriel E. Hoffman, Thanneer M. Perumal, Kiran Girdhar, Yan Jiang, Towfique Raj, Douglas M. Ruderfer, Robin S. Kramer, Dalila Pinto, the CommonMind Consortium, Schahram Akbarian, Panos Roussos, Enrico Domenici, Bernie Devlin, Pamela Sklar, Eli A. Stahl, and Solveig K. Sieberts

SUPPLEMENTAL FIGURES

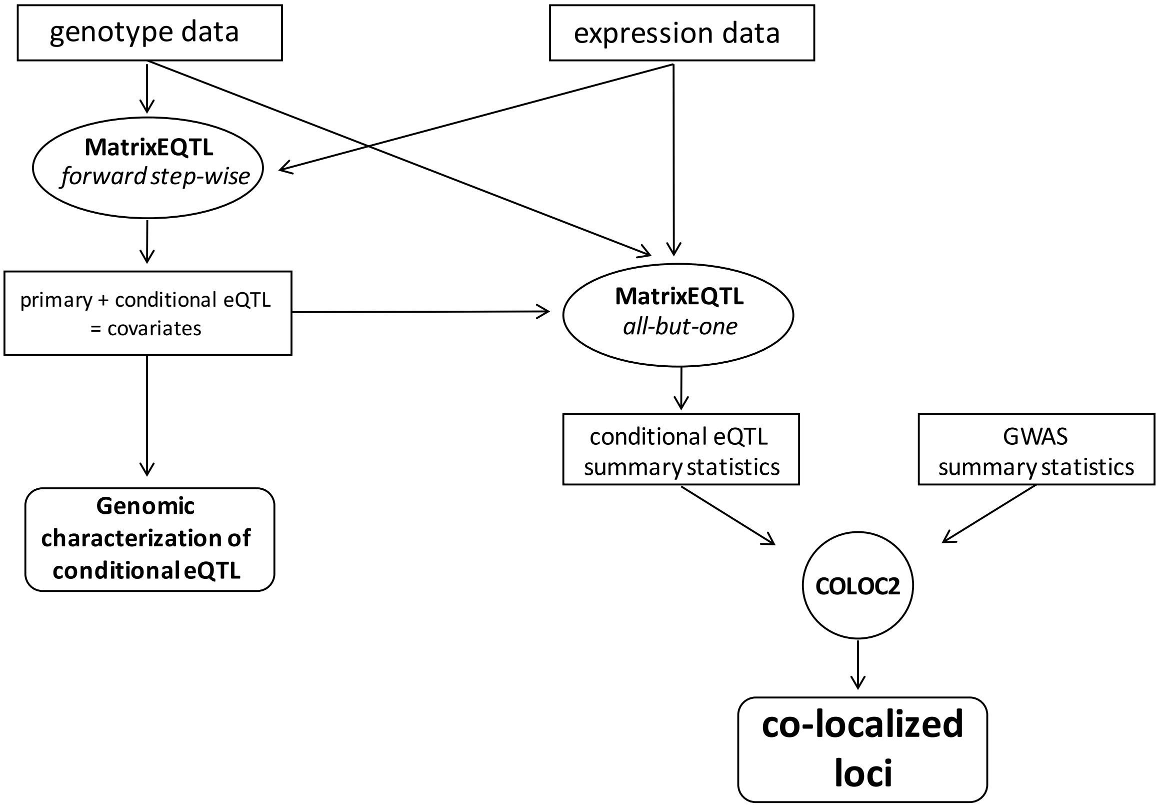

Figure S1. Overview of Workflow

Genotype and DLPFC expression data from the CommonMind Consortium are used to identify primary and conditional eQTL, implemented in MatrixEQTL. The resulting conditionally independent eQTL are then used as covariates, along with the genotype and expression data, to isolate each independent eQTL signature in an “all-but-one” analysis. These independent eQTL summary statistics and the 2014 PGC schizophrenia GWAS summary statistics are used as input to COLOC2, in order to test for co-localization between eQTL and GWAS signatures.

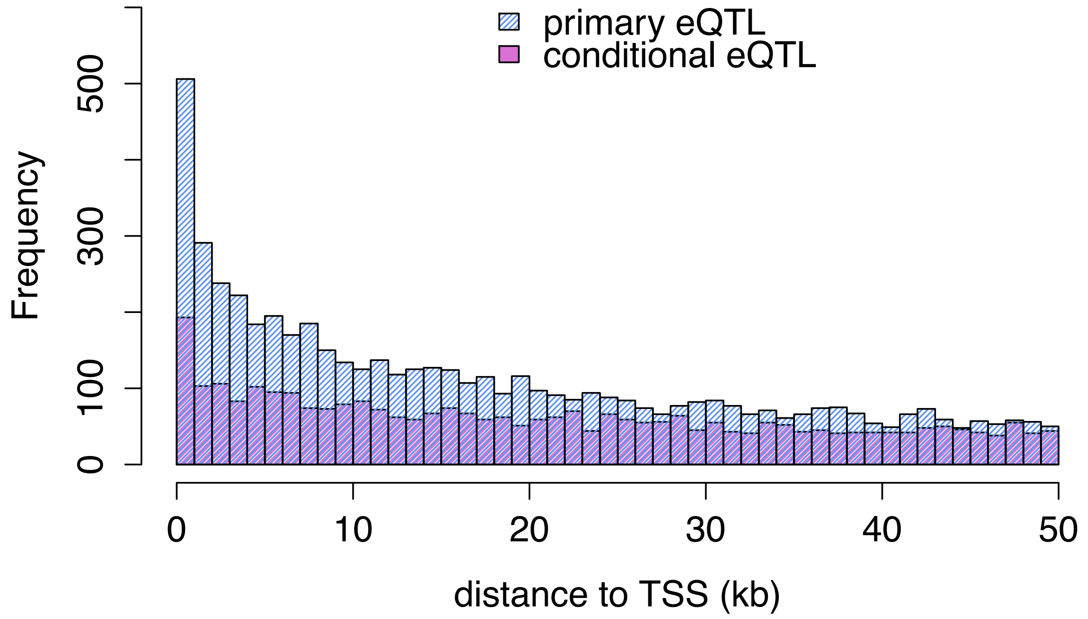

Figure S2. Distance from eQTL to transcription start site (TSS)

An overlapping histogram showing the numbers of eQTL (y-axis) occurring at increasing distances to TSS (x-axis), focusing on the 50 Kb window around the TSS. Even when examining smaller distances to the TSS, there is a difference between primary (blue) and conditional (pink) eQTL distance to TSS, with primary eQTL generally found closer to the TSS than conditional eQTL.

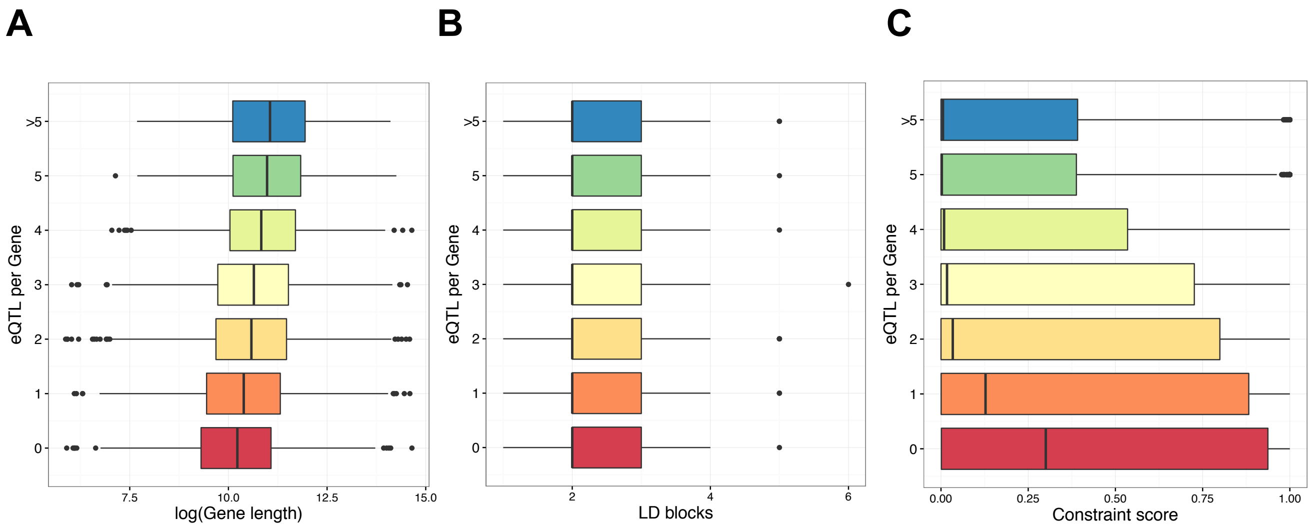

Figure S3. Correlation Between Number of Independent eQTL per Gene and Gene Length, Number of LD Blocks in the Cis- Region, and Genic Constraint

Panels (A-C) show the correlation between the number of independent eQTL per gene (0, 1, 2, 3, 4, 5, ≥6 eQTL) on the y-axis and (A) log(gene length) calculated using Ensembl gene locations, (B) number of LD blocks overlapping the cis-region (gene +/- 1Mb), and (C) genic constraint score (ExAC, based on LoF variants present) on the x-axes. There was a positive correlation between number of eQTL per gene and both log(gene length) and number of LD blocks, and there was a negative correlation between number of eQTL per gene and genic constraint score.

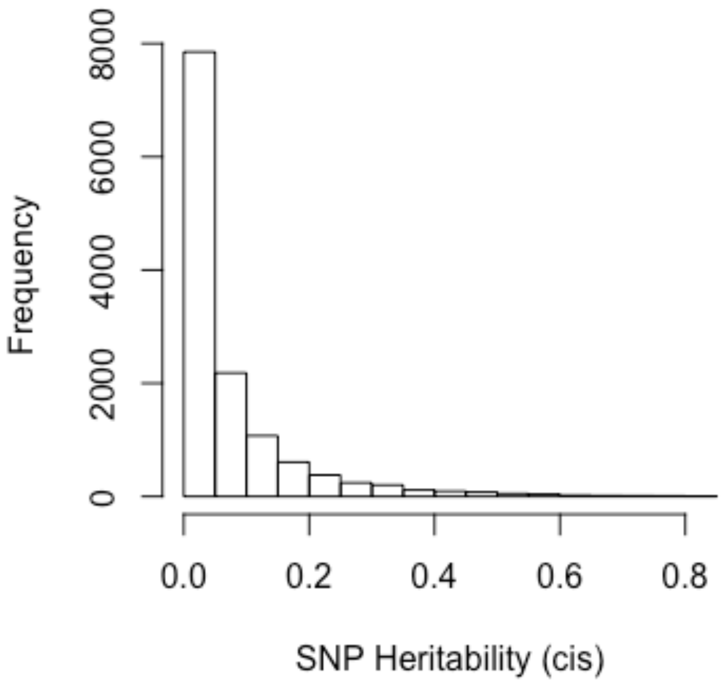

Figure S4. Distribution of Cis-SNP-Heritability per Gene

Counts of the numbers of genes (y-axis) whose variance in expression (ranging from 0 to 1, x-axis) is explained by the SNPs in its cis- region. Cis- heritability was estimated from the dosages of all imputed SNPs in the gene's cis- region (+/- 1Mb), after filtering out SNPs with MAF < 0.05 and INFO score < 0.90, using GCTA. Dosages were taken from the 467 European-ancestry individuals in the CMC cohort.

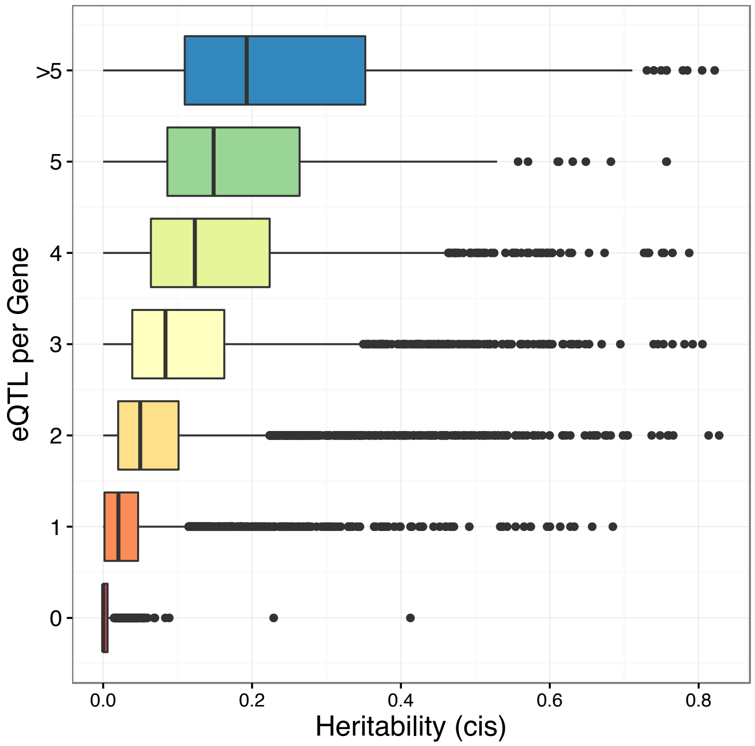

Figure S5. Correlation Between Number of Independent eQTL per Gene and Cis-Heritability

There is a positive correlation between the number of independent eQTL per gene (0, 1, 2, 3, 4, 5, ≥6 eQTL) on the y-axis and variance of expression explained by SNPs in the cis- region (cis- heritability) on the x axis.

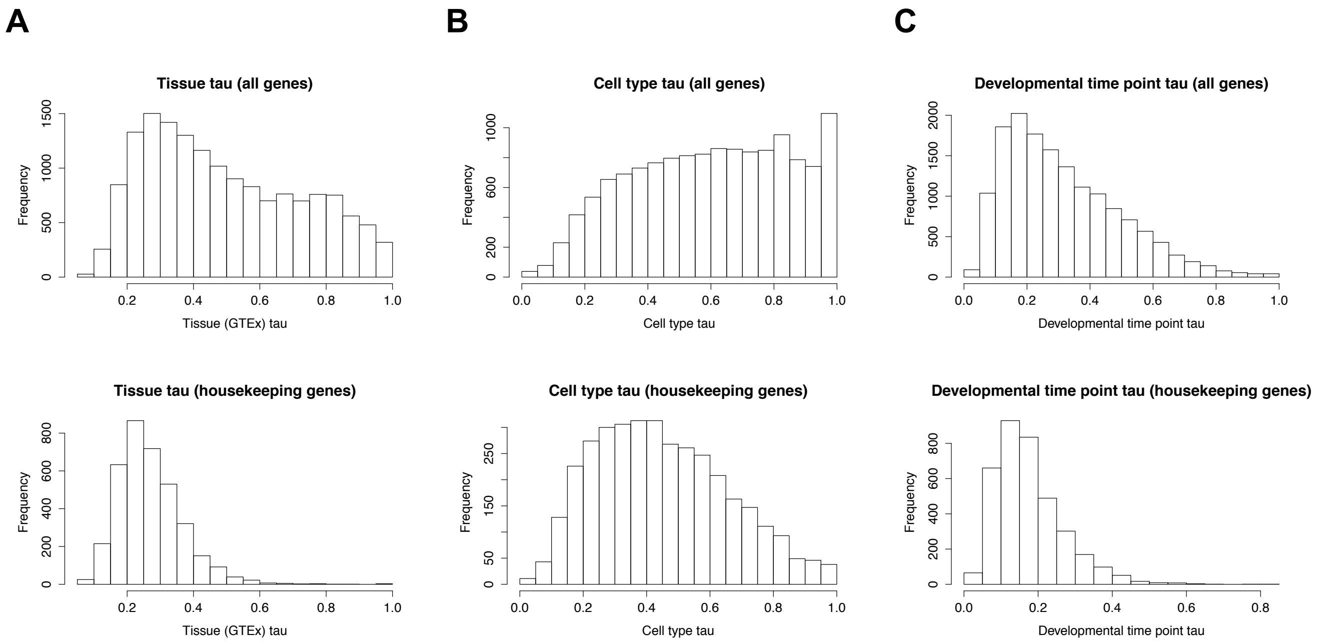

Figure S6. Distributions for Tissue, Cell Type, and Developmental Time Point Tau

Panels (A-C) show the counts of genes (y-axis) with a given tissue (A), cell type (B), or developmental time point (C) Tau (ranging from 0 to 1, x-axis), for all autosomal genes tested (top), and for a subset of housekeeping genes (bottom). Tissue Tau was calculated using expression data for 53 tissues from the GTEx project, cell type Tau was calculated using single-cell expression data for six DLPFC cell types, and developmental time point Tau was calculated using expression data for eight developmental time points from the BrainSpan atlas. For all tau measures, 0 indicates low specificity of expression, and 1 indicates high specificity of expression. The Tau distributions for housekeeping genes are all skewed right in comparison to the distributions for all genes, reflecting lower context specificity of expression for housekeeping genes.

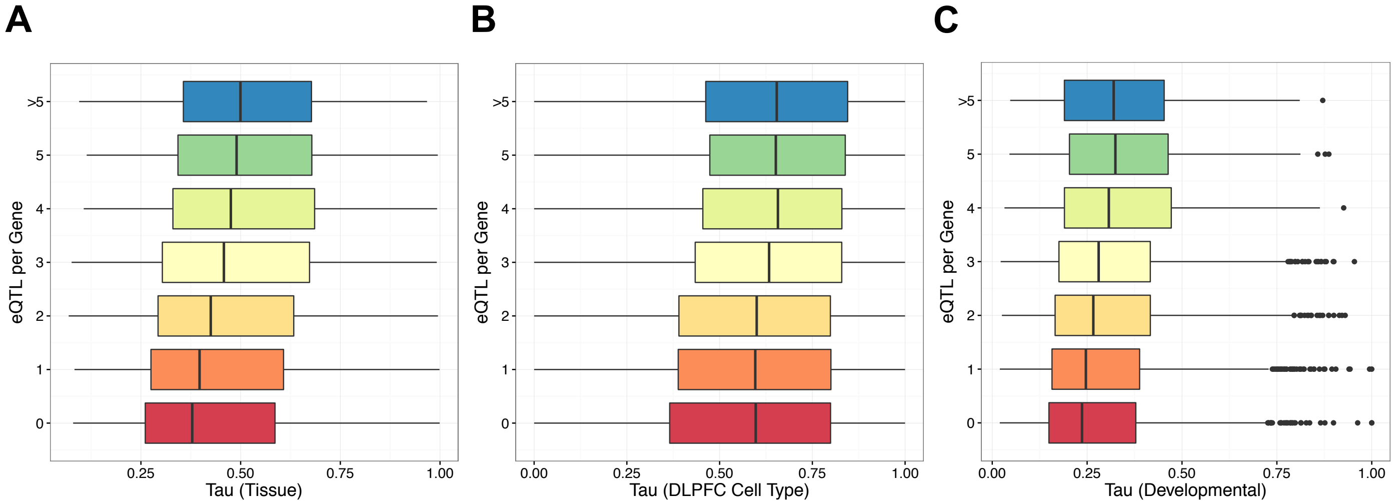

Figure S7. Correlation Between Number of Independent eQTL per Gene and Tissue, Cell Type, and Developmental Time Point Tau

Panels (A-C) show the correlation between the number of independent eQTL per gene (0, 1, 2, 3, 4, 5, ≥6 eQTL) on the y-axis and (A) tissue Tau, (B) cell type Tau, and (C) developmental time point Tau on the x-axes. There are positive correlations between the number of eQTL per gene and all three Tau measures. The strongest correlation observed is for cell type Tau.

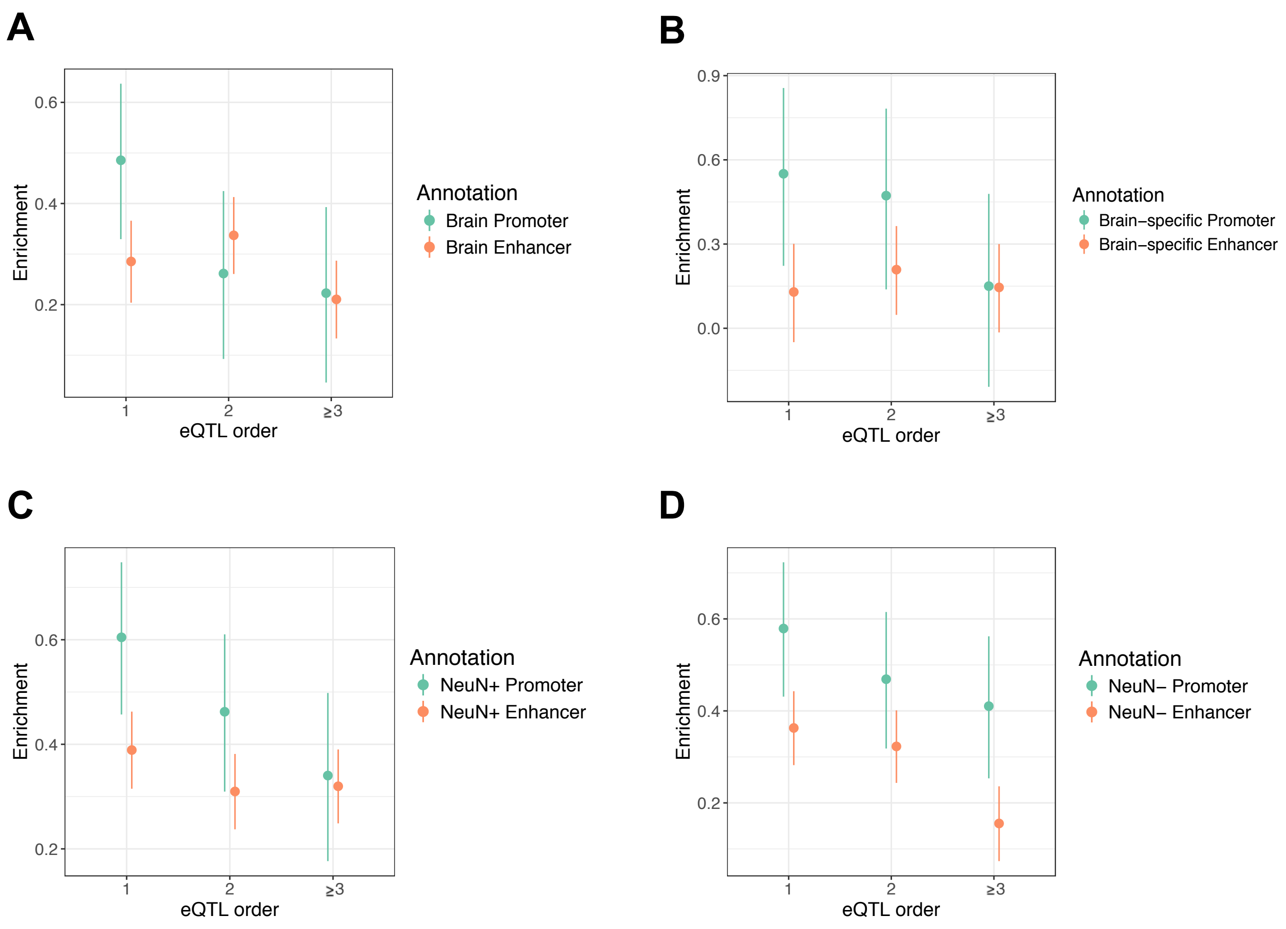

Figure S8. Enrichments of Primary and Conditional eQTL in Active Regulatory Annotations, Restricting to Match SNPs Near Brain-Expressed Genes

Plotted are enrichments (regression coefficient estimate ± 95% CI from logistic regression, y-axes) of primary (x-axis eQTL order = 1) and conditional (eQTL order = 2, ≥3) eQTL in functional annotations. Control SNPs were selected on the basis of being within 1 Mb of any of the 16,423 genes that were expressed in DLPFC and tested for eQTL. Panels (A) and (B) show enrichment in brain (union of all individual brain regions) and brain-specific (present in brain but not in seven other non-brain tissues) active promoter (turquoise) and enhancer (orange) ChromHMM states from the NIH Roadmap Epigenomics Project. Panel (C) shows enrichment in neuronal nuclei (NeuN+), for active promoters (intersection of DLPFC H3K4me3 and H3K27ac ChIP-seq peaks, turquoise) and enhancers (H3K27 peaks that do not overlap H3K4me3 peaks, orange). Panel (D) shows enrichments in the same annotations, but for DLPFC non-neuronal nuclei (NeuN-).

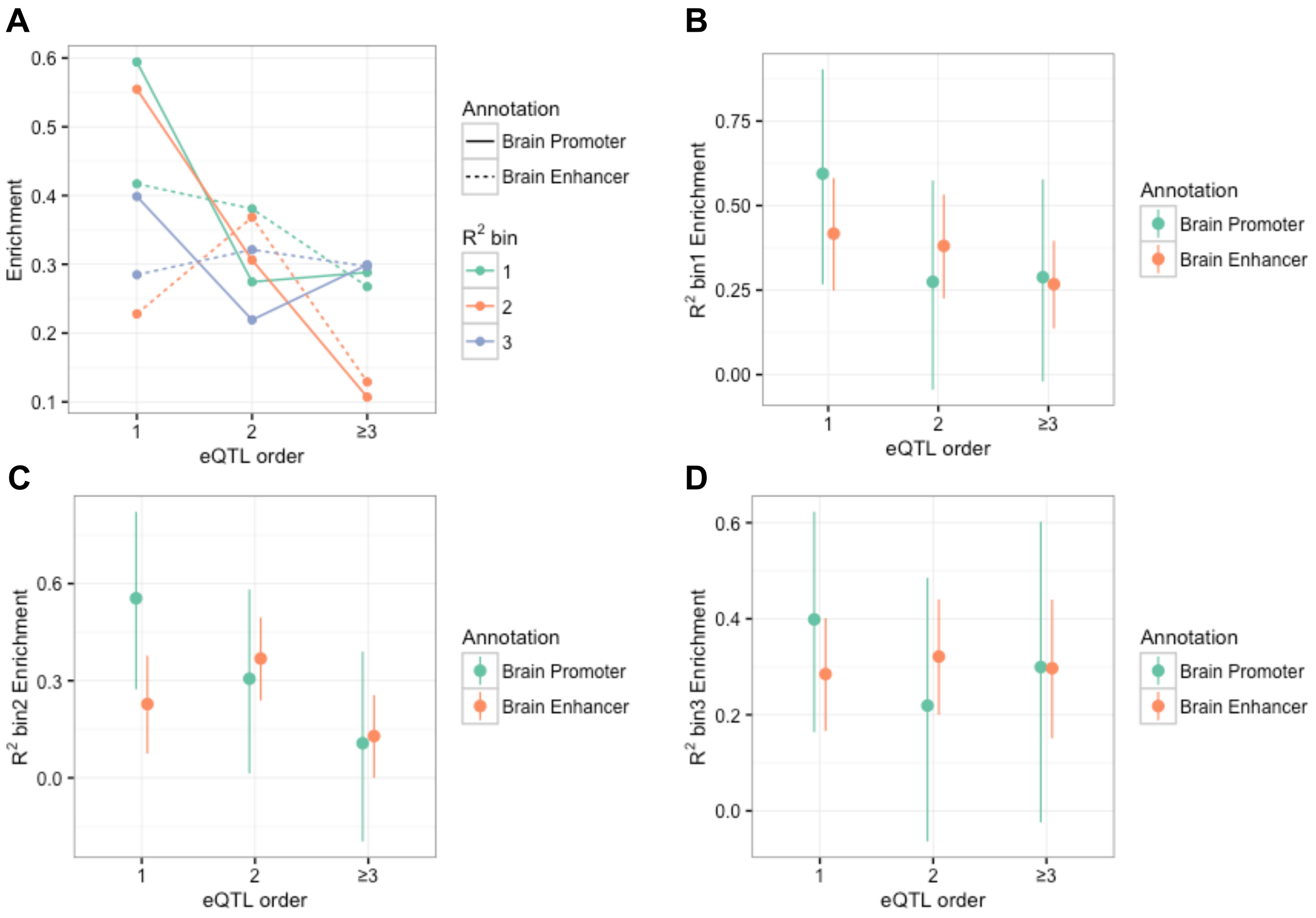

Figure S9. Variance-stratified Enrichments of Primary and Conditional eQTL in Brain Promoters and Enhancers

Plotted are enrichments (regression coefficient estimate ± 95% CI from logistic regression, y-axes) of primary (x-axis eQTL order = 1) and conditional (eQTL order = 2, ≥3) eQTL, stratified by variance in expression explained (R^2) by each eQTL, in brain promoters and enhancers. Primary and conditional eQTL were binned by R^2 value, where bin 1 consists of eQTL with the smallest R^2 estimates, and bin 3 consists of those with the highest. Panel (A) shows enrichments for all R^2 bins. Panels (B-D) show enrichments for R^2 bins 1, 2, and 3, respectively.

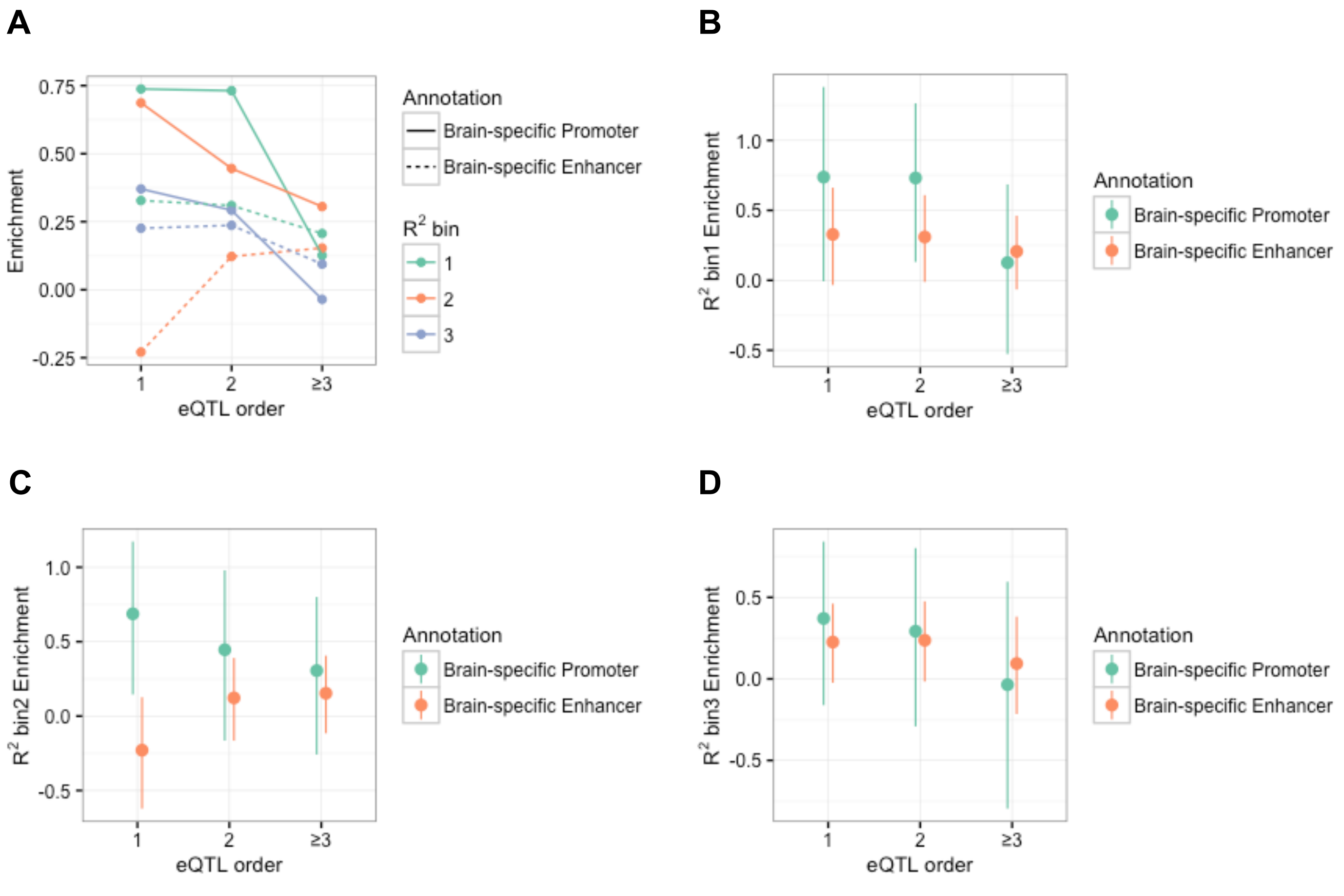

Figure S10. Variance-stratified Enrichments of Primary and Conditional eQTL in Brain-specific Promoters and Enhancers

Plotted are enrichments (regression coefficient estimate ± 95% CI from logistic regression, y-axes) of primary (x-axis eQTL order = 1) and conditional (eQTL order = 2, ≥3) eQTL, stratified by variance in expression explained (R^2) by each eQTL, in brain-specific promoters and enhancers. Primary and conditional eQTL were binned by R^2 value, where bin 1 consists of eQTL with the smallest R^2 estimates, and bin 3 consists of those with the highest. Panel (A) shows enrichments for all R^2 bins. Panels (B-D) show enrichments for R^2 bins 1, 2, and 3, respectively.

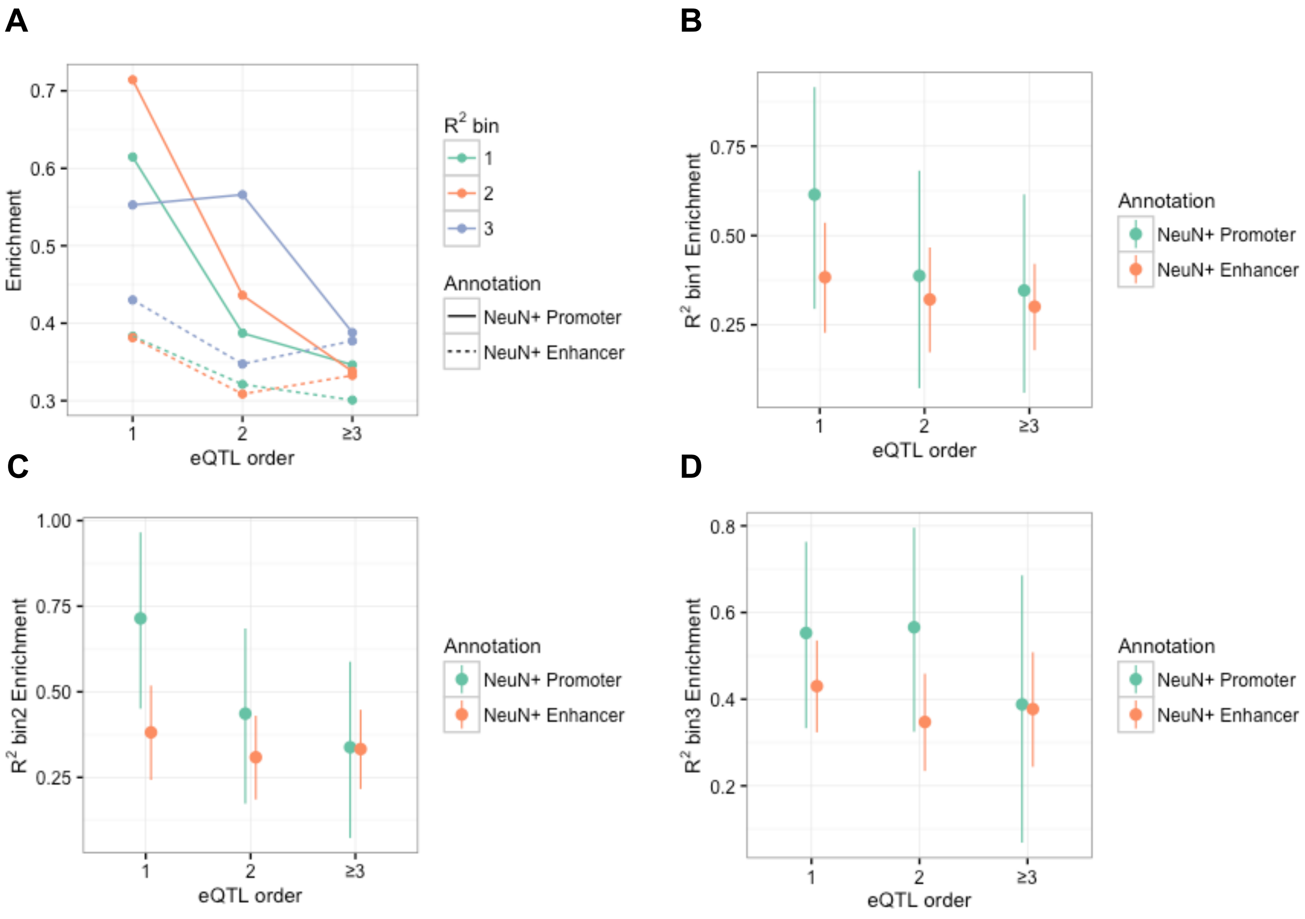

Figure S11. Variance-stratified Enrichments of Primary and Conditional eQTL in DLPFC Neuronal Promoters and Enhancers

Plotted are enrichments (regression coefficient estimate ± 95% CI from logistic regression, y-axes) of primary (x-axis eQTL order = 1) and conditional (eQTL order = 2, ≥3) eQTL, stratified by variance in expression explained (R^2) by each eQTL, in DLPFC neuronal (NeuN+) promoters and enhancers. Primary and conditional eQTL were binned by R^2 value, where bin 1 consists of eQTL with the smallest R^2 estimates, and bin 3 consists of those with the highest. Panel (A) shows enrichments for all R^2 bins. Panels (B-D) show enrichments for R^2 bins 1, 2, and 3, respectively.

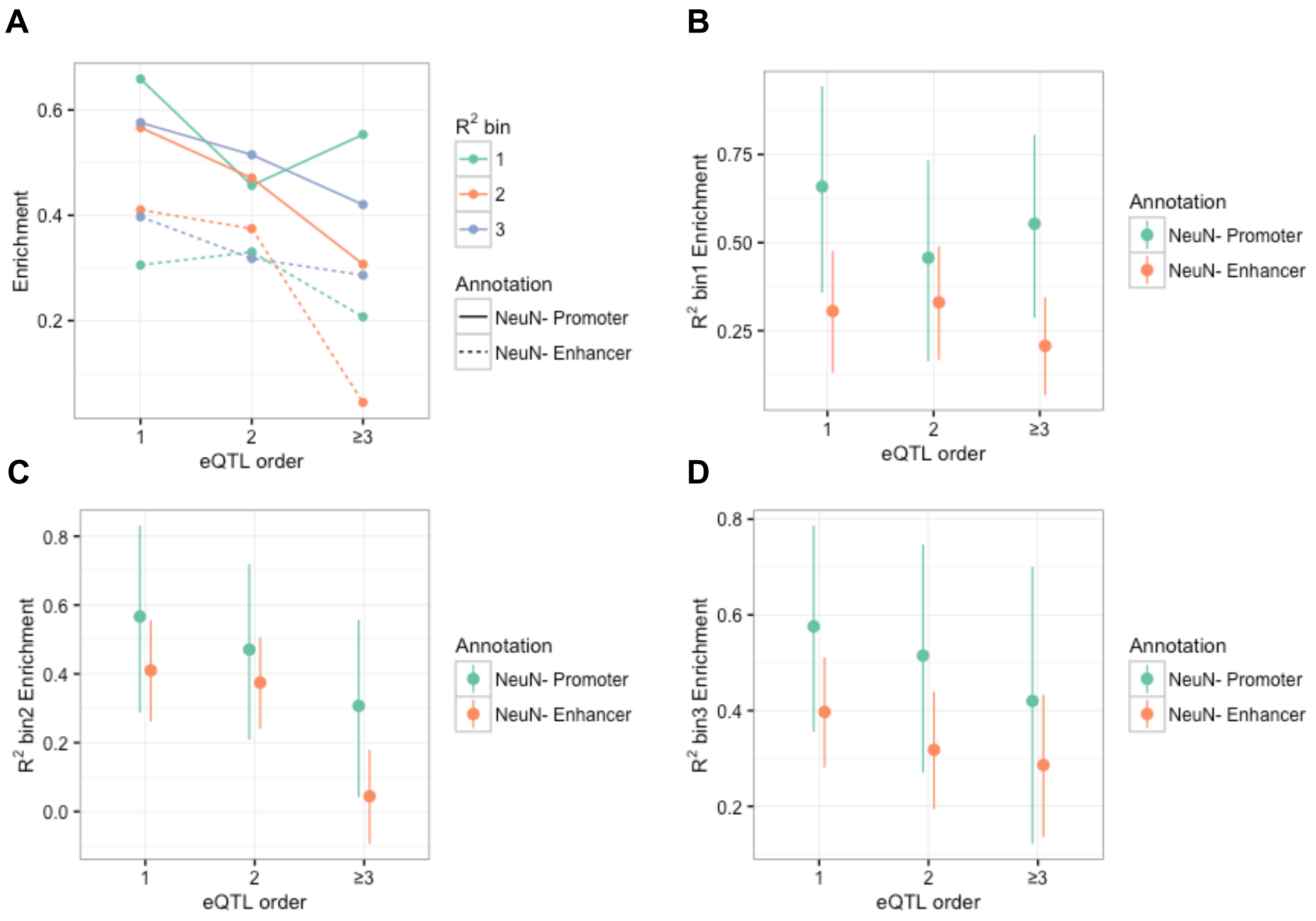

Figure S12. Variance-stratified Enrichments of Primary and Conditional eQTL in DLPFC Non-neuronal Promoters and Enhancers

Plotted are enrichments (regression coefficient estimate ± 95% CI from logistic regression, y-axes) of primary (x-axis eQTL order = 1) and conditional (eQTL order = 2, ≥3) eQTL, stratified by variance in expression explained (R^2) by each eQTL, in DLPFC non-neuronal (NeuN-) promoters and enhancers. Primary and conditional eQTL were binned by R^2 value, where bin 1 consists of eQTL with the smallest R^2 estimates, and bin 3 consists of those with the highest. Panel (A) shows enrichments for all R^2 bins. Panels (B-D) show enrichments for R^2 bins 1, 2, and 3, respectively.

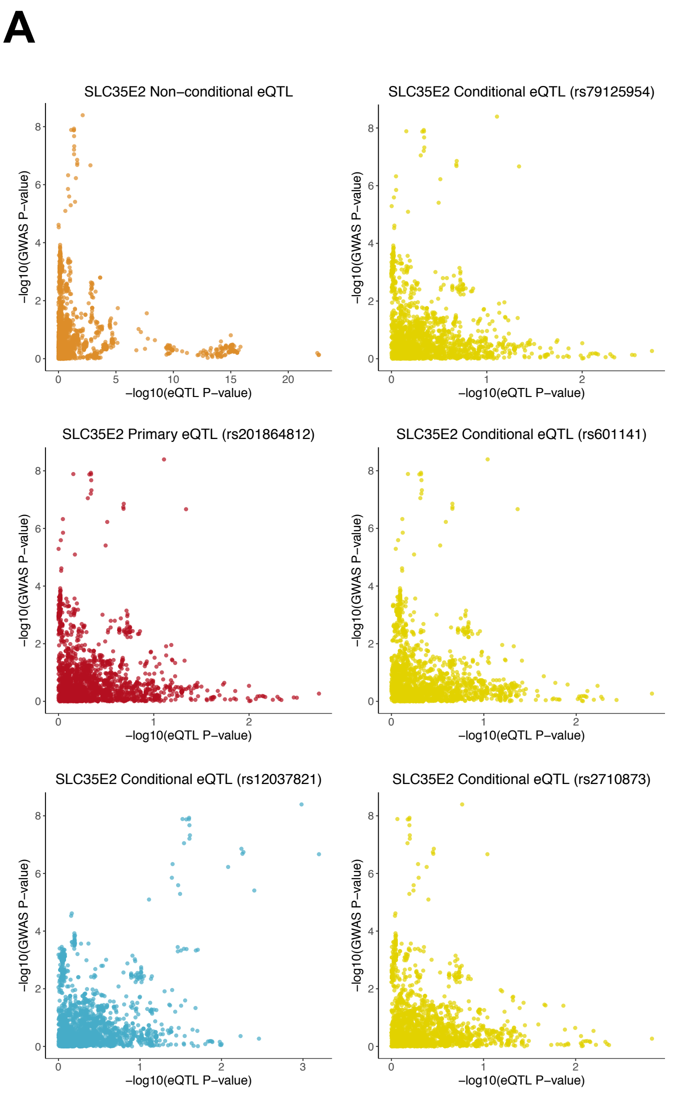

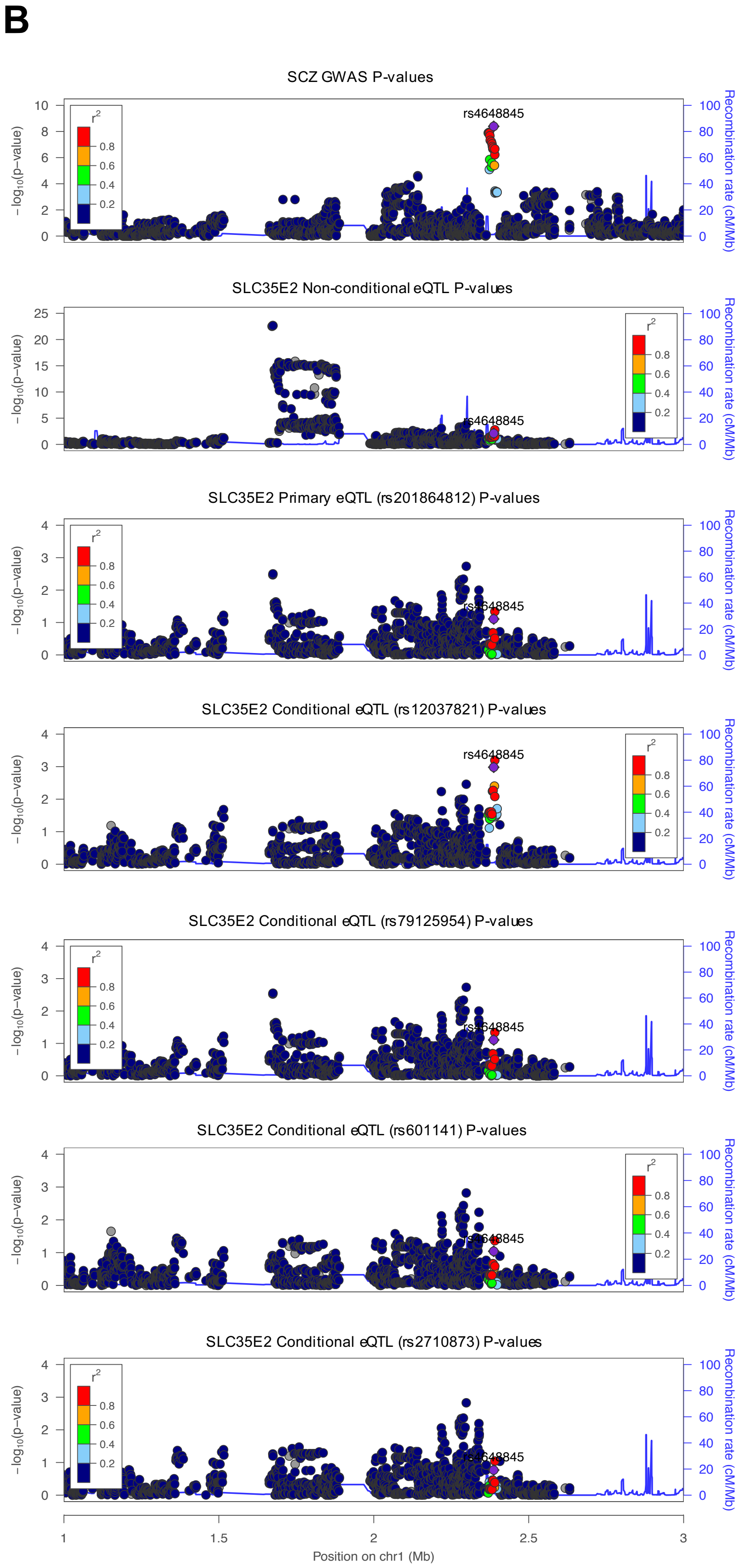

Figure S13. GWAS Signature for *SLC35E2* Co-localizes with the rs12037821 Conditional eQTL Signature

P-P plots (A) comparing GWAS P-values (y-axes) with eQTL P-values resulting from the “all-but-one” conditional analysis (x-axes) show the highest correlation to be that between the GWAS P-values and the rs12037821 conditional eQTL P-values (bottom left, blue). LocusZoom plots for the *SLC35E2* locus (B) show the GWAS signal (top) to more closely resemble the rs12037821 conditional eQTL signal (fourth from top) than the primary eQTL signal (rs201864812, third from top), non-conditional eQTL signal (second from top), or additional conditional eQTL signals. For all LocusZoom plots LD is colored with respect to the GWAS lead SNP.

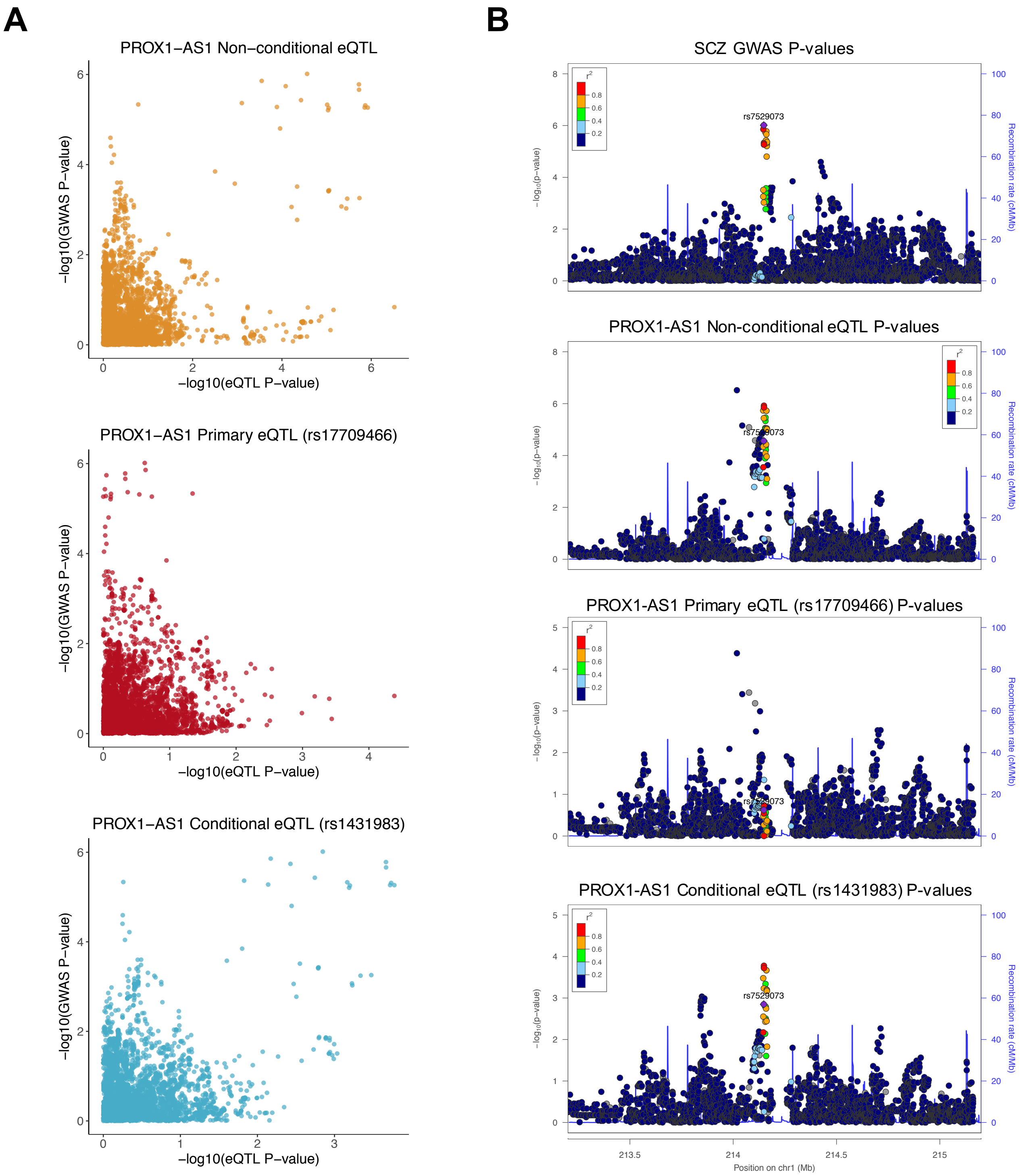

Figure S14. GWAS Signature for *PROX1-AS1* Co-localizes with the Conditional eQTL Signature

P-P plots (A) comparing GWAS P-values (y-axes) with eQTL P-values resulting from the “all-but-one” conditional analysis (x-axes) show the highest correlation to be that between the GWAS P-values and the rs1431983 conditional eQTL P-values (blue, bottom). LocusZoom plots for the *PROX1-AS1* locus (B) show the GWAS signal (top) to more closely resemble the conditional eQTL signal (rs1431983, bottom) than the primary eQTL signal (rs17709466, third from top) or non-conditional eQTL signal (second from top). For all LocusZoom plots LD is colored with respect to the GWAS lead SNP.

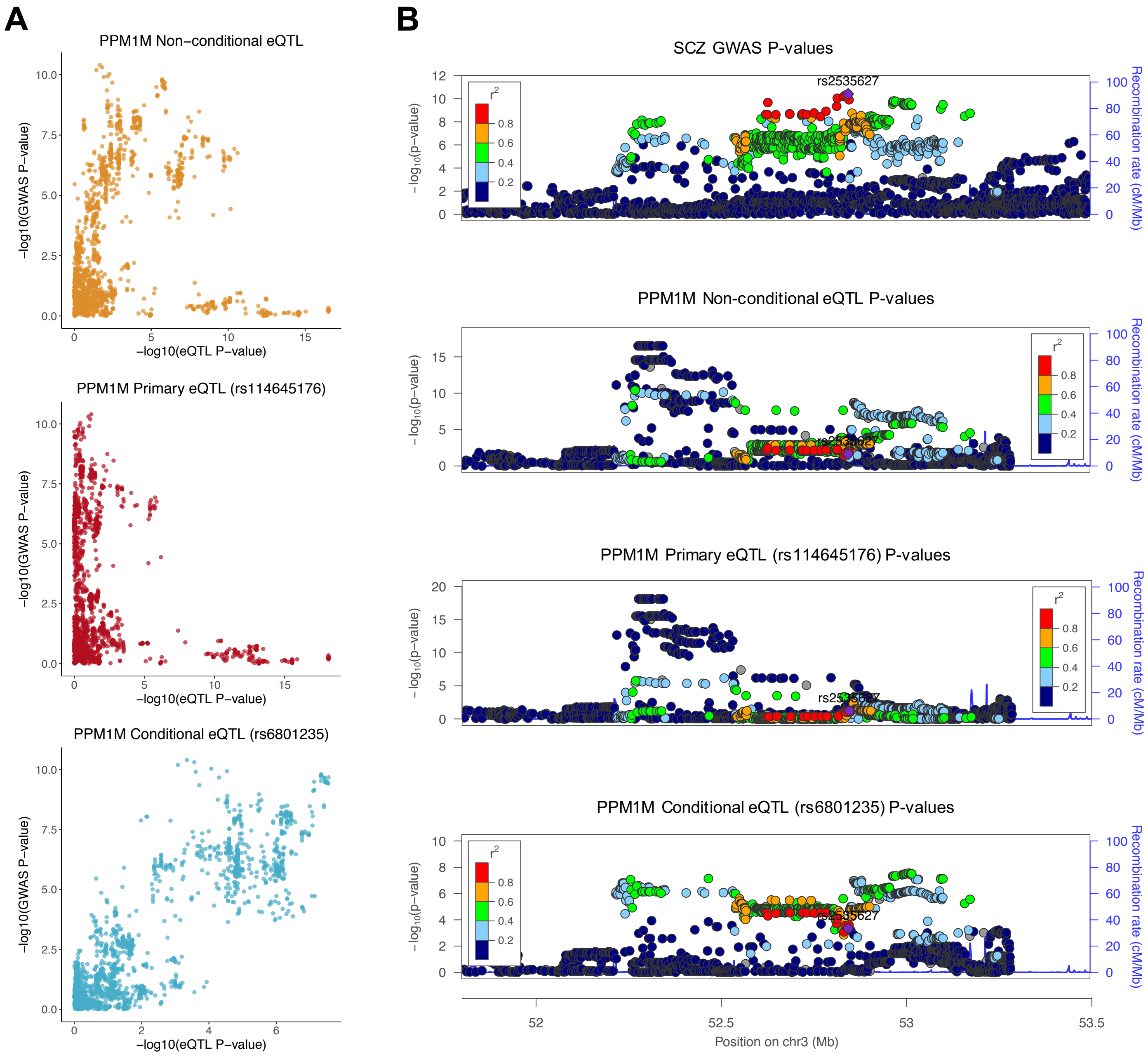

Figure S15. GWAS Signature for *PPM1M* Co-localizes with the Conditional eQTL Signature

P-P plots (A) comparing GWAS P-values (y-axes) with eQTL P-values resulting from the “all-but-one” conditional analysis (x-axes) show the highest correlation to be that between the GWAS P-values and the rs6801235 conditional eQTL P-values (blue, bottom). LocusZoom plots for the *PPM1M* locus (B) show the GWAS signal (top) to more closely resemble the conditional eQTL signal (rs6801235, bottom) than the primary eQTL signal (rs114645176, third from top) or non-conditional eQTL signal (second from top). For all LocusZoom plots LD is colored with respect to the GWAS lead SNP.

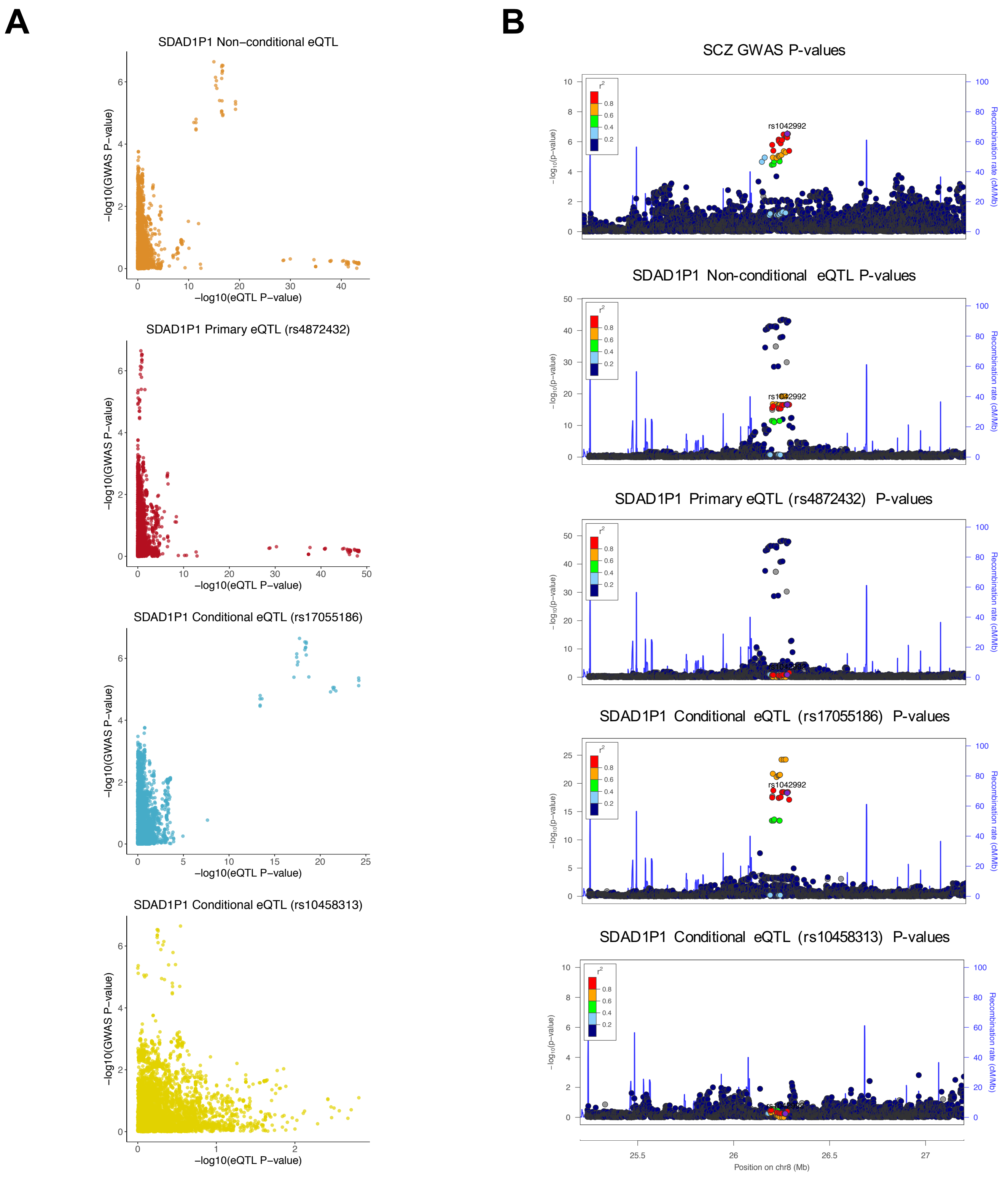

Figure S16. GWAS Signature for *SDAD1P1* Co-localizes with the Conditional eQTL Signature

P-P plots (A) comparing GWAS P-values (y-axes) with eQTL P-values resulting from the “all-but-one” conditional analysis (x-axes) shows the highest correlation to be that between the GWAS P-values and the rs17055186 conditional eQTL P-values (blue, third from top). LocusZoom plots for the *SDAD1P1* locus (B) show the GWAS signal (top) to more closely resemble the rs17055186 conditional eQTL signal (fourth from top) than the primary eQTL signal (rs4872432, third from top), non-conditional eQTL signal (second from top), or rs10458313 conditional eQTL signal. For all LocusZoom plots LD is colored with respect to the GWAS lead SNP.

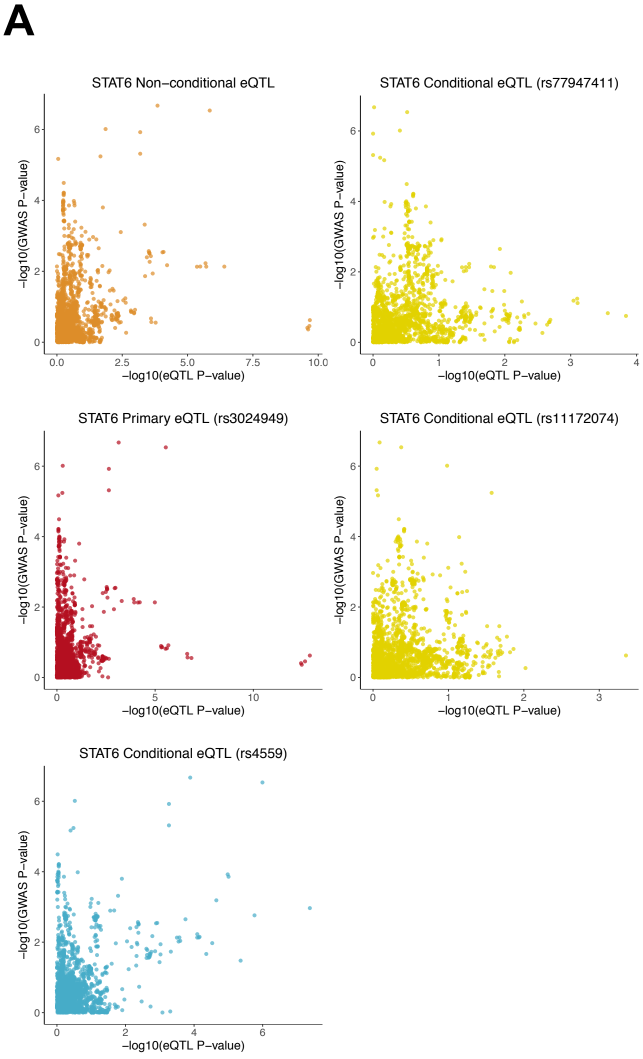

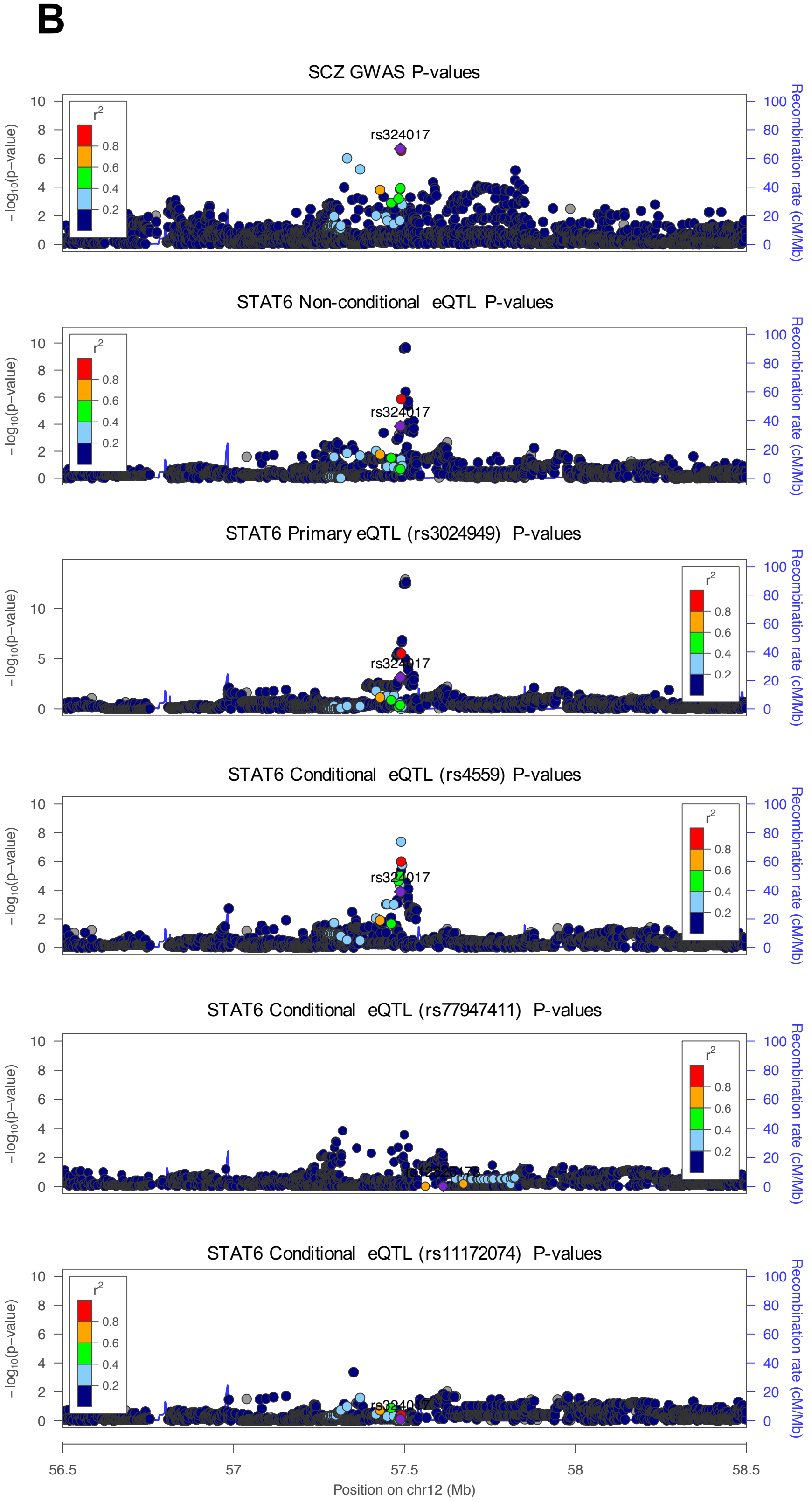

Figure S17. GWAS Signature for *STAT6* Co-localizes with the rs4559 Conditional eQTL Signature

P-P plots (A) comparing GWAS P-values (y-axes) with eQTL P-values resulting from the “all-but-one” conditional analysis (x-axes) show the highest correlation to be that between the GWAS P-values and the rs4559 conditional eQTL P-values blue, bottom left). LocusZoom plots for the *STAT6* locus (B) show the GWAS signal (top) to more closely resemble the rs4559 conditional eQTL signal (fourth from top) than the primary eQTL signal (rs3024949, third from top), non-conditional eQTL signal (second from top), or other conditional eQTL signals. For all LocusZoom plots LD is colored with respect to the GWAS lead SNP.

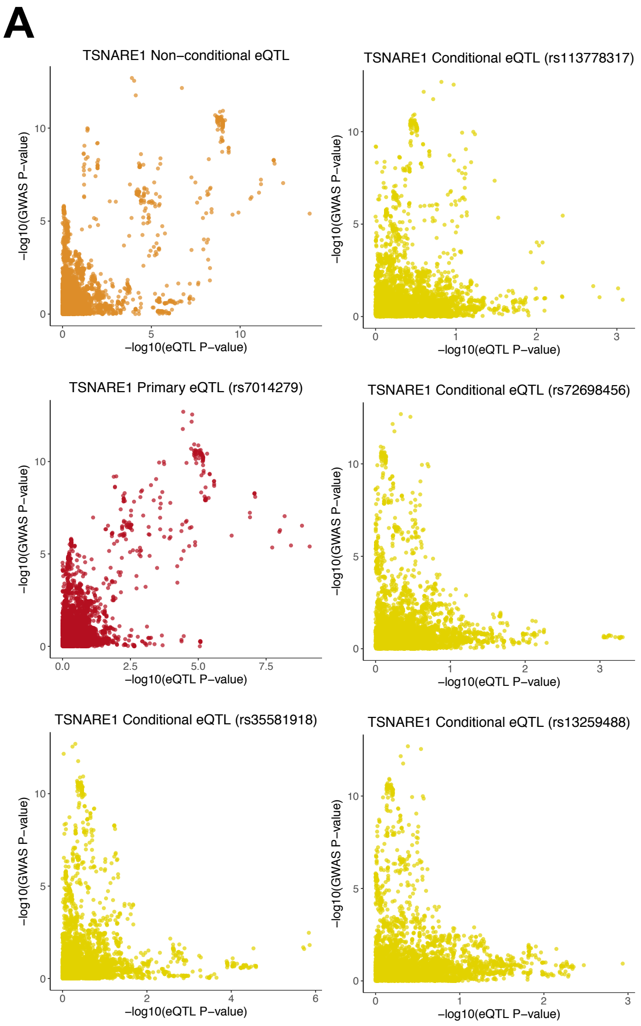

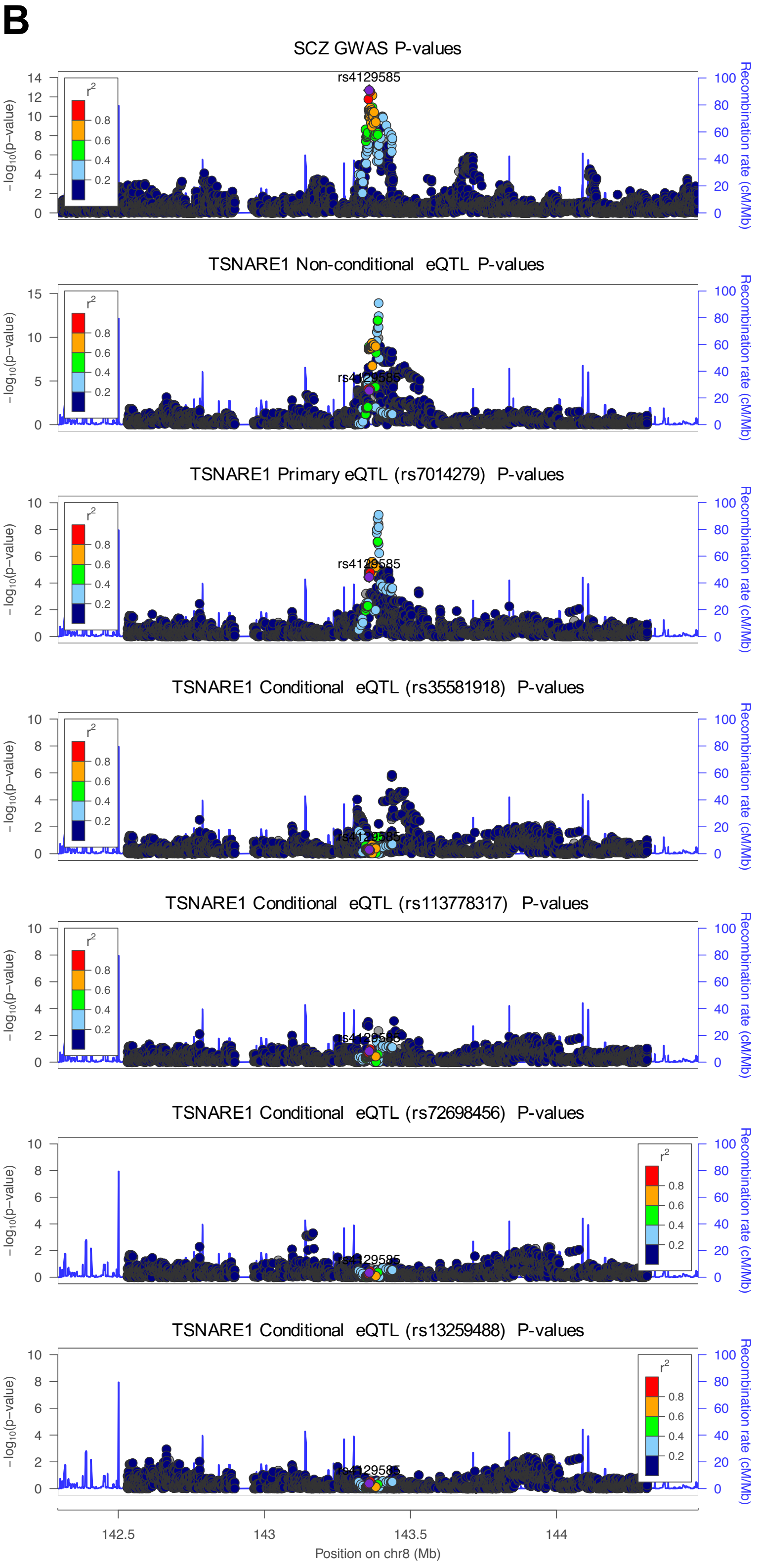

Figure S18. Lack of Co-localization Between *TSNARE1* eQTL and GWAS Signatures

P-P plots (A) comparing GWAS P-values (y-axes) with eQTL P-values resulting from the "all-but-one" conditional analysis (x-axes) suggest co-localization between the GWAS and primary eQTL (rs7014279, red) signals, but LocusZoom plots for the *TSNARE1* locus (B) show neither the non-conditional eQTL signal (second from top) nor the primary eQTL signal (third from top) to co-localize with the GWAS signal (top). For all LocusZoom plots LD is colored with respect to the GWAS lead SNP.
