# Supplementary material for: Landscape of Conditional eQTL in Dorsolateral Prefrontal Cortex and Co-localization with Schizophrenia GWAS
Source: Am J Hum Genet. 2018 May 24;102(6):1169–84. doi: 10.1016/j.ajhg.2018.04.011 (PMC5993513; doi:10.1016/j.ajhg.2018.04.011)
Supplement: Document S2. Article plus Supplemental Data [file mmc3.pdf]

Landscape of Conditional eQTL in Dorsolateral Prefrontal Cortex and Co-localization with Schizophrenia GWAS

Amanda Dobbyn,[1,2] Laura M. Huckins,[1,2] James Boocock,[3] Laura G. Sloofman,[1] Benjamin S. Glicksberg,[2,4,5] Claudia Giambartolomei,[6] Gabriel E. Hoffman,[1,2,4] Thanneer M. Perumal,[7] Kiran Girdhar,[1,2] Yan Jiang,[1,8] Towfique Raj,[2,4,9] Douglas M. Ruderfer,[10] Robin S. Kramer,[11] Dalila Pinto,[2,4,8,12,13] the CommonMind Consortium, Schahram Akbarian,[1,8] Panos Roussos,[1,4,8] Enrico Domenici,[14,15] Bernie Devlin,[16] Pamela Sklar,[1,2,4,8,17] Eli A. Stahl,[1,4,18,*] and Solveig K. Sieberts[7,18,*]

Causal genes and variants within genome-wide association study (GWAS) loci can be identified by integrating GWAS statistics with expression quantitative trait loci (eQTL) and determining which variants underlie both GWAS and eQTL signals. Most analyses, however, consider only the marginal eQTL signal, rather than dissect this signal into multiple conditionally independent signals for each gene. Here we show that analyzing conditional eQTL signatures, which could be important under specific cellular or temporal contexts, leads to improved fine mapping of GWAS associations. Using genotypes and gene expression levels from post-mortem human brain samples (n = 467) reported by the CommonMind Consortium (CMC), we find that conditional eQTL are widespread; 63% of genes with primary eQTL also have conditional eQTL. In addition, genomic features associated with conditional eQTL are consistent with context-specific (e.g., tissue-, cell type-, or developmental time point-specific) regulation of gene expression. Integrating the 2014 Psychiatric Genomics Consortium schizophrenia (SCZ) GWAS and CMC primary and conditional eQTL data reveals 40 loci with strong evidence for co-localization (posterior probability > 0.8), including six loci with co-localization of conditional eQTL. Our co-localization analyses support previously reported genes, identify novel genes associated with schizophrenia risk, and provide specific hypotheses for their functional follow-up.

Introduction

Significant advances in understanding the genetic architecture of schizophrenia (MIM: 181500) have occurred within the last 10 years. However, for common variants identified in genome-wide association studies (GWASs), the success in locus identification is not yet matched by an understanding of their underlying basic mechanism or effect on pathophysiology. Expression quantitative trait loci (eQTL), which are responsible for a significant proportion of variation in gene expression, could serve as a link between the numerous non-coding genetic associations that have been identified in GWASs and susceptibility to common diseases directly through their association with gene expression regulation.[1–4] Accordingly, results from eQTL mapping studies have been successfully utilized to identify genes and causal variants from GWASs for various complex phenotypes, including asthma (MIM: 600807), body mass index (MIM: 601665), celiac disease (MIM: 212750), and Crohn disease (MIM: 266600).[5–8]

Studies integrating eQTL and GWAS data have almost exclusively used marginal association statistics which typically represent the primary, or most significant, eQTL signal when assessing co-localization with GWASs, ignoring other SNPs that affect expression independently of the primary eQTL for a given gene. However, recent findings indicating that conditionally independent eQTL are widespread[9–12] motivate examination of the extent to which considering conditional eQTL may provide additional power to identify likely causal genes in a GWAS locus. Recent reports provide evidence that conditional eQTL are less frequently shared across tissues than primary

[1]Division of Psychiatric Genomics, Department of Genetics and Genomic Sciences, Icahn School of Medicine at Mount Sinai, New York, NY 10029, USA; [2]Department of Genetics and Genomic Sciences, Icahn School of Medicine at Mount Sinai, New York, NY 10029, USA; [3]Department of Human Genetics, David Geffen School of Medicine, University of California, Los Angeles, Los Angeles, CA 90024, USA; [4]Institute for Genomics and Multiscale Biology, Icahn School of Medicine at Mount Sinai, New York, NY 10029, USA; [5]Institute for Next Generation Healthcare, Mount Sinai Health System, Icahn School of Medicine at Mount Sinai, New York, NY 10029, USA; [6]Department of Pathology and Laboratory Medicine, University of California, Los Angeles, Los Angeles, CA 90095, USA; [7]Systems Biology, Sage Bionetworks, Seattle, WA 98109, USA; [8]Friedman Brain Institute, Icahn School of Medicine at Mount Sinai, New York, NY 10029, USA; [9]Department of Neuroscience, Icahn School of Medicine at Mount Sinai, New York, NY 10029, USA; [10]Division of Genetic Medicine, Department of Medicine, Psychiatry and Biomedical Informatics, Vanderbilt Genetics Institute, Vanderbilt University Medical Center, Nashville, TN 37235, USA; [11]Human Brain Collection Core, National Institute of Mental Health, Bethesda, MD 20892, USA; [12]Department of Psychiatry and Seaver Autism Center for Research and Treatment, Icahn School of Medicine at Mount Sinai, New York, NY 10029, USA; [13]Mindich Child Health and Development Institute, Icahn School of Medicine at Mount Sinai, New York, NY 10029, USA; [14]Laboratory of Neurogenomic Biomarkers, Centre for Integrative Biology (CIBIO), University of Trento, Trento, Italy; [15]The Microsoft Research - University of Trento Centre for Computational and Systems Biology (COSBI), Rovereto (TN), Italy; [16]Department of Psychiatry, University of Pittsburgh School of Medicine, Pittsburgh, PA 15213, USA
[17]Deceased November 20, 2017
[18]These authors contributed equally to this work
*Correspondence: eli.stahl@mssm.edu (E.A.S.), solly.sieberts@sagebase.org (S.K.S.)
https://doi.org/10.1016/j.ajhg.2018.04.011.
© 2018 The Authors. This is an open access article under the CC BY license (http://creativecommons.org/licenses/by/4.0/).

eQTL[10] and, like tissue- and cell type-specific eQTL, are often found more distally to the genes they regulate.[10,13,14] These lines of evidence suggest that conditionally independent eQTL may contribute to tissue-specific or other context-specific gene regulation (e.g., specific to a particular cell type, developmental stage, or stimulation condition). One mechanism by which disease risk could potentially be mediated by a conditional eQTL is the disruption of a tissue-specific enhancer by a given variant, leading to the dysregulation of the relevant eGene in only the tissue for which the enhancer is specific. For example, an eQTL affecting Parkinson disease risk through expression of *SNCA* was recently shown to act through the disruption of an enhancer;[15] if this enhancer is specific to a disease-relevant cell type, such as nerve cells of the substantia nigra, then it could manifest as a conditional eQTL since it would be only partially represented in brain homogenate.

Here, we leveraged genotype and dorsolateral prefrontal cortex (DLPFC) expression data provided by the CommonMind Consortium (CMC) to elucidate the role of conditional eQTL in the etiology of schizophrenia (SCZ). Currently comprising the largest existing postmortem brain genomic resource at nearly 600 samples, the CMC is generating and making publicly available an unprecedented array of functional genomic data, including gene expression (RNA sequencing), histone modification (chromatin immunoprecipitation [ChIP-seq]), and SNP genotypes, from individuals with psychiatric disorders as well as unaffected controls.[16] We utilized SNP dosage and RNA-sequencing (RNA-seq) data from the CMC to identify primary and conditionally independent eQTL. We then characterized the resulting eQTL on various genomic attributes including distance to transcription start site and their genes' specificities across tissues, cell types, and developmental periods. In addition, we quantified enrichment of primary and conditional eQTL in promoter and enhancer functional genomic elements inferred from epigenomic data. Finally, we isolated each independent eQTL signal by conducting a series of "all-but-one" conditional analyses for genes with multiple independent eQTL and then assessed the overlap between all eQTL association signals and the schizophrenia GWAS signals.

Material and Methods

CommonMind Consortium Data

We used pre-QC'ed genotype and expression data from the CommonMind Consortium, and detailed information on quality control, data adjustment, and normalization procedures can be found in Fromer et al.[16] Briefly, samples were genotyped at 958,178 markers using the Illumina Infinium HumanOmniExpress Exome array and markers were removed on the basis of having no alternate alleles, having a genotyping call rate ≤ 0.98, or having a Hardy-Weinberg p value $< 5 \times 10^{-5}$. After QC, 668 individuals genotyped at 767,368 markers were used for imputation. Phasing was performed on each chromosome using ShapeIt v2.r790,[17] and variants were imputed in 5 Mb segments with Impute v2.3.1[18] using the 1000 Genomes Phase 1 integrated reference panel,[19] excluding singleton variants. After phasing and imputation, then filtering out variants with INFO < 0.8 or MAF < 0.05, the number of markers included in the analysis totaled approximately 6.4 million. Gene expression was assayed via RNA-seq using 100 base pair paired end reads and was mapped to human Ensembl gene reference (v.70) using TopHat v.2.0.9 and Bowtie v.2.1.0. After discarding genes with less than 1 CPM (counts per million) in at least 50% of the samples, RNA-seq data for a total of 16,423 Ensembl genes was considered for analysis. The expression data was voom-adjusted for both known covariates (RIN, library batch, institution, diagnosis, post-mortem interval, and sex) and 20 surrogate variables identified via surrogate variable analysis (SVA).[20] After the removal of samples that did not pass RNA sample QC (including but not limited to: having RIN < 5.5, having less than 50 million total reads or more than 5% of reads aligning to rRNA, having any discordance between genotyping and RNA-seq data, and having RNA outlier status or evidence for contamination) and retaining only genetically identified European-ancestry individuals, a total of 467 samples was used for downstream analyses. These 467 individuals comprised 209 SCZ-affected case subjects, 52 AFF (bipolar, major depressive disorder, or mood disorder, unspecified)-affected case subjects, and 206 control subjects.

eQTL Identification

An overview of our workflow can be found in Figure S1. First, to identify primary and conditional *cis*-eQTL, we a conducted forward stepwise conditional analysis implemented in MatrixEQTL[21] using genotype data at 6.4 million markers and RNA-seq data for 16,423 genes. FDR was initially assessed using the Benjamini-Hochberg algorithm across all *cis*-eQTL tests within each chromosome. FDR was not re-assessed at each conditional step; instead, a fixed p value threshold was used as the inclusion criteria in the stepwise model selection. For each gene with at least one *cis*-eQTL (gene $\pm$ 1 Mb) association at a 5% false discovery rate (FDR), the most significant SNP was added as a covariate in order to identify additional independent associations (considered significant if the p value achieved was less than that corresponding to the initial 5% FDR for primary eQTL). This procedure was repeated iteratively until no further eQTL met the p value threshold criteria. We used a linear regression model, adjusting for diagnosis and five ancestry covariates inferred by GemTools. Following eQTL identification, only autosomal eQTL were retained for downstream analyses.

Replication in Independent Datasets

Replication was performed in the HBCC microarray cohort (dbGaP: phs000979, see Web Resources) and in the ROSMAP[22] RNA-seq cohort by fitting the stepwise regression models identified in the CMC data. For cases in which a marker was unavailable in the replication cohort, all models including that marker (i.e., for that eQTL and higher-order eQTL conditional on it, for a given gene) were omitted from replication.

Data from the HBCC cohort was QC'ed and normalized as described in Fromer et al.[16] DLPFC tissue was profiled on the Illumina HumanHT-12_V4 BeadChip and normalized in an analogous manner to the CMC data. Genotypes were obtained using the HumanHap650Yv3 or Human1MDuov3 chips and imputed using the 1000 Genomes Phase 1 reference panel. Replication of the eQTL models was performed on 279 genetically inferred European-ancestry samples (76 control subjects, 72 SCZ-affected

subjects, 43 BP-affected subjects, 88 MDD-affected subjects), adjusting for diagnosis and five ancestry components.

ROSMAP data were obtained from the AMP-AD Knowledge Portal (see Web Resources). Quantile normalized FPKM expression values were adjusted for age of death, RIN, PMI, and 31 hidden confounders from SVA, conditional on diagnosis. Only genes with FPKM > 0 in more than 50 samples were retained. QC'ed genotypes were also obtained from the AMP-AD Knowledge Portal and imputed to the Haplotype Reference Consortium (v.1.1)[23] reference panel via the Michigan Imputation Server.[24] Only markers with imputation quality score $R^2 \geq 0.7$ were considered in the replication analysis. GemTools was used to infer ancestry components as was done for the CMC data above. After QC, 494 samples were used for eQTL replication in a linear regression model that also adjusted for diagnosis (Alzheimer disease, mild cognitive impairment, no cognitive impairment, and other) and four ancestry components.

Modeling Number of eQTL per Gene on Genomic Features

We considered three genomic features (gene length, number of LD blocks in the *cis*-region, and genic constraint score) for our modeling analyses. Gene lengths were calculated using Ensembl gene locations. We obtained LD blocks from the LDetect Bitbucket site to tally the number of blocks overlapping each gene's *cis*-region (gene ± 1 Mb). We obtained loss-of-function-based genic constraint scores from the Exome Aggregation Consortium (ExAC). A negative binomial generalized linear regression model was used to model the number of eQTL per gene based on the above variables; results were qualitatively the same using linear regression of Box-Cox transformed eQTL numbers. Backward-forward stepwise regression using the full model with interaction terms for these three variables was used to determine the relationship between genomic attributes and eQTL number. These analyses were implemented in R. *cis*-heritability of gene expression was estimated using the same CMC data that were used for eQTL detection, including all markers in the *cis*-region and implemented in GCTA.[25] SNP-heritability estimates were then added to the modeling procedure described above.

Tissue, cell type, and developmental time point specificity were measured using the expression specificity metric Tau.[26,27] Tissue specificity for each gene was calculated using publicly available expression data for 53 tissues from the GTEx project[28] (release V6p). Expression for each tissue was summarized as the log2 of the median expression plus one, and then used to calculate tissue specificity Tau. Cell type specificity for each gene was computed using publicly available single-cell RNA-sequencing expression data[29] generated from human cortex and hippocampus tissues. Raw expression counts for 285 cells comprising six major cell types of the brain were obtained from GEO (GSE67835) and counts data were library normalized to CPM. Expression for each cell type was summarized as the log2 of the mean expression plus one, and then used to compute cell type specificity Tau. Developmental time point specificity for each gene was calculated using publicly available DLPFC expression data for 27 time points, clustered into eight biologically relevant groups, from the BrainSpan atlas (see Web Resources). Eight developmental periods[30] were defined as follows: early prenatal (8–12 pcw), early mid-prenatal (13–17 pcw), late mid-prenatal (19–24 pcw), late prenatal (25–37 pcw), infancy (4 months–1 year), childhood (2–11 years), adolescence (13–19 years), and adulthood (21+ years). Expression for each time point was summarized as the log2 of the median expression plus one and then used to calculate developmental period specificity Tau. Each Tau was added to the above model for eQTL number individually, as well as all together.

Enrichment Analyses

We divided eQTL into separate subgroups by stepwise conditional order (first, second, and greater than second) and created sets of matched SNPs drawn from the SNPsnap[31] database for each subgroup, matching on minor allele frequency, gene density (number of genes within 1 Mb of the SNP), distance from SNP to TSS of the nearest gene, and LD (number of LD-partners within $r^2 \geq 0.8$). For each subgroup of eQTL, we performed a logistic regression of status as eQTL or matched SNP on overlap with functional annotation, including the four SNP matching parameters as covariates. Enrichment was taken as the regression coefficient estimate, interpretable as the log-odds ratio for being an eQTL given a functional annotation. Functional annotations tested included: brain promoters and enhancers (union of all brain region TssA and Enh+EnhG intervals, respectively, from the NIH Roadmap Epigenomics Project[32] ChromHMM[33] core 15-state model), brain-specific promoters and enhancers (the union of all brain region TssA and Enh+EnhG intervals, excluding those present in seven other non-brain tissues/cell types: primary T helper cells from peripheral blood, osteoblast primary cells, HUES64 cells, adipose nuclei, liver, NHLF lung fibroblast primary cells, and NHEK-epidermal keratinocyte primary cells), and pre-frontal cortex (PFC) neuronal ($NeuN^+$) and non-neuronal ($NeuN^-$) nucleus H3K4me3 and H3K27ac ChIP-seq marks from the CMC. For each data source, active promoter and enhancer (or H3K4me3 and H3K27ac) annotations were tested for enrichment jointly. This analysis was repeated but restricting to matched SNPs located within 1 Mb of any of the 16,423 genes that were tested for eQTL, in order to determine whether the enrichment estimates were inflated due to the proximity of our primary and conditional eQTL to brain-expressed genes, which may be more likely to occur near active regulatory regions in the brain. In addition, to ensure that any enrichment patterns observed were not due to varying effect size among primary and conditional eQTL, the enrichment analyses were also carried out taking into account the variance in expression explained by each eQTL. Variance explained (R^2) was estimated using the variancePartition[34] R package, and eQTL were stratified into three R^2 bins: bin 1, $1 \times 10^{-2} \leq R^2 \leq 1.75 \times 10^{-2}$; bin 2, $1.75 \times 10^{-2} \leq R^2 \leq 2.25 \times 10^{-2}$; and bin 3, $2.25 \times 10^{-2} \leq R^2 \leq 3 \times 10^{-2}$. Logistic regression of status as eQTL or matched SNP was then carried out separately for each R^2 bin, within each eQTL order.

Conditional eQTL Analyses

In order to isolate each conditionally independent *cis*-eQTL association, we carried out a series of "all-but-one" conditional analyses, implemented within MatrixEQTL,[21] for each gene possessing more than one independent eQTL. As these conditional eQTL signals were to be used to test for co-localization with the SCZ GWAS signals, we limited these analyses to those genes (346 in total) with eQTL overlapping GWAS loci. For each of these genes, we conducted an all-but-one analysis for each independent eQTL by regressing the given gene's expression data on the dosage data, including all of the other independent eQTL for that gene as covariates in addition to diagnosis and five ancestry components. For example, three conditional analyses would be conducted for a

gene with three independent eQTL: one analysis conditioning on the secondary and tertiary eQTL, one analysis conditioning on the primary and tertiary, and one analysis conditioning on the primary and secondary. In this manner we generated summary statistics for each independent eQTL in isolation, conditional on all of the other independent eQTL for that gene.

Co-localization Analyses

For our co-localization analyses, we used summary statistics and genomic intervals from the 2014 Psychiatric Genomics Consortium (PGC) SCZ GWAS.[35] We included 217 loci at a p value threshold of 1×10^{-6} (excluding the MHC locus), defined these loci by their LD $r^2 \geq 0.6$ with the lead SNP, and then merged overlapping loci. GWAS and eQTL signatures were qualitatively compared using p value-p value (P-P) plots, rendered in R, and LocusZoom[36] plots.

Multiple methods that aim to identify GWAS-eQTL co-localized loci are currently available.[37–42] We chose to further develop coloc[39] for our co-localization analyses for several reasons: (1) it uses data from all SNPs within a locus; (2) it avoids the computational burden or approximate results of Bayesian inferential methods for causal variants,[41,42] which rely on reference panel estimates of linkage disequilibrium (LD); and (3) and it has been widely used[43–45] including in direct comparisons of GWAS-eQTL co-localization methods.[42,46] We tested for co-localization using an updated version of coloc[39] R functions, which we name coloc2 (see Web Resources), and incorporated several improvements to the method. First, coloc2 pre-processes data by aligning eQTL and GWAS summary statistics for each eQTL *cis*-region. Second, the coloc2 model optionally incorporates changes implemented in gwas-pw.[43] Briefly, we implemented likelihood estimation of mixture proportions of five hypotheses (H_0, no association; H_1, GWAS association only; H_2, eQTL association only; H_3, both but not co-localized; and H_4, both and co-localized) from genome-wide data. Coloc2 uses these proportions as priors (or optionally, coloc default or user-specified priors) in the empirical Bayesian calculation of the posterior probability of co-localization for each locus (eQTL *cis*-region). Coloc2 averages per-SNP Wakefield asymptotic Bayes factors (WABF)[47] across three different values for the WABF prior variance term, 0.01, 0.1, and 0.5, and provides options for specifying phenotypic variance, estimating it from case-control proportions or estimating it from the data.

Results

Identification of eQTL

Primary and conditional eQTL were identified using genotype and RNA-seq data from the CommonMind Consortium post-mortem DLPFC samples (467 European-ancestry case and control subjects).[16] We identified 12,813 primary and 16,082 conditional eQTL, totaling 28,895 independent eQTL. Of the genes tested, 81% (12,813 of 15,817 autosomal genes) had at least one eQTL and 63% of these (51% of all genes) also had at least one conditional eQTL, with an average of 1.83 independent eQTL per gene (2.26 among those with at least one eQTL) (Figure 1A). Conversely, when examining the distributions for the number of genes whose expression was affected by each eQTL (Table S1), the majority of eQTL were specific for a single gene, and only a small fraction of eQTL, 1.47%, affected more than one gene, with a maximum of six genes affected by a single eQTL.

We tested for replication of conditional eQTL in two independent datasets, the National Institute of Mental Health's Human Brain Collection Core (HBCC, n = 279, microarray expression data) and the Religious Orders Study/Memory and Aging Project[22] (ROSMAP, n = 494, RNA-seq expression). For each gene the same models were evaluated that were identified in forward-stepwise conditional analysis in the CMC data. We observed significant evidence of replication for both primary and conditional eQTL in the HBCC and ROSMAP post-mortem brain cohorts (Table S2). The estimated proportion of true associations (π_1) in ROSMAP was 0.57 and 0.26 for primary and conditional eQTL, respectively; in HBCC π_1 was 0.46 and 0.20 for primary and conditional eQTL. Therefore, replication was stronger for primary than for conditional eQTL, as expected given their stronger effect sizes. Replication rates were somewhat higher in the RNA-seq ROSMAP data than in HBCC.

Genomic Characterization of Primary and Conditional eQTL

The features for which primary and conditional eQTL and their respective eGenes displayed identifiable differences included distance from eQTL to its gene's transcription start site (TSS), gene length, LD blocks per genic *cis*-region, genic constraint score, and genic *cis*-SNP-heritability. According to prior results, eQTL that are shared across tissues and cell types tend to be located closer to transcription start sites than context-specific eQTL;[13,14] we therefore first examined the relationship between primary or conditional eQTL status and distance to genic TSS. Primary eQTL fall closer to the TSS than conditional eQTL (Figure 1C): primary eQTL occur at a median distance of 70.4 kb from the TSS versus a median distance of 302 kb for conditional eQTL. This difference holds true even more proximally to the TSS (Figure S2); 8.1% and 2.5% of primary and conditional eQTL, respectively, fall within 3 kb of the TSS. We next characterized the relationship between the number of independent eQTL per gene and three different genomic features: gene length, number of LD blocks[48] in the gene's *cis*-region (±1 Mb), and Exome Aggregation Consortium (ExAC) genic constraint score,[49] including possible interactions. The best multivariate model for eQTL number included gene length, number of LD blocks, and genic constraint as predictors, as well as a gene length-LD blocks interaction (Table 1). The number of independent eQTL was positively correlated with gene length and number of LD blocks and negatively correlated with genic constraint score (Figure S3). We then examined the variance of gene expression explained by *cis*-region SNPs, or *cis*-SNP-heritability, estimated by linear mixed model variance component analysis[25] (Figure S4). We found a strong effect of estimated *cis*-heritability on number of independent eQTL (Table 1, Figure S5). In a joint model with

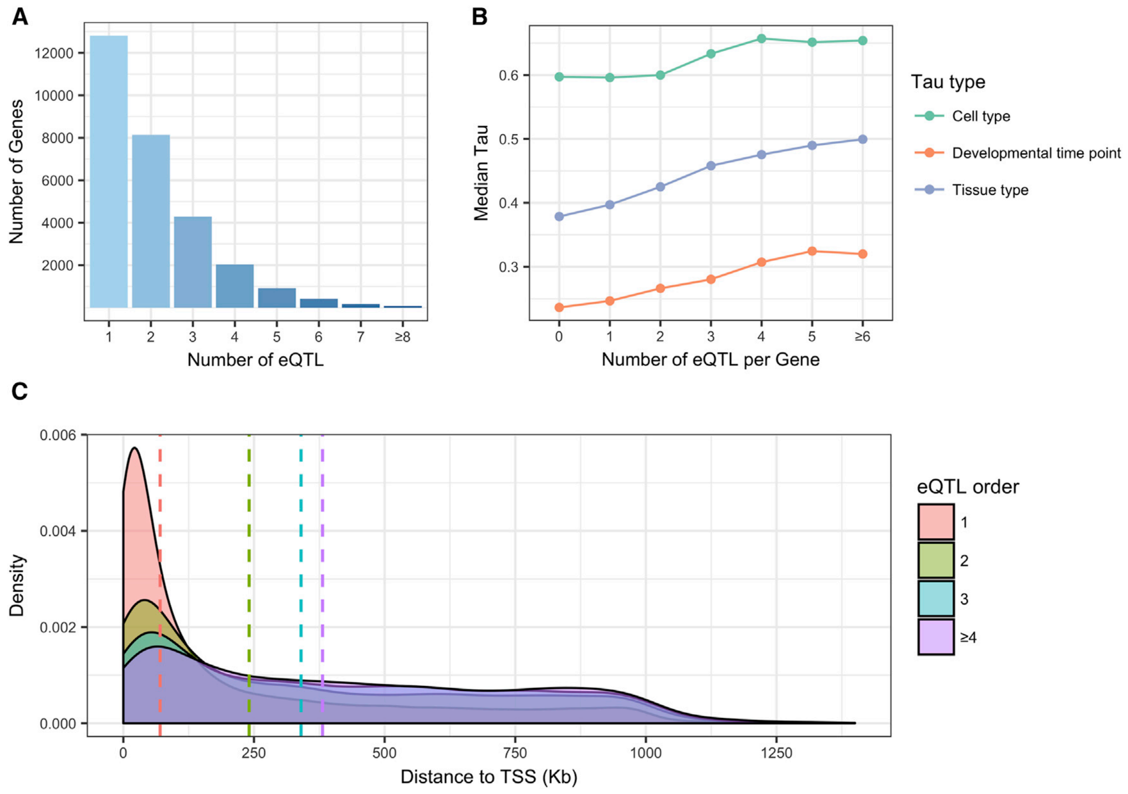

Figure 1. Characterization of Conditional eQTL

(A) Counts of the numbers of genes (y axis) regulated by at least N ($1 \leq N \leq 16$) independent eQTL (x axis).

(B) Median Tau value (y axis) for genes with N independent eQTL (x axis), colored by Tau type (cell type, developmental time point, or tissue type Tau).

(C) Density plot representing the distance from eSNP to eGene transcription start site (TSS), colored by eQTL order. Dashed lines represent the median distance to TSS for each order of eQTL.

cis-SNP-heritability, the main effects of gene length, number of LD blocks, and genic constraint on eQTL number remained at least nominally significant.

We then addressed whether genes with conditional eQTL exhibit greater context specificity as measured by the robust expression specificity metric Tau.[26,27] We calculated Tau across 53 tissues from the Genotype-Tissue Expression (GTEx) project, across 6 DLPFC cell types (astrocytes, endothelial cells, microglia, neurons, oligodendrocytes, and oligodendrocyte progenitor cells) from single-cell RNA-seq,[29] and across 8 developmental periods[30] (early prenatal, early mid-prenatal, late mid-prenatal, late prenatal, infant, child, adolescent, and adult) from the BrainSpan atlas DLPFC RNA-seq data. We confirmed that higher values of Tau reflect expression specificity by comparing the distributions of all three Tau measures for all genes with the distributions for a subset of housekeeping genes[50] (Figure S6). We found positive correlations between eQTL number and tissue, cell type, and developmental time point specificities (Figure 1B, Table 1, Table S3, Figure S7). In a joint model, the strongest correlation was with DLPFC cell type Tau, which is consistent with previous data demonstrating tissue-specific, cell type-dependent expression in blood;[12] however, we note that all three Tau sets were inter-correlated (Table S3).

Epigenetic Enrichment Analyses

One way in which eQTL may affect gene expression is through alteration of *cis*-regulatory elements such as promoters and enhancers. Putative causal eSNPs have been shown to be enriched in genomic regions containing functional annotations such as DNase hypersensitive sites, transcription factor binding sites, promoters, and enhancers.[51–54] Our observation that conditional eQTL fall farther from transcription start sites than primary eQTL led us to hypothesize that primary eQTL may affect transcription levels by altering functional sites in promoters whereas conditional eQTL may do so by altering more distal regulatory elements such as enhancers. We therefore assessed enrichment of primary and conditional eQTL in brain active promoter (TssA) and enhancer (merged Enh and EnhG) states derived from the NIH Roadmap Epigenomics Project,[32,33] and in H3K4me3 and H3K27ac neuronal (NeuN^{+}) and non-neuronal (NeuN^{-}) ChIP-seq peaks from a subset of the CMC post-mortem DLPFC samples. The overlap of H3K4me3 and H3K27ac ChIP-seq peaks was used as a proxy for active promoters, and H3K27ac peaks that do not overlap H3K4me3 peaks were used as a (relatively non-specific) proxy for enhancers.[33] We performed logistic regression of SNP status (eQTL versus random matched SNP) on overlap with functional

Table 1. **Number of eQTL per Gene Modeled on Genomic Features**

Predictor	Model 1 Estimate	Model 1 Robust SE	Model 1 Pr(> |z|)	Model 2 Estimate	Model 2 Robust SE	Model 2 Pr(> |z|)	Model 3 Estimate	Model 3 Robust SE	Model 3 Pr(> |z|)
log(Gene length)	0.27	0.04	5.16E−12	0.16	0.03	2.20E−06	0.17	0.03	9.87E−07
LD blocks	0.59	0.17	6.47E−04	0.33	0.15	2.92E−02	0.37	0.15	1.55E−02
log(Gene length): LD blocks	−0.03	0.02	7.77E−02	−0.01	0.01	5.65E−01	−0.01	0.01	4.11E−01
Constraint	−0.61	0.03	5.93E−85	−0.20	0.03	2.93E−13	−0.15	0.03	5.41E−08
cis-heritability	–	–	–	7.03	0.18	0.00	7.02	0.18	0.00
Tau (tissue)	–	–	–	–	–	–	0.08	0.08	2.76E−01
Tau (DLPFC cell type)	–	–	–	–	–	–	0.20	0.09	3.69E−02
Tau (developmental time point)	–	–	–	–	–	–	0.17	0.09	5.99E−02

annotations, separately for each eQTL order (primary, secondary, and greater than secondary).

Primary and conditional eQTL were significantly enriched in both promoter and enhancer chromatin states from REMC brain and CMC DLPFC tissues, with greatest enrichments overall observed in PFC neuronal (NeuN$^+$) promoters and enhancers (Figure 2, Table S4). We found that whereas active promoter enrichments in all tissue/cell types markedly decreased with higher conditional order of eQTL, enhancer enrichments either only slightly decreased (REMC brain and PFC NeuN$^+$, Figures 2A and 2C) or remained level (REMC brain-specific, Figure 2B). Though there was also significant enrichment of eQTL in non-neuronal nuclei (NeuN$^-$) promoters and enhancers, this trend of a marked decrease in active promoters but steady levels of enhancer enrichment with greater eQTL order was not observed for non-neuronal PFC nuclei (Figure 2D). This greater decrease in enrichment for promoters compared to enhancers with increasing eQTL order was not confounded by an excess of eQTL near brain-expressed genes in comparison to matched SNPs (Figure S8, Table S5) and furthermore was not an artifact of varying effect size with eQTL order; the same overall pattern was observed when stratifying eQTL by variance in expression explained (R^2) and comparing enrichment across eQTL order, within each R^2 bin (Figures S9–S12, Table S6).

eQTL Co-localization with SCZ GWAS

We performed co-localization analyses in order to evaluate the extent of overlap between eQTL and GWAS signatures in schizophrenia and to identify putative causal genes from GWAS associations. Considering 217 loci (Table S7) with lead SNPs reaching a significance threshold of $p < 1 \times 10^{-6}$ from the 2014 Psychiatric Genomics Consortium (PGC) schizophrenia GWAS,[35] we tabulated the number of primary and conditional eQTL falling within GWAS loci. A total of 114 out of 217 loci contained primary and/or conditional eQTL for 346 genes; 110 of these genes had one eQTL only and 236 genes had more than one independent eQTL.

To quantitatively compare the SCZ GWAS and eQTL association signatures, we modified the R package coloc[39] for Bayesian inference of co-localization between the two sets of summary statistics across each gene's *cis*-region. Coloc2, our modified implementation of coloc, analyzes the hierarchical model of gwas-pw,[43] with likelihood-based estimation of dataset-wide probabilities of five hypotheses (H_0, no association; H_1, GWAS association only; H_2, eQTL association only; H_3, both but not co-localized; and H_4, both and co-localized). We then used these probabilities as priors to calculate empirical Bayesian posterior probabilities for the five hypotheses for each locus, in particular PPH_4 for co-localization.

For genes with conditional eQTL overlapping SCZ GWAS loci, summary statistics from all-but-one conditional eQTL analyses were assessed for co-localization with the GWAS signature (Figure 3). To illustrate this analytical strategy, we show eQTL results for the iron responsive element binding protein 2 gene *IREB2* (MIM: 147582, chr15:78729773–78793798) as an example (Figure 4). Forward stepwise selection analysis identified two independent *cis*-eQTL for *IREB2*. In order to generate summary statistics for each eQTL in isolation, we conducted two all-but-one conditional analyses, in each analysis conditioning on all but a focal independent eQTL (for *IREB2* this entailed conditioning on only one eQTL per conditional analysis, but involved conditioning on up to six eQTL per gene across all genes considered in the SCZ co-localization analysis). We then tested for co-localization between the GWAS and all of the eQTL summary statistics resulting from the above conditioning analysis using coloc2 (Table S12). In the case of *IREB2*, the conditional eQTL (rs7171869) was implicated as co-localized with the GWAS signal at this locus with a posterior probability for co-localization (PPH_4) of 0.94. A qualitative examination of the *IREB2* locus supported the coloc2 results: the correlation between the GWAS p values and conditional eQTL p values was higher than that between the GWAS and primary eQTL p values (Figure 4A). In addition, the GWAS signature for the locus more closely resembled the

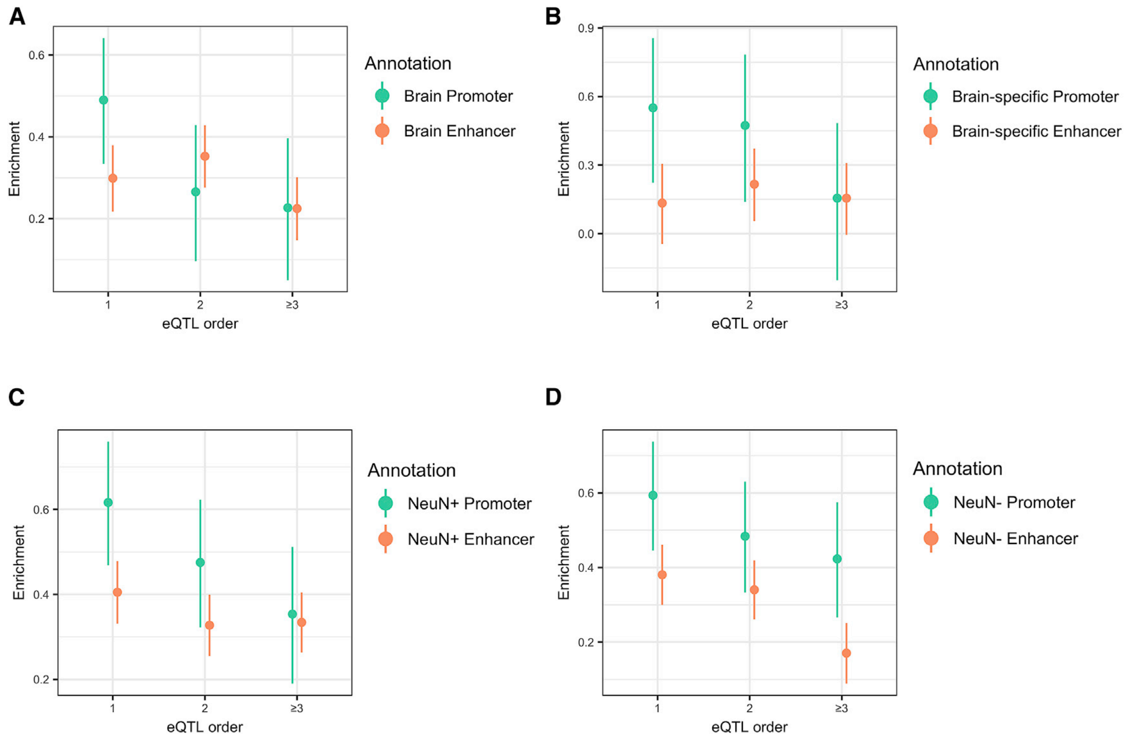

Figure 2. Enrichments of Primary and Conditional eQTL in Active Regulatory Annotations

Plotted are enrichments (regression coefficient estimate ± 95% CI from logistic regression, y axes) of primary (x axis eQTL order = 1) and conditional (eQTL order = 2, ≥ 3) eQTL in functional annotations.

(A and B) Enrichment in brain (union of all individual brain regions) and brain-specific (present in brain but not in seven other non-brain tissues) active promoter (green) and enhancer (orange) ChromHMM states from the NIH Roadmap Epigenomics Project.

(C) Enrichment in neuronal nuclei ($NeuN^+$) for active promoters (intersection of DLPFC H3K4me3 and H3K27ac ChIP-seq peaks, green) and enhancers (H3K27 peaks that do not overlap H3K4me3 peaks, orange).

(D) Enrichments in the same annotations, but for DLPFC non-neuronal nuclei ($NeuN^-$).

conditional eQTL signature than either the non-conditional eQTL signature or the primary eQTL signature (Figure 4B).

We found that 40 loci contained genes with strong evidence of co-localization between eQTL and GWAS signatures, with posterior probability of H_4 (PPH_4) ≥ 0.8 (Table 2). When restricting to genome-wide significance for the GWAS, we found co-localization in 24 of the 108 loci. Given the correlations between number of independent eQTL and expression specificity scores (Tau) across tissues, cell types, and development, we tabulated the reported genes' Tau percentiles and expression levels, to highlight contexts in which the genes are specifically expressed (Table 2, Table S8). We acknowledge that while posterior probability PPH_4 ≥ 0.8 demonstrates strong Bayesian evidence for co-localization, it is an arbitrary threshold for characterizing loci as GWAS-eQTL co-localized; we find that many loci with PPH_4 ≥ 0.5 appear qualitatively consistent with co-localization.

Importantly, for 6 of the 40 co-localizing loci, a conditional rather than primary eQTL co-localized with the GWAS with compelling qualitative support (Table 2, Figure 4, Table S11, Figures S13–S17). The genes showing strong evidence for conditional eQTL co-localization include *SLC35E2*, *PROX1-AS1* (MIM: 601546), *PPM1M* (MIM: 608979), *SDAD1P1*, *STAT6* (MIM: 601512), and *IREB2*. Also notable are the occurrences of complex patterns of co-localization for some loci; for example, three loci showed evidence for co-localization with a primary eQTL for one gene and a conditional eQTL for another.

Comparison with Previous Co-localization Analyses

In the prior CMC study, a GWAS-eQTL co-localization analysis implemented in Sherlock and using non-conditional eQTL summary statistics reported a total of 18 co-localized loci, representing 17% of the 108 genome-wide significant loci examined. Through our all-but-one conditional co-localization analysis, we replicate the majority of their findings and detect an additional 13 instances of co-localization, bringing the total number of co-localizations when considering only the genome-wide significant (and not including the MHC) loci up to 24 (representing 22% of these 108 loci) (Table S9). These 13 comprise instances of conditional eQTL co-localization (for genes *SLC35E2* and *IREB2*) and improved detection of primary eQTL co-localization due to isolation of independent eQTL signatures and our choice of co-localization software (coloc2). Of the six co-localized loci identified in the previous but not current analysis, three resulted from differences in study design such as GWAS locus definition and

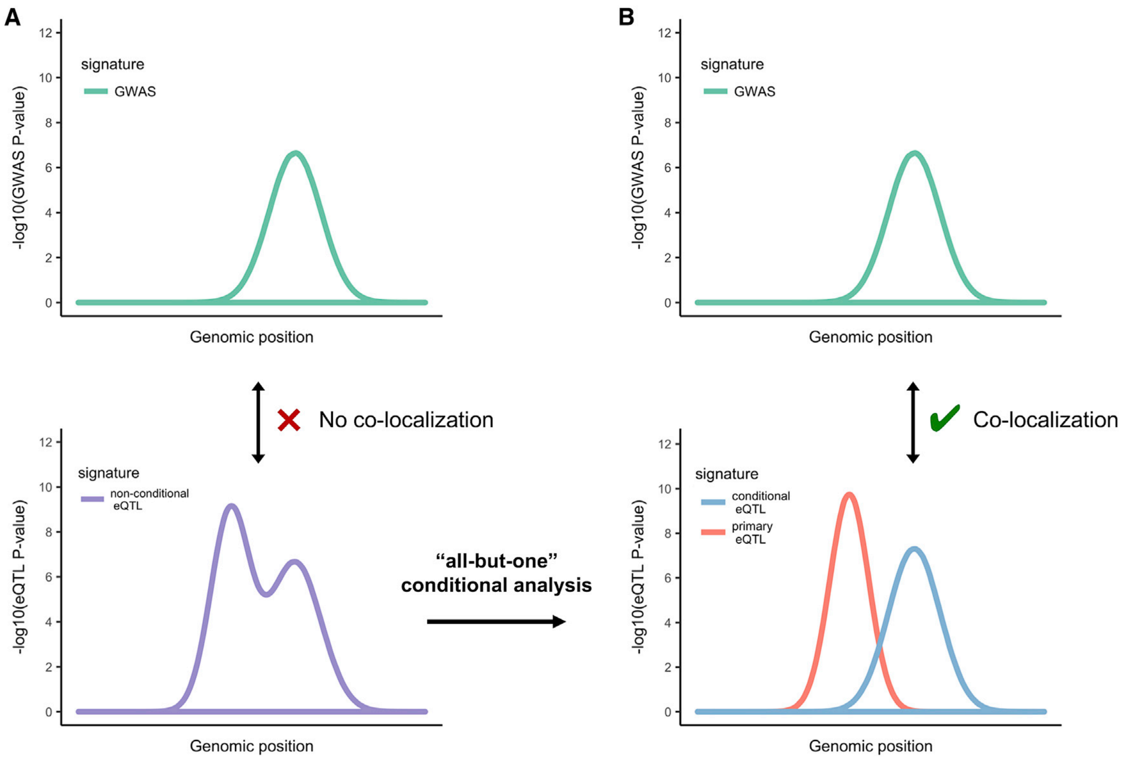

Figure 3. All-but-One Conditional Analysis to Isolate Independent eQTL Signatures
(A) Hypothetical GWAS signature (top, green) at a given locus and an overlapping hypothetical eQTL signature (bottom, purple), which comprises two independent eQTL.
(B) Same hypothetical GWAS and eQTL signatures after the all-but-one conditional eQTL analysis isolating the primary (red) and secondary (blue) eQTL signatures. Before conditional analysis there is a lack of co-localization between the GWAS signature and eQTL signature. After all-but-one conditional analysis, there is evidence for co-localization between the conditional (secondary) eQTL and GWAS signatures.

eQTL overlap criteria, and two were suggestive in the current analysis ($0.65 < PPH_4 < 0.8$). The one remaining discrepant locus (chr8:143302933–143403527) was found to co-localize with *TSNARE1* eQTL previously (Sherlock $p = 8.24 \times 10^{-7}$) but not here (coloc2 primary eQTL $PPH_4 = 0.074$, $PPH_3 = 0.93$). A qualitative comparison of the eQTL and GWAS data (Figure S18) did not appear to support co-localization; while the strongest GWAS association and the strongest eQTL are in close physical proximity, the LD between the two index SNPs is low ($r^2 \sim 0.2$–0.4). Additionally, our attempts to disentangle independent eQTL signal via conditional analysis do not reveal the GWAS index SNP to be in high LD with any of the conditionally independent eQTL peaks.

We also compared our conditional co-localization results with those from non-conditional eQTL analysis, using coloc2 and the same SCZ GWAS loci (Table S10). Conditional and non-conditional coloc2 results were highly concordant, with slightly higher PPH_4s resulting from the same WABFs due to a higher prior probability of co-localization estimated in the non-conditional coloc2 analysis. Thirty-five loci were co-localized in both analyses; five loci that were co-localized in the non-conditional analysis only were highly suggestive in the conditional analysis ($0.65 < PPH_4 < 0.8$), and the five loci that were co-localized only in the conditional coloc2 analysis involved conditional eQTL, emphasizing the utility of the conditional analysis. This conditional eQTL co-localization represents a substantial proportion (~15%) of all instances of co-localization, and furthermore could reflect context-specific differential expression that has the potential to implicate cell types, tissue types, and developmental stages that are relevant to disease etiology.

Discussion

We utilized genotype and expression data from 467 human post-mortem brain samples from the DLPFC to conduct eQTL mapping analyses, to characterize both primary and conditional eQTL. We then identified co-localization between SCZ GWAS and eQTL association signals, comprising both primary and conditional eQTL. Our principal findings include four major observations. First, we detect that conditional eQTL are widespread in the brain tissue samples we investigated. In 63% of genes with at least one eQTL, we found multiple statistically independent eQTL (representing 8,136 genes). In addition, conditional eQTL make substantial contributions to regulatory genetic variation, as there is a strong association between

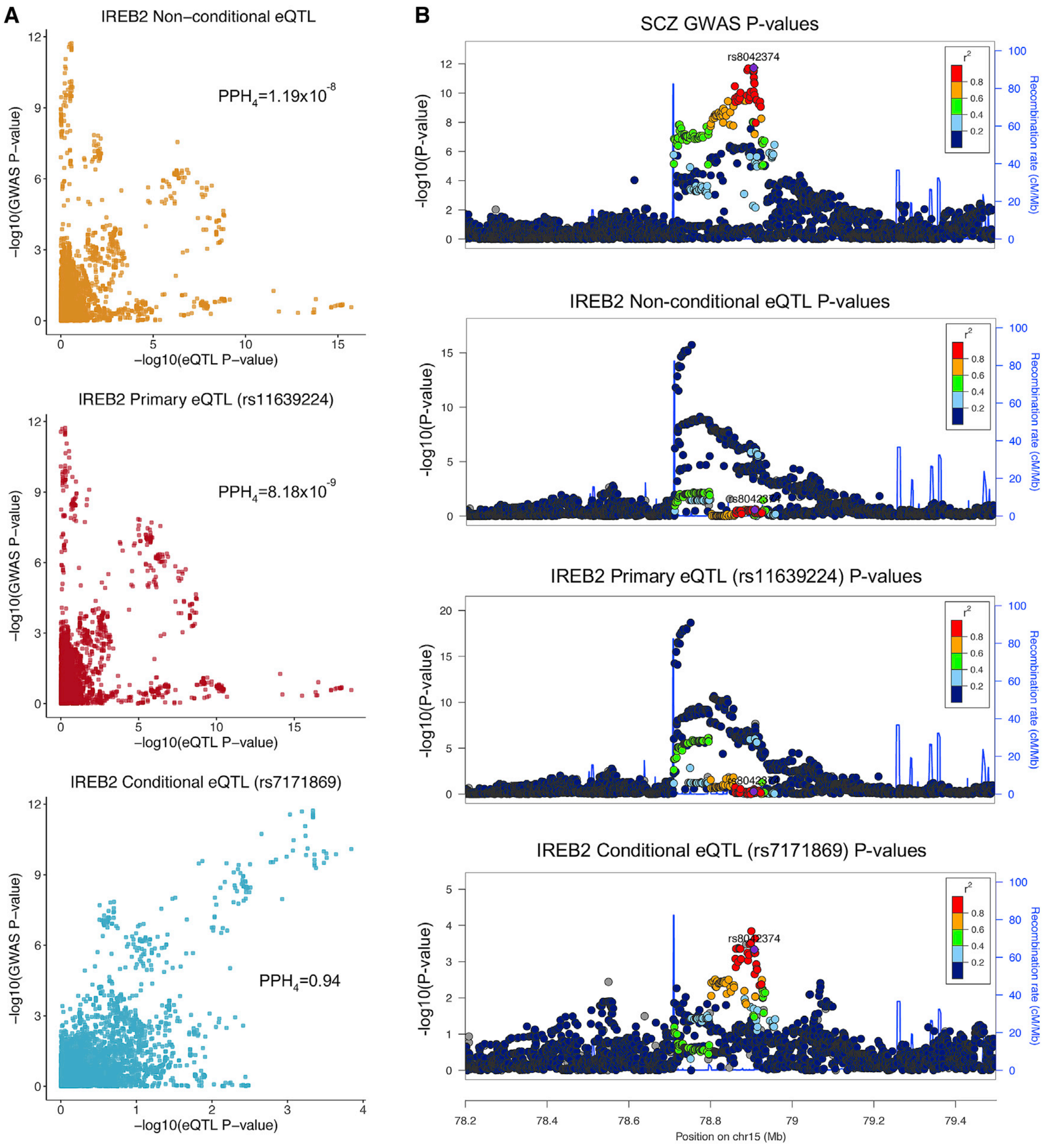

Figure 4. GWAS Signature for *IREB2* Co-localizes with the Conditional eQTL Signature
(A) P-P plots comparing $-\log_{10}$ p values from GWAS (y axes) and all-but-one conditional eQTL analysis (x axes), which show the highest correlation to be between the GWAS and the conditional eQTL rs7171869 (blue, bottom).
(B) LocusZoom plots for the *IREB2* locus, where the GWAS signal (top) more closely resembles the conditional eQTL signal (rs7171869, bottom) than the primary eQTL signal (rs11639224, third from top) or non-conditional eQTL signal (second from top). For all LocusZoom plots, LD is colored with respect to the GWAS lead SNP (rs8042374, labeled).

eQTL number and gene expression *cis*-SNP-heritability. This demonstrates that genetic variation affecting RNA abundance is incompletely characterized by focusing on only one primary eQTL per gene, which is the case currently for most eQTL studies.

Second, we find the genomics of conditional eQTL and their genes are consistent with complex, context-specific regulation of gene expression, which may be conferred through overlap with distal regulatory elements. Genes with more independent eQTL tend to be larger and span multiple recombination hotspot intervals, and tend to be less constrained at the protein level. While these associations may reflect in part greater power to detect independent eQTL that are not in linkage disequilibrium and explain more phenotypic variance, they are also consistent with more complex regulation and greater potential for regulatory genetic variation. Context-specific genetic regulation of expression could manifest as conditional eQTL signal in the analysis of expression from a heterogeneous source. For example, eQTL in naive and stimulated (LPS, IFN) monocytes[55] may occur as either primary or conditional eQTL in our CMC data, due to related microglial cells

Table 2. GWAS-eQTL Co-localized Loci

Chr	GWAS Locus Start	GWAS Locus End	GWAS Lead SNP	GWAS p Value	eSNP	eSNP p Value	Primary/ Conditional	PPH_4	Gene	Relevant Tissue/Cell Type/ Developmental Period
1	2372401	2402501	rs4648845	4.03E−09	rs12037821	4.9E−04	conditional	0.87	*SLC35E2*	–/–/early mid-prenatal
1	8355697	8638984	rs301797	2.03E−09	rs138050288	1.8E−04	primary	0.95	*RERE*	–/–/–
1	30412551	30443951	rs1498232	1.28E−09	rs2015244	1.8E−08	primary	0.99	*PTPRU*	–/neurons /early mid-prenatal
1	163582923	163766623	rs7521492	5.64E−07	rs10799961	3.18E−11	primary	0.91	*PBX1*	–/–/early prenatal
1	205015255	205189455	rs16937	8.69E−07	rs12724651	7.31E−07	primary	0.89	*TMEM81*	–/neurons/–
					rs12031350	8.15E−06	conditional	0.87	*RBBP5*	–/–/–
1	214137889	214163689	rs7529073	9.69E−07	rs1431983	1.67E−04	conditional	0.93	*PROX1-AS1*	cerebellar hemisphere/neurons/adult
2	73194203	73900439	rs56145559	8.42E−08	rs11679809	1.85E−34	primary	0.86	*ALMS1P*	testis/–/–
2	110262036	110398236	rs9330316	7.69E−08	rs892464	2.35E−26	primary	0.92	*SEPT10*	–/–/late prenatal
2	198148577	198835577	rs6434928	1.48E−11	rs12621129	6.06E−12	primary	0.94	*SF3B1*	–/–/–
2	200715237	201247789	rs281768	1.78E−14	rs35220450	3.46E−14	primary	0.95	*FTCDNL1, AC073043.2*	–/–/adult
					rs186546506	8.77E−04	conditional	0.83	*LINC01792, AC007163.3*	putamen (basal ganglia)/ –/adult
2	208371631	208531731	rs2709410	5.75E−07	rs34171849	5.86E−17	primary	0.88	*METTL21A*	–/–/–
					rs2551656	2.85E−09	primary	0.86	*CREB1*	–/–/early prenatal
2	220033801	220071601	rs6707588	9.51E−07	rs13404754	1.08E−09	primary	0.92	*CNPPD1*	–/–/–
3	36843183	36945783	rs75968099	3.39E−12	rs9834970	1.88E−05	primary	0.94	*DCLK3*	nerve - tibial /neurons/infant
3	52281078	53539269	rs2535627	3.96E−11	rs6801235	2.81E−08	conditional	0.86	*PPM1M*	–/neurons/late prenatal
3	63792650	64004050	rs832187	2.58E−08	rs113386200	1.95E−12	primary	0.98	*THOC7*	–/–/–
3	135807405	136615405	rs7432375	5.27E−11	rs10935184	7.71E−25	primary	0.93	*PCCB*	–/–/–
4	170357552	170646052	rs10520163	1.02E−08	rs7438	1.02E−09	primary	0.97	*CLCN3*	–/–/–
5	45291475	46404116	rs1501357	1.24E−08	rs9292918	4.45E−05	primary	0.94	*BRCAT54, RP11-53O19.1*	–/–/adult
6	83779798	84407274	rs3798869	8.57E−10	rs2016358	1.19E−09	primary	0.90	*SNAP91*	cerebellar hemisphere/–/–
6	108875527	109019327	rs9398171	3.37E−08	rs111727905	3.84E−06	primary	0.97	*ZNF259P1*	–/–/early mid-prenatal
7	21485312	21545712	rs73060317	6.60E−07	rs141984481	3.59E−05	primary	0.92	*SP4*	–/–/early prenatal
8	8088038	10056127	rs2945232	2.03E−08	rs2980441	7.68E−69	primary	0.82	*FAM86B3P*	–/–/adolescent
8	26181524	26279124	rs1042992	2.27E−07	rs17055186	3.06E−24	conditional	0.91	*SDAD1P1*	testis/–/adult
8	38020424	38310924	rs57709857	2.32E−07	rs201999919	1.70E−07	primary	0.88	*WHSC1L1*	–/–/early prenatal
8	144822546	144871746	rs11784536	1.83E−07	rs12541792	6.45E−35	primary	0.90	*FAM83H*	esophagus - mucosa/ oligodendrocytes/adolescent

(*Continued on next page*)

Table 2. *Continued*

Chr	GWAS Locus Start	GWAS Locus End	GWAS Lead SNP	GWAS p Value	eSNP	eSNP p Value	Primary/ Conditional	PPH_4	Gene	Relevant Tissue/Cell Type/ Developmental Period
9	26839508	26909408	rs10967586	4.75E−07	rs12345197	3.90E−06	primary	0.80	*IFT74*	–/–/–
11	46340213	46751213	rs7951870	1.97E−11	rs16938506	5.08E−05	primary	0.88	*MDK*	–/–/early mid-prenatal
12	57428314	57497814	rs324017	2.13E−07	rs4559	2.02E−05	conditional	0.91	*STAT6*	–/microglia/adolescent
14	35421614	35847614	rs77477310	1.52E−07	rs1028449	8.09E−04	primary	0.84	*RP11-85K15.2*	–/–/–
15	78803032	78926732	rs8042374	1.87E−12	rs7171869	1.44E−04	conditional	0.94	*IREB2*	–/–/early prenatal
15	84661161	85153461	rs950169	7.62E−11	rs35677834	1.54E−34	primary	0.80	*LOC101929479, RP11-561C5.3*	ovary/–/early mid-prenatal
15	91416560	91436560	rs4702	2.30E−12	rs4702	4.49E−13	primary	1.00	*FURIN*	–/endothelial cells/adolescent
16	4447751	4596451	rs6500602	2.79E−07	rs3747580	4.75E−16	primary	0.90	*CORO7*	–/–/–
					rs8046295	2.68E−11	primary	0.89	*NMRAL1*	–/–/–
16	29924377	30144877	rs12691307	1.30E−10	rs4788203	1.95E−05	primary	0.88	*TMEM219*	–/–/–
					rs3935873	7.46E−14	primary	0.87	*INO80E*	–/neurons/–
					rs4787491	1.60E−04	conditional	0.82	*DOC2A*	brain - cortex/neurons/adolescent
16	58669293	58691393	rs12325245	1.15E−08	rs11647976	4.83E−04	primary	0.94	*CNOT1*	–/–/–
17	17722402	18030202	rs8082590	6.84E−09	rs4072739	4.74E−13	primary	0.92	*DRG2*	–/–/–
19	11839736	11859736	rs72986630	4.64E−08	rs72986630	2.20E−14	primary	1.00	*ZNF823*	–/endothelial cells/early prenatal
19	19374022	19658022	rs2905426	6.92E−09	rs2965199	9.22E−36	primary	0.87	*GATAD2A*	–/–/–
19	50067499	50135399	rs56873913	2.19E−07	rs5023763	9.32E−05	primary	0.93	*SNRNP70*	–/–/–
22	41408556	42689414	rs9607782	6.76E−12	rs200447424	1.87E−04	primary	0.96	*RANGAP1*	–/–/–

being present in brain tissue homogenate. We found that 60 stimulation-specific eQTL (FDR < 0.01 in interferon or lipopolysaccharide stimulated monocytes, but FDR ≥ 0.05 in naive monocytes) were also conditional eQTL in DLPFC. Notably, rs7171787, a conditional (tertiary) eQTL in our DLPFC analysis, is a stimulation-specific monocyte eQTL for the neurodevelopmental[56–58] gene *CYFIP1*. In our data, associations with specificity of expression across tissues, developmental periods, and cell types determined from single-cell RNA-sequencing data suggest that context specificity plays a role in the occurrence of multiple statistically independent eQTL. Cell type specificity is particularly strongly correlated with eQTL number, consistent with those cell types being present in the current tissue homogenate data. Since previous studies have shown the importance of developmental[59–62] or cell-specific contributions[61,63–66] to schizophrenia, interrogation of independent eQTL effects may elucidate developmental or tissue-specific effects obscured in whole-tissue eQTL studies.

This context specificity of expression regulation is potentially mediated through overlap of eSNPs with distal regulatory elements, such as enhancers. Conditional eQTL occur farther from transcription start sites than primary eQTL, consistent with effects on enhancers. In addition, while both primary and conditional eQTL are enriched in both active promoter and enhancer regions, their enrichment in active promoters diminishes with increasing conditional eQTL order. In other words, conditional eQTL show greater enrichment in enhancers relative to promoters than do primary eQTL.

Third, we have identified a number of candidate genes for which genetic variation for expression co-localizes with genetic variation for schizophrenia risk (Table 2), including cases of co-localization with conditional eQTL. Genetic co-localization is expected if gene expression causally mediates disease risk, although we recognize that co-localization could also result from pleiotropy or linkage, particularly in regions of extensive linkage disequilibrium and haplotype structure.[40,67] We also note that several co-localization methods have recently been developed,[37,38,40–42] and direct comparisons have found broad concordance among these methods and a high degree of specificity of positive results using coloc.[42,45,46] However, some differences in results would likely be achieved using alternative co-localization methods.

Our analyses prioritize 27 genes within 24 genome-wide significant (GWAS $p < 5 \times 10^{-8}$) SCZ loci and 19 genes in 17 suggestive ($p < 1 \times 10^{-6}$) loci. In addition to a number of previously implicated SCZ risk genes, our findings include several genes not previously considered as candidates,[35] in some cases—e.g., *SLC35E2*, *PTPRU* (MIM: 602454), *LINC01792*, *DCLK3*, *PPM1M*, *LOC101929479*—because the genes themselves do not overlap the GWAS locus regions but their eQTL do. In examining these genes for expression specificity in GTEx tissues, brain sample cell types from single-cell RNA-seq,[29] and in BrainSpan DLPFC developmental periods (Tables 2 and S8), we find their expression contexts show a diversity of patterns and can provide clues to generate specific hypotheses for functional follow-up of their potential roles in SCZ. Interestingly, genes broadly expressed across cell types tend to show prenatal expression.

Fourth, we highlight the importance of examining conditional eQTL for co-localization with GWASs. In at least 6 out of 40 loci showing GWAS-eQTL co-localization, a conditional eQTL signal co-localizes with SCZ risk. This is likely to be a conservative estimate, as the smaller effect sizes of conditional eQTL results in bias against detection of conditional GWAS-eQTL co-localization. If we had considered only primary eQTL in the analyses, these instances of co-localization would not have been identified. Among our highlighted conditional eQTL-GWAS co-localized genes are *IREB2*, *STAT6*, and *PROX1-AS1*. IREB2 (iron regulatory element binding protein 2) is a key regulator of iron homeostasis[68,69] that has been previously implicated in neurodegenerative disorders.[70,71] Mouse *IREB2* homolog *Irp2* knockouts exhibit impairments in coordination and balance, exploration, and nociception.[69] The immune-related transcription factor STAT6 induces interleukin 4 (IL-4)-mediated anti-apoptotic activity of T helper cells, and the locus is associated with migraine[72,73] and brain glioma[74] as well as several immune/inflammatory diseases.[75–77] STAT6 also activates neuronal progenitor/stem cells and neurogenesis,[78] making it intriguing as an immune-related SCZ candidate given recent observations about the role of complement factor 4 (*C4*) gene as a SCZ risk gene[79] and prior work potentially implicating microglia.[80] Consistent with a role in immune-mediated synaptic pruning, *STAT6* expression is broadly postnatal and shows specificity for microglia (Table S8). *PROX1-AS1* encodes a lncRNA that has been implicated as aberrantly expressed in several cancers, is upregulated in the cell cycle S-phase, and promotes G1/S transition in cell culture.[81] As a potential regulator of the Prospero Homeobox 1 (PROX1) transcription factor, it could be involved in development and cell differentiation in several tissues, including oligodendrocytes[82] and GABAergic interneurons[83] in the brain. *PROX1-AS1* expression is specific to neurons and mature oligodendrocytes and is expressed postnatally (Table S8).

In conclusion, we find that conditional eQTL are widespread and are consistent with complex and context-specific regulation. Accounting for conditional eQTL leads to new findings of GWAS-eQTL co-localization and generates specific hypotheses for the role of gene expression regulation in disease etiology. The analytical strategy presented here could be implemented as a means of identification of putatively causal genes for any phenotype in which GWAS summary statistics and expression and genotype data from the GWAS phenotype-relevant tissue are available. Conditional eQTL that co-localize with disease risk may reflect regulatory mechanisms that are important in a key developmental period or individual cell type and may be missed when focusing on primary eQTL discovered

in adult whole tissue. As further efforts are made to generate data across ranges of tissues or individual cell types, we may have a better ability to directly identify regulatory variants specific to these contexts. However, if a variant is primarily active in a very specific time point or stimulus condition, capturing data reflecting this condition will remain challenging. Conditional co-localization analysis in well-powered eQTL cohorts may best identify the genes driving these trait associations, though further validation work will be required to understand the mechanism by which the gene contributes to disease risk.

Supplemental Data

Supplemental Data include 18 figures and 12 tables and can be found with this article online at https://doi.org/10.1016/j.ajhg.2018.04.011.

Consortia

CMC leadership: Pamela Sklar, Joseph Buxbaum (Icahn School of Medicine at Mount Sinai), Bernie Devlin, David Lewis (University of Pittsburgh), Raquel Gur, Chang-Gyu Hahn (University of Pennsylvania), Keisuke Hirai, Hiroyoshi Toyoshiba (Takeda Pharmaceuticals Company Limited), Enrico Domenici, Laurent Essioux (F. Hoffmann-La Roche Ltd), Lara Mangravite, Mette Peters (Sage Bionetworks), Thomas Lehner, and Barbara Lipska (NIMH). Additional members of CMC: A. Ercument Cicek, Cong Lu, Kathryn Roeder, Lu Xie (Carnegie Mellon Univ.); Konrad Talbot (Cedars-Sinai Medical Center); Scott E. Hemby (High Point Univ.); Laurent Essioux (Hoffmann-La Roche); Andrew Browne, Andrew Chess, Aaron Topol, Alexander Charney, Amanda Dobbyn, Ben Readhead, Bin Zhang, Dalila Pinto, David A. Bennett, David H. Kavanagh, Douglas M. Ruderfer, Eli A. Stahl, Eric E. Schadt, Gabriel E. Hoffman, Hardik R. Shah, Jun Zhu, Jessica S. Johnson, John F. Fullard, Joel T. Dudley, Kiran Girdhar, Kristen J. Brennand, Laura G. Sloofman, Laura M. Huckins, Menachem Fromer, Milind C. Mahajan, Panos Roussos, Schahram Akbarian, Shaun M. Purcell, Tymor Hamamsy, Towfique Raj, Vahram Haroutunian, Ying-Chih Wang, Zeynep H. Gümüş (Mount Sinai School of Med.); Geetha Senthil, Robin Kramer (NIMH); Benjamin A. Logsdon, Jonathan M.J. Derry, Kristen K. Dang, Solveig K. Sieberts, Thanneer M. Perumal (Sage Bionetworks); Roberto Visintainer (Univ. Trento, Italy); Leslie A. Shinobu (Takeda); Patrick F. Sullivan (Univ. North Carolina); and Lambertus L. Klei (Univ. Pittsburgh School of Med.).

Acknowledgments

Dedicated to the memory of Pamela Sklar, MD, PhD.

Data were generated as part of the CommonMind Consortium supported by funding from Takeda Pharmaceuticals Company Limited, F. Hoffmann-La Roche Ltd and NIH grants R01MH085542, R01MH093725, P50MH066392, P50MH080405, R01MH097276, RO1-MH-075916, P50M096891, P50MH084053S1, R37MH057881, R37MH057881S1, HHSN271201300031C, AG02219, AG05138, and MH06692. Brain tissue for the study was obtained from the following brain bank collections: the Mount Sinai NIH Brain and Tissue Repository, the University of Pennsylvania Alzheimer Disease Core Center, and the University of Pittsburgh NeuroBioBank and Brain and Tissue Repositories. Data from the NIMH Human Brain Collection Core were generated as part of the NIMH Human Brain Collection Core (NIH NCT00001260, 999917073).

ROSMAP study data were provided by the Rush Alzheimer Disease Center, Rush University Medical Center, Chicago. Data collection was supported through funding by NIA grants P30AG10161, R01AG15819, R01AG17917, R01AG30146, R01AG36836, U01AG32984, U01AG46152, the Illinois Department of Public Health, and the Translational Genomics Research Institute. The Genotype-Tissue Expression (GTEx) Project was supported by the Common Fund of the Office of the Director of the National Institutes of Health, and by NCI, NHGRI, NHLBI, NIDA, NIMH, and NINDS. The data used for the analyses described in this manuscript were obtained from the GTEx Portal on 09/05/16. BrainSpan: Atlas of the Developing Human Brain. Funded by ARRA Awards 1RC2MH089921-01, 1RC2MH090047-01, and 1RC2MH089929-01.

Received: April 6, 2017
Accepted: April 24, 2018
Published: May 24, 2018

Web Resources

AMP-AD Knowledge Portal, https://www.synapse.org/ampad

BrainSpan – Atlas of the Developing Human Brain, http://www.brainspan.org/

CommonMind Consortium data, https://www.synapse.org/CMC

CommonMind Consortium ChIP-seq data, https://www.synapse.org/#!Synapse:syn8040458

coloc2, https://github.com/Stahl-Lab-MSSM/coloc2

dbGaP (accession number phs000979), http://ncbi.nlm.nih.gov/gap

ExAC Functional Gene Constraint, http://exac.broadinstitute.org/downloads

GCTA, http://cnsgenomics.com/software/gcta/

GemTools, http://wpicr.wpic.pitt.edu/WPICCompgen/GemTools/GemTools.htm

GEO (accession number GSE67835), https://www.ncbi.nlm.nih.gov/geo/

GTEx Portal, https://www.gtexportal.org/home/

HBCC microarray cohort, dbGaP (ID: phs000979.v1.p1), https://www.ncbi.nlm.nih.gov/gap

LDetect LD blocks, https://bitbucket.org/nygcresearch/ldetect-data/overview

NIH Roadmap Epigenomics Project chromatin state learning, http://egg2.wustl.edu/roadmap/web_portal/chr_state_learning.html#core_15state

OMIM, http://www.omim.org/

qvalue, http://bioconductor.org/packages/release/bioc/html/qvalue.html

R statistical software, https://www.r-project.org/

SNPsnap, https://data.broadinstitute.org/mpg/snpsnap/

SVA: Surrogate Variable Analysis, R package version 3.24.4, http://bioconductor.org/packages/release/bioc/html/sva.html

variancePartition, http://bioconductor.org/packages/release/bioc/html/variancePartition.html

References

1. Gilad, Y., Rifkin, S.A., and Pritchard, J.K. (2008). Revealing the architecture of gene regulation: the promise of eQTL studies. Trends Genet. *24*, 408–415.

2. Cookson, W., Liang, L., Abecasis, G., Moffatt, M., and Lathrop, M. (2009). Mapping complex disease traits with global gene expression. Nat. Rev. Genet. *10*, 184–194.
3. Montgomery, S.B., and Dermitzakis, E.T. (2011). From expression QTLs to personalized transcriptomics. Nat. Rev. Genet. *12*, 277–282.
4. Albert, F.W., and Kruglyak, L. (2015). The role of regulatory variation in complex traits and disease. Nat. Rev. Genet. *16*, 197–212.
5. Moffatt, M.F., Kabesch, M., Liang, L., Dixon, A.L., Strachan, D., Heath, S., Depner, M., von Berg, A., Bufe, A., Rietschel, E., et al. (2007). Genetic variants regulating ORMDL3 expression contribute to the risk of childhood asthma. Nature *448*, 470–473.
6. Speliotes, E.K., Willer, C.J., Berndt, S.I., Monda, K.L., Thorleifsson, G., Jackson, A.U., Lango Allen, H., Lindgren, C.M., Luan, J., Mägi, R., et al.; MAGIC; and Procardis Consortium (2010). Association analyses of 249,796 individuals reveal 18 new loci associated with body mass index. Nat. Genet. *42*, 937–948.
7. Dubois, P.C., Trynka, G., Franke, L., Hunt, K.A., Romanos, J., Curtotti, A., Zhernakova, A., Heap, G.A., Adány, R., Aromaa, A., et al. (2010). Multiple common variants for celiac disease influencing immune gene expression. Nat. Genet. *42*, 295–302.
8. Libioulle, C., Louis, E., Hansoul, S., Sandor, C., Farnir, F., Franchimont, D., Vermeire, S., Dewit, O., de Vos, M., Dixon, A., et al. (2007). Novel Crohn disease locus identified by genome-wide association maps to a gene desert on 5p13.1 and modulates expression of PTGER4. PLoS Genet. *3*, e58.
9. Lappalainen, T., Sammeth, M., Friedländer, M.R., 't Hoen, P.A., Monlong, J., Rivas, M.A., Gonzàlez-Porta, M., Kurbatova, N., Griebel, T., Ferreira, P.G., et al.; Geuvadis Consortium (2013). Transcriptome and genome sequencing uncovers functional variation in humans. Nature *501*, 506–511.
10. Battle, A., Brown, C.D., Engelhardt, B.E., Montgomery, S.B.; GTEx Consortium; Laboratory, Data Analysis &Coordinating Center (LDACC)—Analysis Working Group; Statistical Methods groups—Analysis Working Group; Enhancing GTEx (eGTEx) groups; NIH Common Fund; NIH/NCI; NIH/NHGRI; NIH/NIMH; NIH/NIDA; Biospecimen Collection Source Site—NDRI; Biospecimen Collection Source Site—RPCI; Biospecimen Core Resource—VARI; Brain Bank Repository—University of Miami Brain Endowment Bank; Leidos Biomedical—Project Management; ELSI Study; Genome Browser Data Integration &Visualization—EBI; Genome Browser Data Integration &Visualization—UCSC Genomics Institute, University of California Santa Cruz; Lead analysts; Laboratory, Data Analysis &Coordinating Center (LDACC); NIH program management; Biospecimen collection; Pathology; and eQTL manuscript working group (2017). Genetic effects on gene expression across human tissues. Nature *550*, 204–213.
11. Jansen, R., Hottenga, J.J., Nivard, M.G., Abdellaoui, A., Laport, B., de Geus, E.J., Wright, F.A., Penninx, B.W.J.H., and Boomsma, D.I. (2017). Conditional eQTL analysis reveals allelic heterogeneity of gene expression. Hum. Mol. Genet. *26*, 1444–1451.
12. Zhernakova, D.V., Deelen, P., Vermaat, M., van Iterson, M., van Galen, M., Arindrarto, W., van 't Hof, P., Mei, H., van Dijk, F., Westra, H.J., et al. (2017). Identification of context-dependent expression quantitative trait loci in whole blood. Nat. Genet. *49*, 139–145.
13. Dimas, A.S., Deutsch, S., Stranger, B.E., Montgomery, S.B., Borel, C., Attar-Cohen, H., Ingle, C., Beazley, C., Gutierrez Arcelus, M., Sekowska, M., et al. (2009). Common regulatory variation impacts gene expression in a cell type-dependent manner. Science *325*, 1246–1250.
14. Liu, X., Finucane, H.K., Gusev, A., Bhatia, G., Gazal, S., O'Connor, L., Bulik-Sullivan, B., Wright, F.A., Sullivan, P.F., Neale, B.M., and Price, A.L. (2017). Functional architectures of local and distal regulation of gene expression in multiple human tissues. Am. J. Hum. Genet. *100*, 605–616.
15. Soldner, F., Stelzer, Y., Shivalila, C.S., Abraham, B.J., Latourelle, J.C., Barrasa, M.I., Goldmann, J., Myers, R.H., Young, R.A., and Jaenisch, R. (2016). Parkinson-associated risk variant in distal enhancer of α-synuclein modulates target gene expression. Nature *533*, 95–99.
16. Fromer, M., Roussos, P., Sieberts, S.K., Johnson, J.S., Kavanagh, D.H., Perumal, T.M., Ruderfer, D.M., Oh, E.C., Topol, A., Shah, H.R., et al. (2016). Gene expression elucidates functional impact of polygenic risk for schizophrenia. Nat. Neurosci. *19*, 1442–1453.
17. O'Connell, J., Gurdasani, D., Delaneau, O., Pirastu, N., Ulivi, S., Cocca, M., Traglia, M., Huang, J., Huffman, J.E., Rudan, I., et al. (2014). A general approach for haplotype phasing across the full spectrum of relatedness. PLoS Genet. *10*, e1004234.
18. Howie, B., Fuchsberger, C., Stephens, M., Marchini, J., and Abecasis, G.R. (2012). Fast and accurate genotype imputation in genome-wide association studies through pre-phasing. Nat. Genet. *44*, 955–959.
19. Abecasis, G.R., Auton, A., Brooks, L.D., DePristo, M.A., Durbin, R.M., Handsaker, R.E., Kang, H.M., Marth, G.T., McVean, G.A.; and 1000 Genomes Project Consortium (2012). An integrated map of genetic variation from 1,092 human genomes. Nature *491*, 56–65.
20. Leek, J.T., Johnson, W.E., Parker, H.S., Jaffe, A.E., and Storey, J.D. (2012). The sva package for removing batch effects and other unwanted variation in high-throughput experiments. Bioinformatics *28*, 882–883.
21. Shabalin, A.A. (2012). Matrix eQTL: ultra fast eQTL analysis via large matrix operations. Bioinformatics *28*, 1353–1358.
22. De Jager, P.L., Srivastava, G., Lunnon, K., Burgess, J., Schalkwyk, L.C., Yu, L., Eaton, M.L., Keenan, B.T., Ernst, J., McCabe, C., et al. (2014). Alzheimer's disease: early alterations in brain DNA methylation at ANK1, BIN1, RHBDF2 and other loci. Nat. Neurosci. *17*, 1156–1163.
23. McCarthy, S., Das, S., Kretzschmar, W., Delaneau, O., Wood, A.R., Teumer, A., Kang, H.M., Fuchsberger, C., Danecek, P., Sharp, K., et al.; Haplotype Reference Consortium (2016). A reference panel of 64,976 haplotypes for genotype imputation. Nat. Genet. *48*, 1279–1283.
24. Das, S., Forer, L., Schönherr, S., Sidore, C., Locke, A.E., Kwong, A., Vrieze, S.I., Chew, E.Y., Levy, S., McGue, M., et al. (2016). Next-generation genotype imputation service and methods. Nat. Genet. *48*, 1284–1287.
25. Yang, J., Lee, S.H., Goddard, M.E., and Visscher, P.M. (2011). GCTA: a tool for genome-wide complex trait analysis. Am. J. Hum. Genet. *88*, 76–82.
26. Yanai, I., Benjamin, H., Shmoish, M., Chalifa-Caspi, V., Shklar, M., Ophir, R., Bar-Even, A., Horn-Saban, S., Safran, M., Domany, E., et al. (2005). Genome-wide midrange transcription

profiles reveal expression level relationships in human tissue specification. Bioinformatics *21*, 650–659.
27. Kryuchkova-Mostacci, N., and Robinson-Rechavi, M. (2017). A benchmark of gene expression tissue-specificity metrics. Brief. Bioinform. *18*, 205–214.
28. GTEx Consortium (2013). The Genotype-Tissue Expression (GTEx) project. Nat. Genet. *45*, 580–585.
29. Darmanis, S., Sloan, S.A., Zhang, Y., Enge, M., Caneda, C., Shuer, L.M., Hayden Gephart, M.G., Barres, B.A., and Quake, S.R. (2015). A survey of human brain transcriptome diversity at the single cell level. Proc. Natl. Acad. Sci. USA *112*, 7285–7290.
30. Lin, G.N., Corominas, R., Lemmens, I., Yang, X., Tavernier, J., Hill, D.E., Vidal, M., Sebat, J., and Iakoucheva, L.M. (2015). Spatiotemporal 16p11.2 protein network implicates cortical late mid-fetal brain development and KCTD13-Cul3-RhoA pathway in psychiatric diseases. Neuron *85*, 742–754.
31. Pers, T.H., Timshel, P., and Hirschhorn, J.N. (2015). SNPsnap: a Web-based tool for identification and annotation of matched SNPs. Bioinformatics *31*, 418–420.
32. Kundaje, A., Meuleman, W., Ernst, J., Bilenky, M., Yen, A., Heravi-Moussavi, A., Kheradpour, P., Zhang, Z., Wang, J., Ziller, M.J., et al.; Roadmap Epigenomics Consortium (2015). Integrative analysis of 111 reference human epigenomes. Nature *518*, 317–330.
33. Ernst, J., and Kellis, M. (2012). ChromHMM: automating chromatin-state discovery and characterization. Nat. Methods *9*, 215–216.
34. Hoffman, G.E., and Schadt, E.E. (2016). variancePartition: interpreting drivers of variation in complex gene expression studies. BMC Bioinformatics *17*, 483.
35. Schizophrenia Working Group of the Psychiatric Genomics Consortium (2014). Biological insights from 108 schizophrenia-associated genetic loci. Nature *511*, 421–427.
36. Pruim, R.J., Welch, R.P., Sanna, S., Teslovich, T.M., Chines, P.S., Gliedt, T.P., Boehnke, M., Abecasis, G.R., and Willer, C.J. (2010). LocusZoom: regional visualization of genome-wide association scan results. Bioinformatics *26*, 2336–2337.
37. Nica, A.C., Montgomery, S.B., Dimas, A.S., Stranger, B.E., Beazley, C., Barroso, I., and Dermitzakis, E.T. (2010). Candidate causal regulatory effects by integration of expression QTLs with complex trait genetic associations. PLoS Genet. *6*, e1000895.
38. He, X., Fuller, C.K., Song, Y., Meng, Q., Zhang, B., Yang, X., and Li, H. (2013). Sherlock: detecting gene-disease associations by matching patterns of expression QTL and GWAS. Am. J. Hum. Genet. *92*, 667–680.
39. Giambartolomei, C., Vukcevic, D., Schadt, E.E., Franke, L., Hingorani, A.D., Wallace, C., and Plagnol, V. (2014). Bayesian test for colocalisation between pairs of genetic association studies using summary statistics. PLoS Genet. *10*, e1004383.
40. Zhu, Z., Zhang, F., Hu, H., Bakshi, A., Robinson, M.R., Powell, J.E., Montgomery, G.W., Goddard, M.E., Wray, N.R., Visscher, P.M., and Yang, J. (2016). Integration of summary data from GWAS and eQTL studies predicts complex trait gene targets. Nat. Genet. *48*, 481–487.
41. Hormozdiari, F., van de Bunt, M., Segrè, A.V., Li, X., Joo, J.W.J., Bilow, M., Sul, J.H., Sankararaman, S., Pasaniuc, B., and Eskin, E. (2016). Colocalization of GWAS and eQTL Signals Detects Target Genes. Am. J. Hum. Genet. *99*, 1245–1260.
42. Chun, S., Casparino, A., Patsopoulos, N.A., Croteau-Chonka, D.C., Raby, B.A., De Jager, P.L., Sunyaev, S.R., and Cotsapas, C. (2017). Limited statistical evidence for shared genetic effects of eQTLs and autoimmune-disease-associated loci in three major immune-cell types. Nat. Genet. *49*, 600–605.
43. Pickrell, J.K., Berisa, T., Liu, J.Z., Ségurel, L., Tung, J.Y., and Hinds, D.A. (2016). Detection and interpretation of shared genetic influences on 42 human traits. Nat. Genet. *48*, 709–717.
44. Mancuso, N., Shi, H., Goddard, P., Kichaev, G., Gusev, A., and Pasaniuc, B. (2017). Integrating gene expression with summary association statistics to identify genes associated with 30 complex traits. Am. J. Hum. Genet. *100*, 473–487.
45. Barbeira, A.N., Dickinson, S.P., Torres, J.M., Bonazzola, R., Zheng, J., Torstenson, E.S., Wheeler, H.E., Shah, K.P., Edwards, T., Garcia, T., et al. (2018). Exploring the phenotypic consequences of tissue specific gene expression variation inferred from GWAS summary statistics. Nat. Comm. *9*, 1825.
46. Ongen, H., Brown, A.A., Delaneau, O., Panousis, N.I., Nica, A.C., Dermitzakis, E.T.; and GTEx Consortium (2017). Estimating the causal tissues for complex traits and diseases. Nat. Genet. *49*, 1676–1683.
47. Wakefield, J. (2009). Bayes factors for genome-wide association studies: comparison with P-values. Genet. Epidemiol. *33*, 79–86.
48. Berisa, T., and Pickrell, J.K. (2016). Approximately independent linkage disequilibrium blocks in human populations. Bioinformatics *32*, 283–285.
49. Lek, M., Karczewski, K.J., Minikel, E.V., Samocha, K.E., Banks, E., Fennell, T., O'Donnell-Luria, A.H., Ware, J.S., Hill, A.J., Cummings, B.B., et al.; Exome Aggregation Consortium (2016). Analysis of protein-coding genetic variation in 60,706 humans. Nature *536*, 285–291.
50. Eisenberg, E., and Levanon, E.Y. (2013). Human housekeeping genes, revisited. Trends Genet. *29*, 569–574.
51. Farh, K.K., Marson, A., Zhu, J., Kleinewietfeld, M., Housley, W.J., Beik, S., Shoresh, N., Whitton, H., Ryan, R.J., Shishkin, A.A., et al. (2015). Genetic and epigenetic fine mapping of causal autoimmune disease variants. Nature *518*, 337–343.
52. Tak, Y.G., and Farnham, P.J. (2015). Making sense of GWAS: using epigenomics and genome engineering to understand the functional relevance of SNPs in non-coding regions of the human genome. Epigenetics Chromatin *8*, 57.
53. Brown, C.D., Mangravite, L.M., and Engelhardt, B.E. (2013). Integrative modeling of eQTLs and cis-regulatory elements suggests mechanisms underlying cell type specificity of eQTLs. PLoS Genet. *9*, e1003649.
54. Gaffney, D.J., Veyrieras, J.B., Degner, J.F., Pique-Regi, R., Pai, A.A., Crawford, G.E., Stephens, M., Gilad, Y., and Pritchard, J.K. (2012). Dissecting the regulatory architecture of gene expression QTLs. Genome Biol. *13*, R7.
55. Fairfax, B.P., Humburg, P., Makino, S., Naranbhai, V., Wong, D., Lau, E., Jostins, L., Plant, K., Andrews, R., McGee, C., and Knight, J.C. (2014). Innate immune activity conditions the effect of regulatory variants upon monocyte gene expression. Science *343*, 1246949.
56. Stefansson, H., Rujescu, D., Cichon, S., Pietiläinen, O.P., Ingason, A., Steinberg, S., Fossdal, R., Sigurdsson, E., Sigmundsson, T., Buizer-Voskamp, J.E., et al.; GROUP (2008). Large recurrent microdeletions associated with schizophrenia. Nature *455*, 232–236.
57. van der Zwaag, B., Staal, W.G., Hochstenbach, R., Poot, M., Spierenburg, H.A., de Jonge, M.V., Verbeek, N.E., van 't Slot, R., van Es, M.A., Staal, F.J., et al. (2010). A co-segregating microduplication of chromosome 15q11.2 pinpoints two risk

genes for autism spectrum disorder. Am. J. Med. Genet. B. Neuropsychiatr. Genet. *153B*, 960–966.

58. Pathania, M., Davenport, E.C., Muir, J., Sheehan, D.F., López-Doménech, G., and Kittler, J.T. (2014). The autism and schizophrenia associated gene CYFIP1 is critical for the maintenance of dendritic complexity and the stabilization of mature spines. Transl. Psychiatry *4*, e374.
59. Birnbaum, R., Jaffe, A.E., Chen, Q., Hyde, T.M., Kleinman, J.E., and Weinberger, D.R. (2015). Investigation of the prenatal expression patterns of 108 schizophrenia-associated genetic loci. Biol. Psychiatry *77*, e43–e51.
60. Jaffe, A.E., Gao, Y., Deep-Soboslay, A., Tao, R., Hyde, T.M., Weinberger, D.R., and Kleinman, J.E. (2016). Mapping DNA methylation across development, genotype and schizophrenia in the human frontal cortex. Nat. Neurosci. *19*, 40–47.
61. Marín, O. (2016). Developmental timing and critical windows for the treatment of psychiatric disorders. Nat. Med. *22*, 1229–1238.
62. Gulsuner, S., Walsh, T., Watts, A.C., Lee, M.K., Thornton, A.M., Casadei, S., Rippey, C., Shahin, H., Nimgaonkar, V.L., Go, R.C., et al.; Consortium on the Genetics of Schizophrenia (COGS); and PAARTNERS Study Group (2013). Spatial and temporal mapping of de novo mutations in schizophrenia to a fetal prefrontal cortical network. Cell *154*, 518–529.
63. Windrem, M.S., Osipovitch, M., Liu, Z., Bates, J., Chandler-Militello, D., Zou, L., Munir, J., Schanz, S., McCoy, K., Miller, R.H., et al. (2017). Human iPSC glial mouse chimeras reveal glial contributions to schizophrenia. Cell Stem Cell *21*, 195–208.e6.
64. Skene, N.G., Bryois, J., Bakken, T.E., Breen, G., Crowley, J.J., Gaspar, H., Giusti-Rodriguez, P., Hodge, R.D., Miller, J.A., Munoz-Manchado, A., et al. (2017). Genetic identification of brain cell types underlying schizophrenia. bioRxiv. https://doi.org/10.1101/145466.
65. Wang, L., Lockstone, H.E., Guest, P.C., Levin, Y., Palotás, A., Pietsch, S., Schwarz, E., Rahmoune, H., Harris, L.W., Ma, D., and Bahn, S. (2010). Expression profiling of fibroblasts identifies cell cycle abnormalities in schizophrenia. J. Proteome Res. *9*, 521–527.
66. Vitale, A.M., Matigian, N.A., Cristino, A.S., Nones, K., Ravishankar, S., Bellette, B., Fan, Y., Wood, S.A., Wolvetang, E., and Mackay-Sim, A. (2017). DNA methylation in schizophrenia in different patient-derived cell types. NPJ Schizophr *3*, 6.
67. Guo, H., Fortune, M.D., Burren, O.S., Schofield, E., Todd, J.A., and Wallace, C. (2015). Integration of disease association and eQTL data using a Bayesian colocalisation approach highlights six candidate causal genes in immune-mediated diseases. Hum. Mol. Genet. *24*, 3305–3313.
68. Kim, W.J., and Lee, S.D. (2015). Candidate genes for COPD: current evidence and research. Int. J. Chron. Obstruct. Pulmon. Dis. *10*, 2249–2255.
69. Zumbrennen-Bullough, K.B., Becker, L., Garrett, L., Hölter, S.M., Calzada-Wack, J., Mossbrugger, I., Quintanilla-Fend, L., Racz, I., Rathkolb, B., Klopstock, T., et al. (2014). Abnormal brain iron metabolism in Irp2 deficient mice is associated with mild neurological and behavioral impairments. PLoS ONE *9*, e98072.
70. Dusek, P., Jankovic, J., and Le, W. (2012). Iron dysregulation in movement disorders. Neurobiol. Dis. *46*, 1–18.
71. Rouault, T.A. (2013). Iron metabolism in the CNS: implications for neurodegenerative diseases. Nat. Rev. Neurosci. *14*, 551–564.
72. Anttila, V., Winsvold, B.S., Gormley, P., Kurth, T., Bettella, F., McMahon, G., Kallela, M., Malik, R., de Vries, B., Terwindt, G., et al.; North American Brain Expression Consortium; and UK Brain Expression Consortium (2013). Genome-wide meta-analysis identifies new susceptibility loci for migraine. Nat. Genet. *45*, 912–917.
73. Gormley, P., Anttila, V., Winsvold, B.S., Palta, P., Esko, T., Pers, T.H., Farh, K.H., Cuenca-Leon, E., Muona, M., Furlotte, N.A., et al.; International Headache Genetics Consortium (2016). Meta-analysis of 375,000 individuals identifies 38 susceptibility loci for migraine. Nat. Genet. *48*, 856–866.
74. Ruan, Z., Zhao, Y., Yan, L., Chen, H., Fan, W., Chen, J., Wu, Q., Qian, J., Zhang, T., Zhou, K., et al. (2011). Single nucleotide polymorphisms in IL-4Ra, IL-13 and STAT6 genes occurs in brain glioma. Front. Biosci. (Elite Ed.) *3*, 33–45.
75. Sleiman, P.M., Wang, M.L., Cianferoni, A., Aceves, S., Gonsalves, N., Nadeau, K., Bredenoord, A.J., Furuta, G.T., Spergel, J.M., and Hakonarson, H. (2014). GWAS identifies four novel eosinophilic esophagitis loci. Nat. Commun. *5*, 5593.
76. Granada, M., Wilk, J.B., Tuzova, M., Strachan, D.P., Weidinger, S., Albrecht, E., Gieger, C., Heinrich, J., Himes, B.E., Hunninghake, G.M., et al. (2012). A genome-wide association study of plasma total IgE concentrations in the Framingham Heart Study. J. Allergy Clin. Immunol. *129*, 840–845.e21.
77. Bønnelykke, K., Matheson, M.C., Pers, T.H., Granell, R., Strachan, D.P., Alves, A.C., Linneberg, A., Curtin, J.A., Warrington, N.M., Standl, M., et al.; AAGC (2013). Meta-analysis of genome-wide association studies identifies ten loci influencing allergic sensitization. Nat. Genet. *45*, 902–906.
78. Bhattarai, P., Thomas, A.K., Cosacak, M.I., Papadimitriou, C., Mashkaryan, V., Froc, C., Reinhardt, S., Kurth, T., Dahl, A., Zhang, Y., and Kizil, C. (2016). IL4/STAT6 signaling activates neural stem cell proliferation and neurogenesis upon amyloid-β42 aggregation in adult zebrafish brain. Cell Rep. *17*, 941–948.
79. Sekar, A., Bialas, A.R., de Rivera, H., Davis, A., Hammond, T.R., Kamitaki, N., Tooley, K., Presumey, J., Baum, M., Van Doren, V., et al.; Schizophrenia Working Group of the Psychiatric Genomics Consortium (2016). Schizophrenia risk from complex variation of complement component 4. Nature *530*, 177–183.
80. Schafer, D.P., Lehrman, E.K., Kautzman, A.G., Koyama, R., Mardinly, A.R., Yamasaki, R., Ransohoff, R.M., Greenberg, M.E., Barres, B.A., and Stevens, B. (2012). Microglia sculpt postnatal neural circuits in an activity and complement-dependent manner. Neuron *74*, 691–705.
81. Yang, F., Yi, F., Zheng, Z., Ling, Z., Ding, J., Guo, J., Mao, W., Wang, X., Wang, X., Ding, X., et al. (2012). Characterization of a carcinogenesis-associated long non-coding RNA. RNA Biol. *9*, 110–116.
82. Kato, K., Konno, D., Berry, M., Matsuzaki, F., Logan, A., and Hidalgo, A. (2015). Prox1 inhibits proliferation and is required for differentiation of the oligodendrocyte cell lineage in the mouse. PLoS ONE *10*, e0145334.
83. Miyoshi, G., Young, A., Petros, T., Karayannis, T., McKenzie Chang, M., Lavado, A., Iwano, T., Nakajima, M., Taniguchi, H., Huang, Z.J., et al. (2015). Prox1 regulates the subtype-specific development of caudal ganglionic eminence-derived GABAergic cortical interneurons. J. Neurosci. *35*, 12869–12889.

The American Journal of Human Genetics, Volume *102*

Supplemental Data

Landscape of Conditional eQTL

in Dorsolateral Prefrontal Cortex

and Co-localization with Schizophrenia GWAS

Amanda Dobbyn, Laura M. Huckins, James Boocock, Laura G. Sloofman, Benjamin S. Glicksberg, Claudia Giambartolomei, Gabriel E. Hoffman, Thanneer M. Perumal, Kiran Girdhar, Yan Jiang, Towfique Raj, Douglas M. Ruderfer, Robin S. Kramer, Dalila Pinto, the CommonMind Consortium, Schahram Akbarian, Panos Roussos, Enrico Domenici, Bernie Devlin, Pamela Sklar, Eli A. Stahl, and Solveig K. Sieberts

SUPPLEMENTAL FIGURES

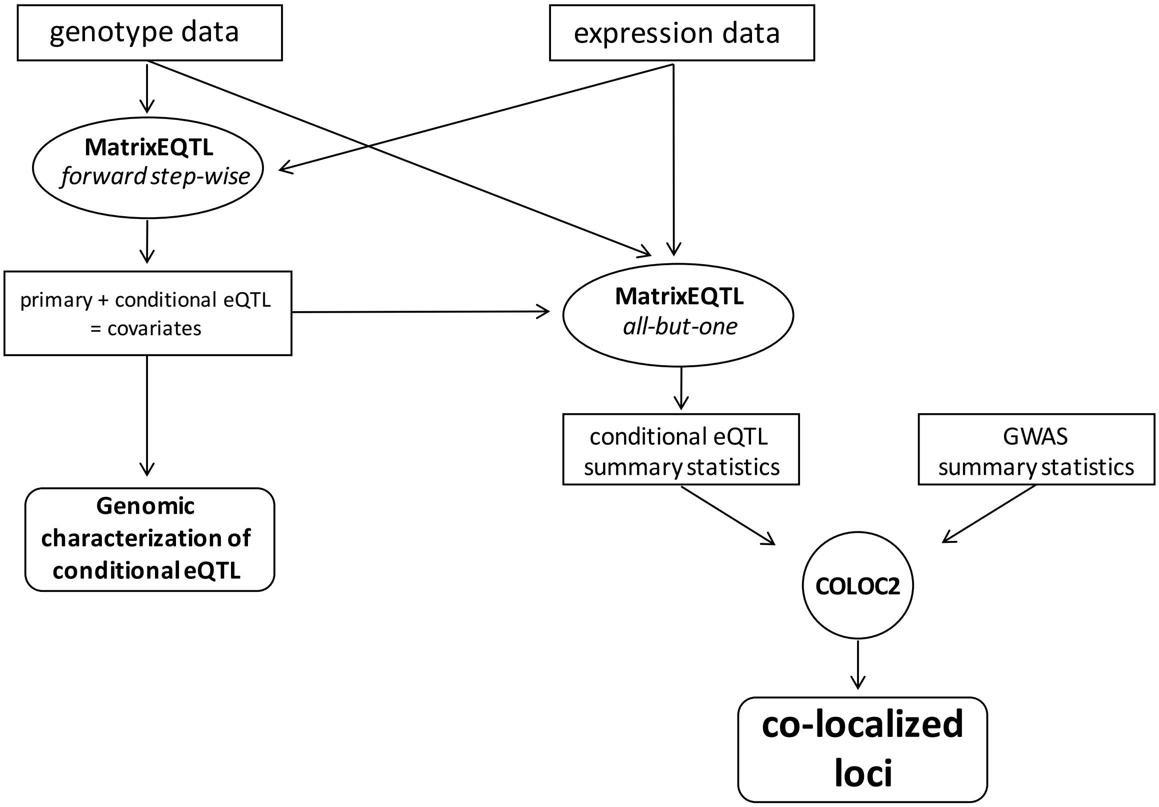

Figure S1. Overview of Workflow

Genotype and DLPFC expression data from the CommonMind Consortium are used to identify primary and conditional eQTL, implemented in MatrixEQTL. The resulting conditionally independent eQTL are then used as covariates, along with the genotype and expression data, to isolate each independent eQTL signature in an “all-but-one” analysis. These independent eQTL summary statistics and the 2014 PGC schizophrenia GWAS summary statistics are used as input to COLOC2, in order to test for co-localization between eQTL and GWAS signatures.

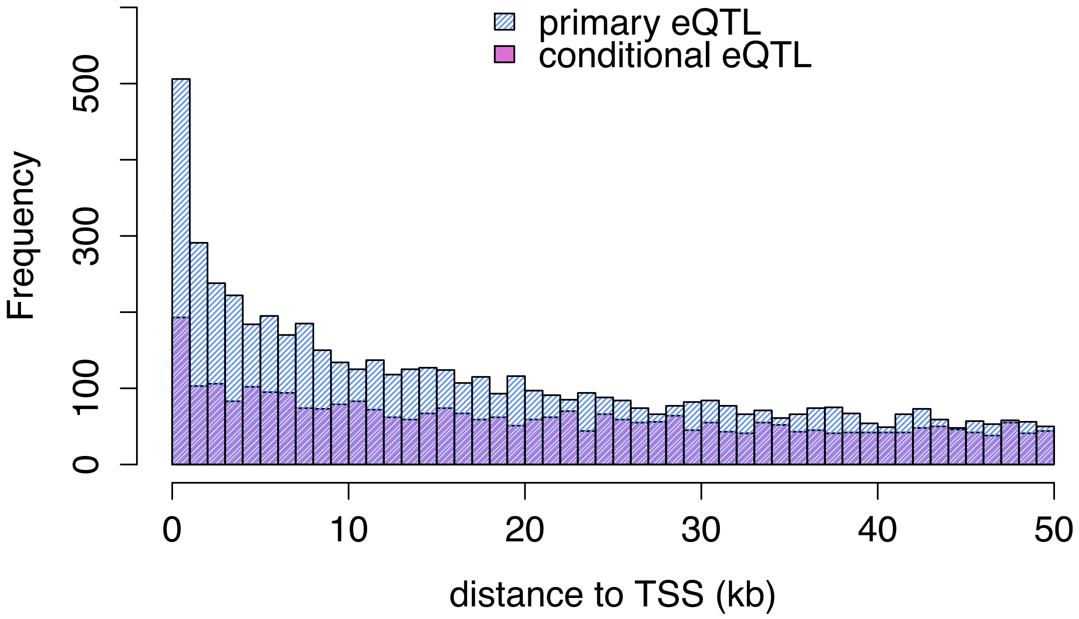

Figure S2. Distance from eQTL to transcription start site (TSS)

An overlapping histogram showing the numbers of eQTL (y-axis) occurring at increasing distances to TSS (x-axis), focusing on the 50 Kb window around the TSS. Even when examining smaller distances to the TSS, there is a difference between primary (blue) and conditional (pink) eQTL distance to TSS, with primary eQTL generally found closer to the TSS than conditional eQTL.

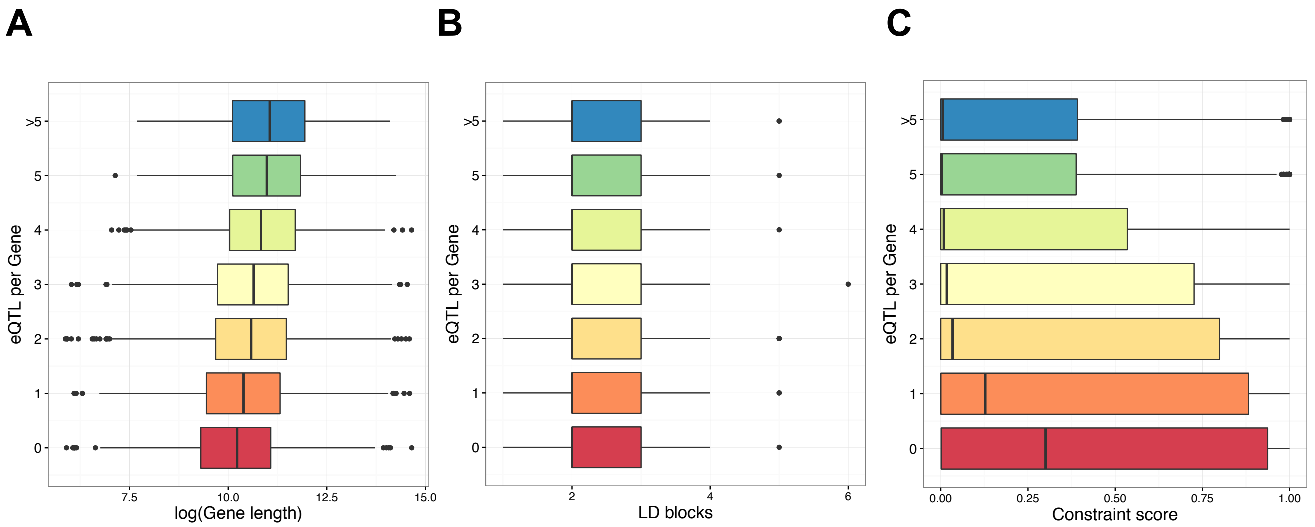

Figure S3. Correlation Between Number of Independent eQTL per Gene and Gene Length, Number of LD Blocks in the Cis- Region, and Genic Constraint

Panels (A-C) show the correlation between the number of independent eQTL per gene (0, 1, 2, 3, 4, 5, ≥6 eQTL) on the y-axis and (A) log(gene length) calculated using Ensembl gene locations, (B) number of LD blocks overlapping the cis-region (gene +/- 1Mb), and (C) genic constraint score (ExAC, based on LoF variants present) on the x-axes. There was a positive correlation between number of eQTL per gene and both log(gene length) and number of LD blocks, and there was a negative correlation between number of eQTL per gene and genic constraint score.

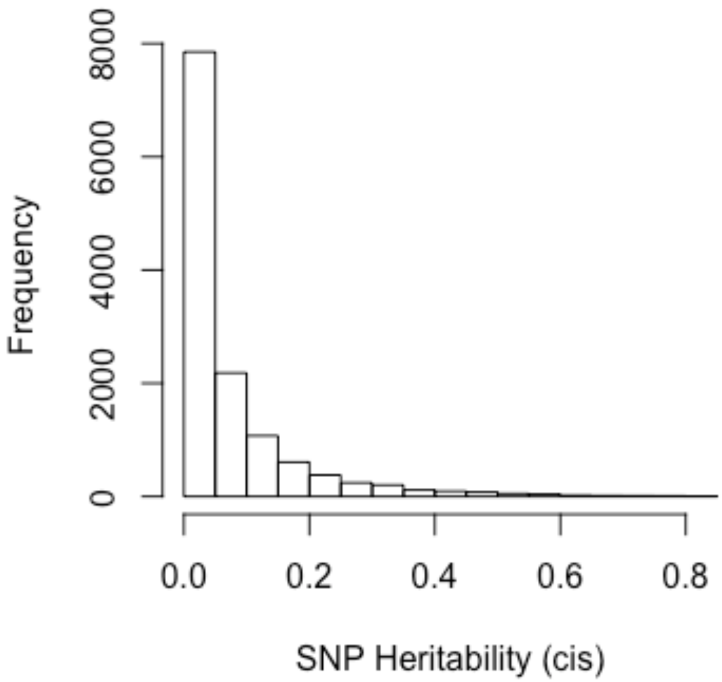

Figure S4. Distribution of Cis-SNP-Heritability per Gene

Counts of the numbers of genes (y-axis) whose variance in expression (ranging from 0 to 1, x-axis) is explained by the SNPs in its cis- region. Cis- heritability was estimated from the dosages of all imputed SNPs in the gene's cis- region (+/- 1Mb), after filtering out SNPs with MAF < 0.05 and INFO score < 0.90, using GCTA. Dosages were taken from the 467 European-ancestry individuals in the CMC cohort.

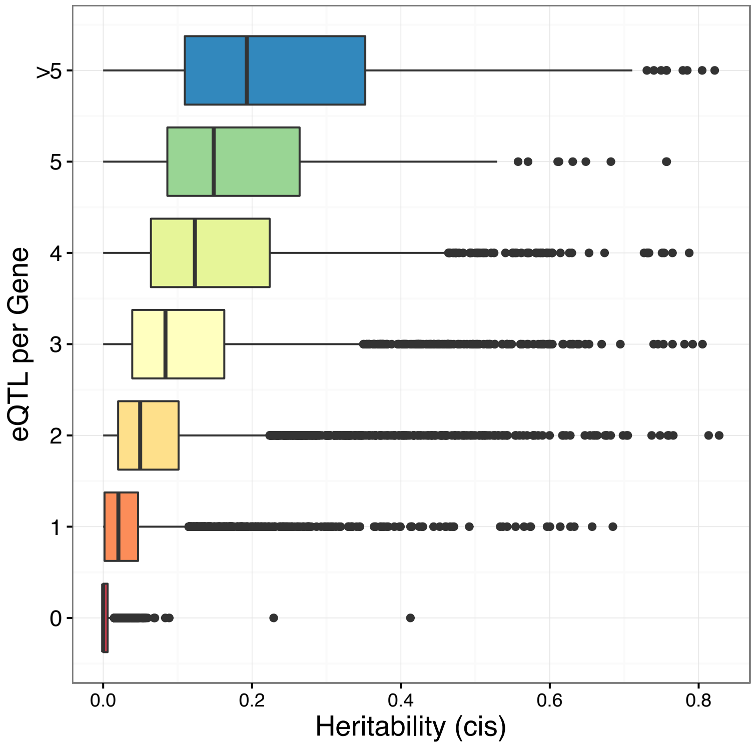

Figure S5. Correlation Between Number of Independent eQTL per Gene and Cis-Heritability

There is a positive correlation between the number of independent eQTL per gene (0, 1, 2, 3, 4, 5, ≥6 eQTL) on the y-axis and variance of expression explained by SNPs in the cis- region (cis- heritability) on the x axis.

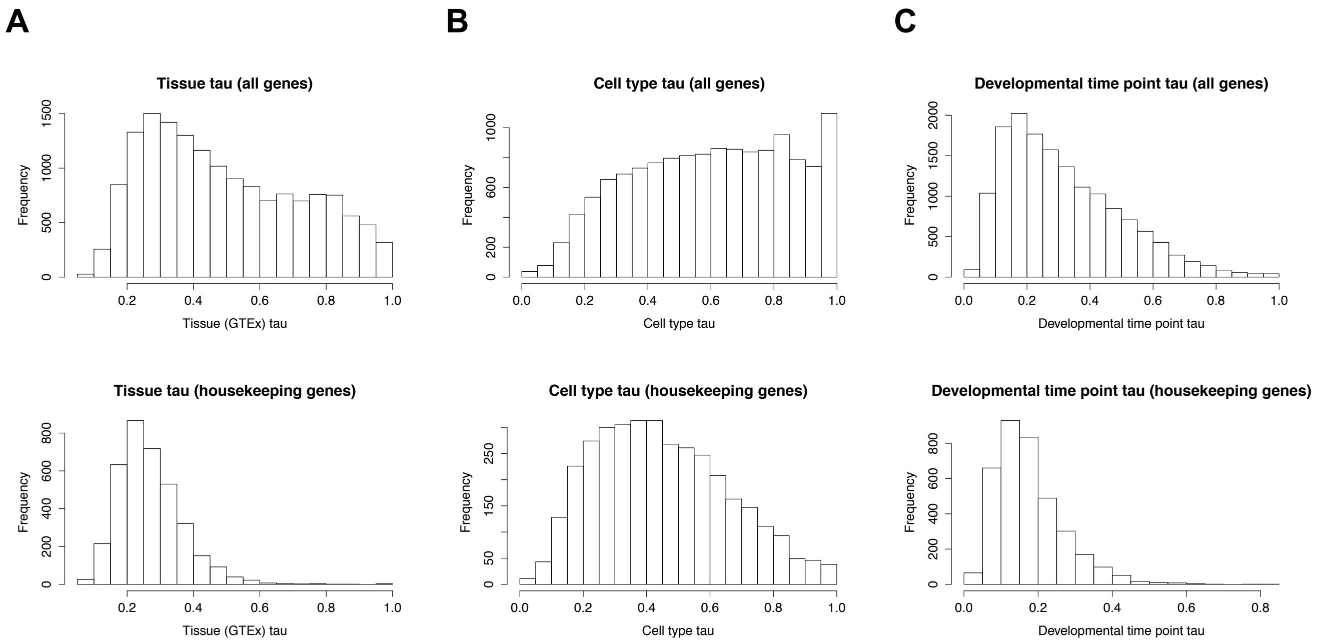

Figure S6. Distributions for Tissue, Cell Type, and Developmental Time Point Tau

Panels (A-C) show the counts of genes (y-axis) with a given tissue (A), cell type (B), or developmental time point (C) Tau (ranging from 0 to 1, x-axis), for all autosomal genes tested (top), and for a subset of housekeeping genes (bottom). Tissue Tau was calculated using expression data for 53 tissues from the GTEx project, cell type Tau was calculated using single-cell expression data for six DLPFC cell types, and developmental time point Tau was calculated using expression data for eight developmental time points from the BrainSpan atlas. For all tau measures, 0 indicates low specificity of expression, and 1 indicates high specificity of expression. The Tau distributions for housekeeping genes are all skewed right in comparison to the distributions for all genes, reflecting lower context specificity of expression for housekeeping genes.

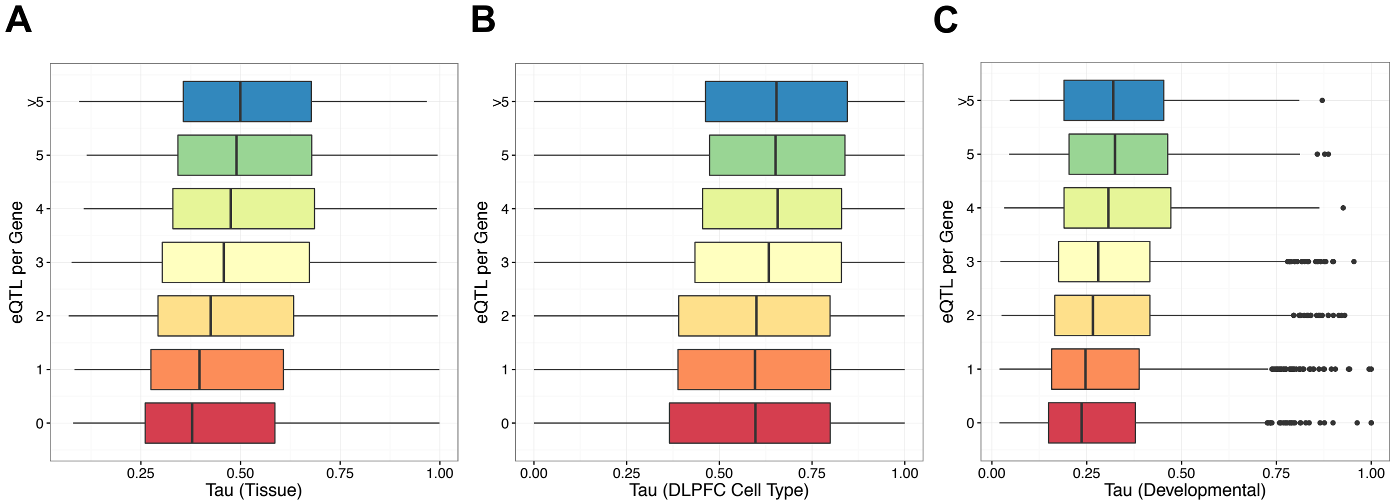

Figure S7. Correlation Between Number of Independent eQTL per Gene and Tissue, Cell Type, and Developmental Time Point Tau

Panels (A-C) show the correlation between the number of independent eQTL per gene (0, 1, 2, 3, 4, 5, ≥6 eQTL) on the y-axis and (A) tissue Tau, (B) cell type Tau, and (C) developmental time point Tau on the x-axes. There are positive correlations between the number of eQTL per gene and all three Tau measures. The strongest correlation observed is for cell type Tau.

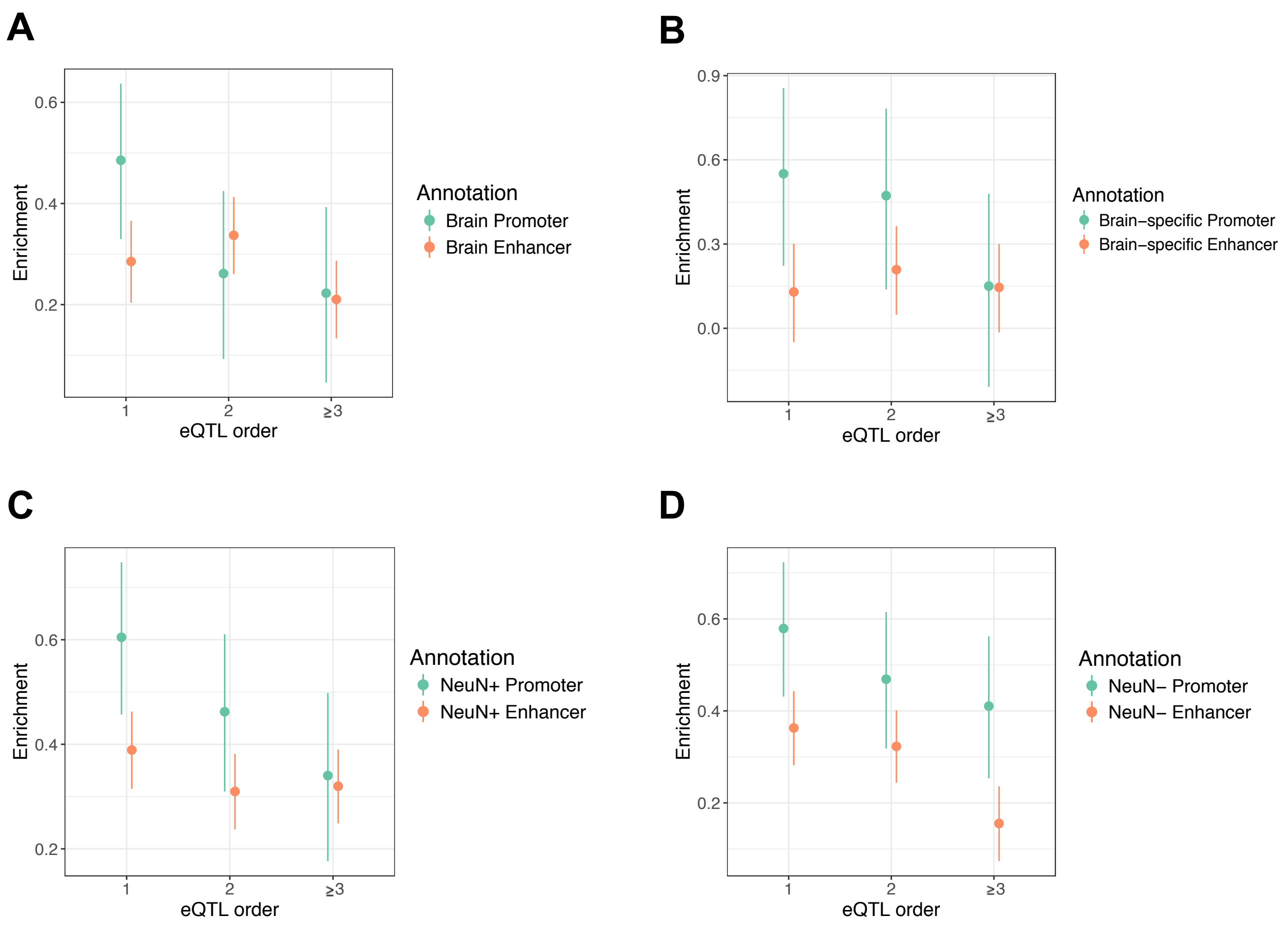

Figure S8. Enrichments of Primary and Conditional eQTL in Active Regulatory Annotations, Restricting to Match SNPs Near Brain-Expressed Genes

Plotted are enrichments (regression coefficient estimate ± 95% CI from logistic regression, y-axes) of primary (x-axis eQTL order = 1) and conditional (eQTL order = 2, ≥3) eQTL in functional annotations. Control SNPs were selected on the basis of being within 1 Mb of any of the 16,423 genes that were expressed in DLPFC and tested for eQTL. Panels (A) and (B) show enrichment in brain (union of all individual brain regions) and brain-specific (present in brain but not in seven other non-brain tissues) active promoter (turquoise) and enhancer (orange) ChromHMM states from the NIH Roadmap Epigenomics Project. Panel (C) shows enrichment in neuronal nuclei (NeuN+), for active promoters (intersection of DLPFC H3K4me3 and H3K27ac ChIP-seq peaks, turquoise) and enhancers (H3K27 peaks that do not overlap H3K4me3 peaks, orange). Panel (D) shows enrichments in the same annotations, but for DLPFC non-neuronal nuclei (NeuN-).

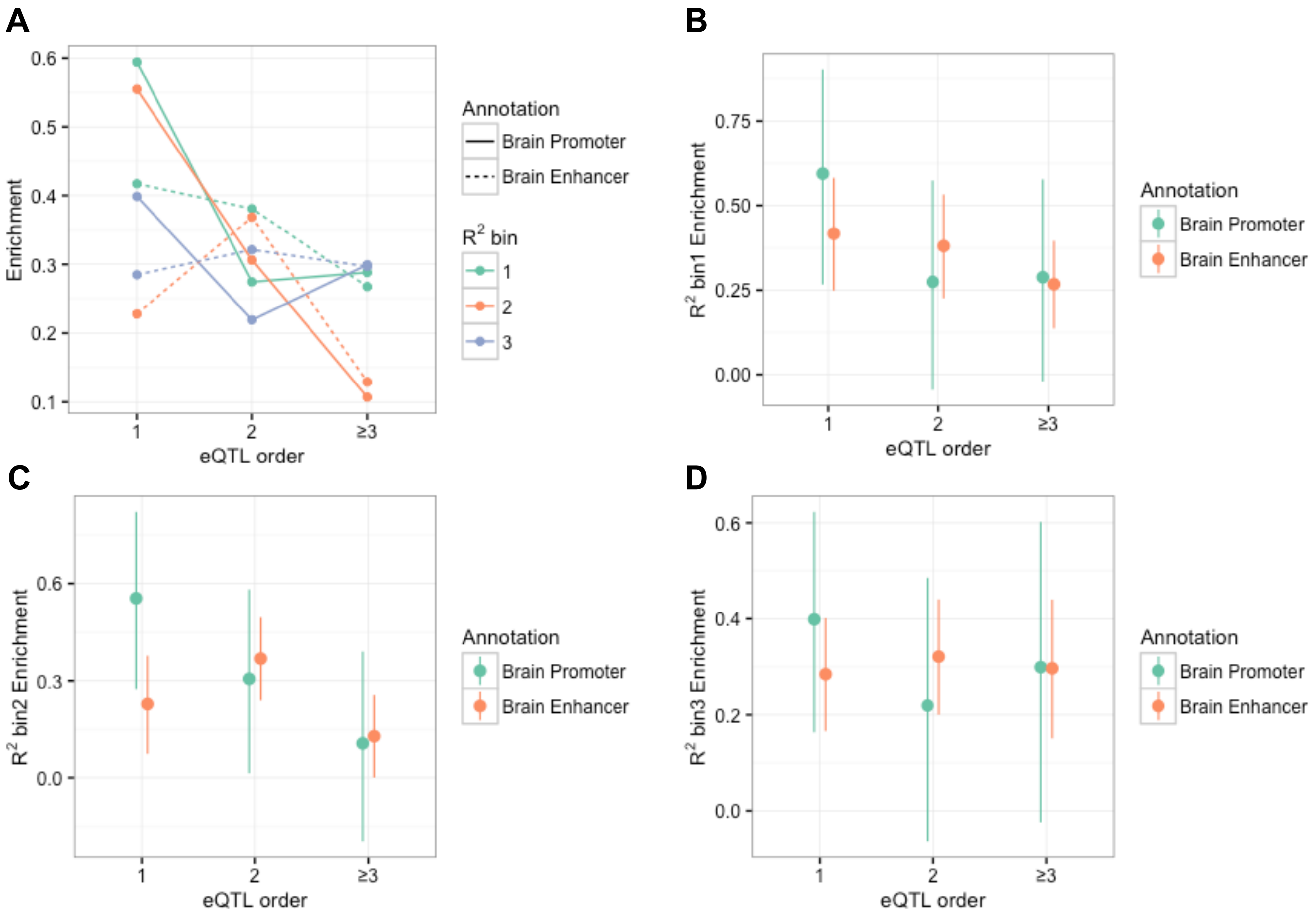

Figure S9. Variance-stratified Enrichments of Primary and Conditional eQTL in Brain Promoters and Enhancers

Plotted are enrichments (regression coefficient estimate ± 95% CI from logistic regression, y-axes) of primary (x-axis eQTL order = 1) and conditional (eQTL order = 2, ≥3) eQTL, stratified by variance in expression explained (R^2) by each eQTL, in brain promoters and enhancers. Primary and conditional eQTL were binned by R^2 value, where bin 1 consists of eQTL with the smallest R^2 estimates, and bin 3 consists of those with the highest. Panel (A) shows enrichments for all R^2 bins. Panels (B-D) show enrichments for R^2 bins 1, 2, and 3, respectively.

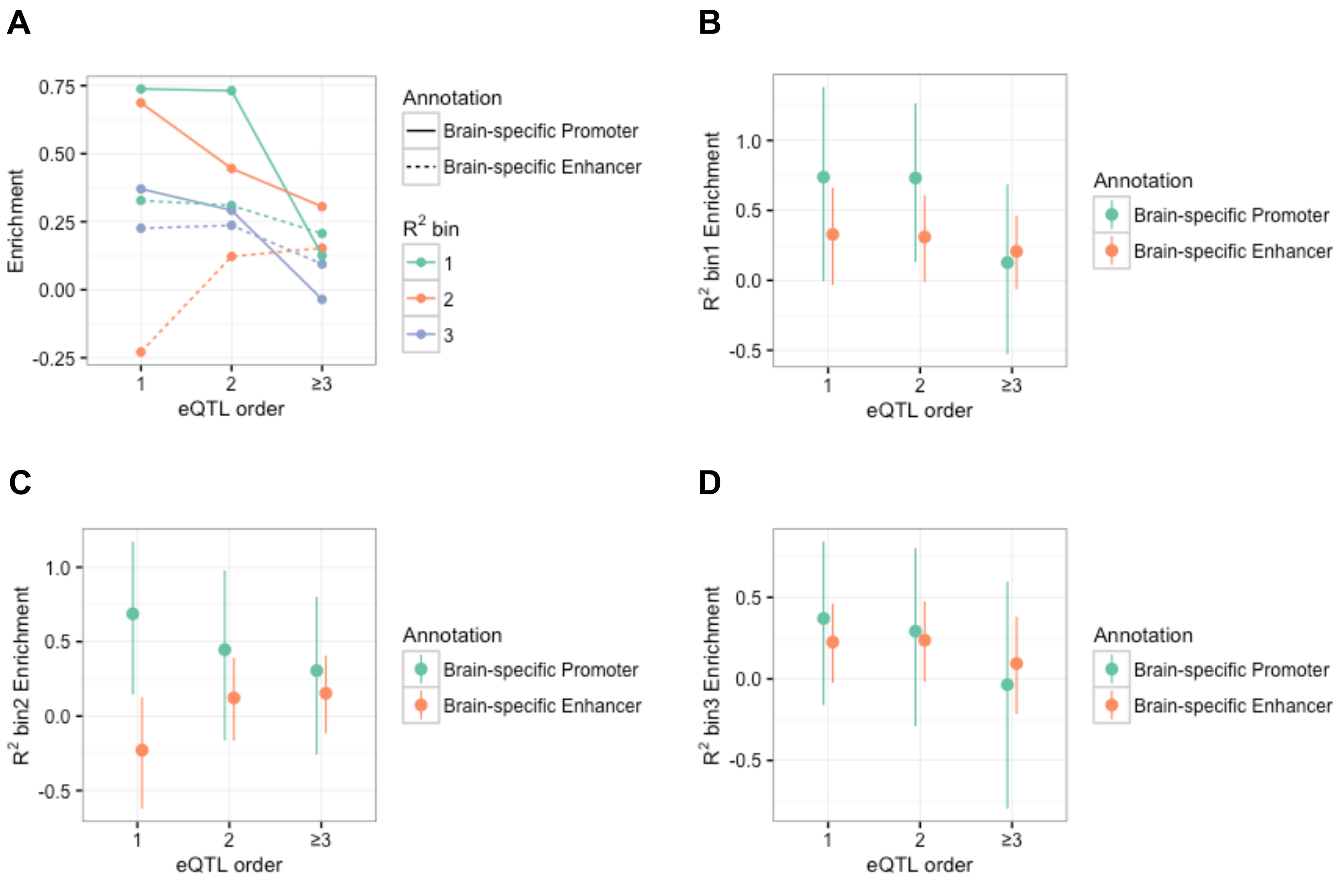

Figure S10. Variance-stratified Enrichments of Primary and Conditional eQTL in Brain-specific Promoters and Enhancers

Plotted are enrichments (regression coefficient estimate ± 95% CI from logistic regression, y-axes) of primary (x-axis eQTL order = 1) and conditional (eQTL order = 2, ≥3) eQTL, stratified by variance in expression explained (R^2) by each eQTL, in brain-specific promoters and enhancers. Primary and conditional eQTL were binned by R^2 value, where bin 1 consists of eQTL with the smallest R^2 estimates, and bin 3 consists of those with the highest. Panel (A) shows enrichments for all R^2 bins. Panels (B-D) show enrichments for R^2 bins 1, 2, and 3, respectively.

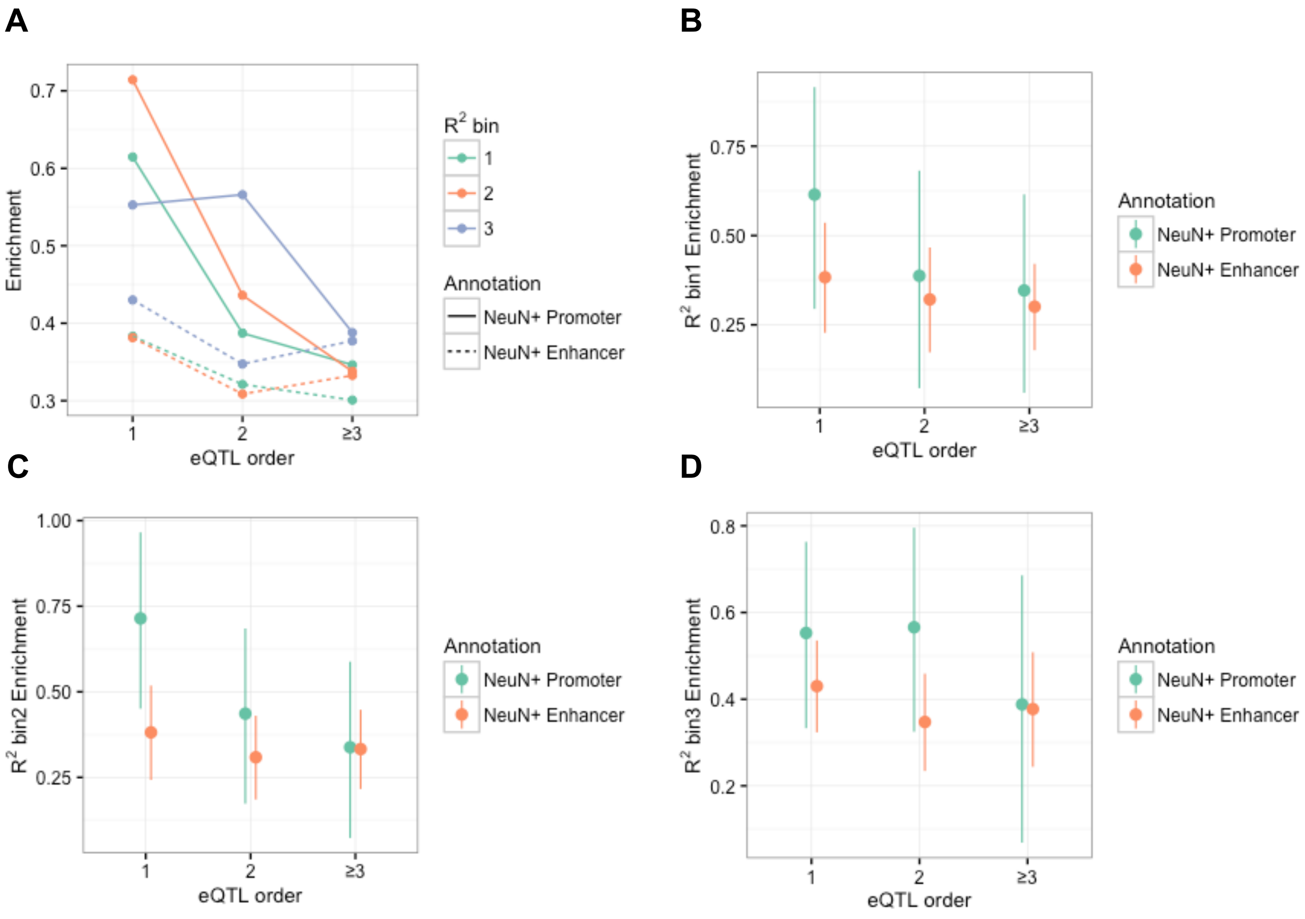

Figure S11. Variance-stratified Enrichments of Primary and Conditional eQTL in DLPFC Neuronal Promoters and Enhancers

Plotted are enrichments (regression coefficient estimate ± 95% CI from logistic regression, y-axes) of primary (x-axis eQTL order = 1) and conditional (eQTL order = 2, ≥3) eQTL, stratified by variance in expression explained (R^2) by each eQTL, in DLPFC neuronal (NeuN+) promoters and enhancers. Primary and conditional eQTL were binned by R^2 value, where bin 1 consists of eQTL with the smallest R^2 estimates, and bin 3 consists of those with the highest. Panel (A) shows enrichments for all R^2 bins. Panels (B-D) show enrichments for R^2 bins 1, 2, and 3, respectively.

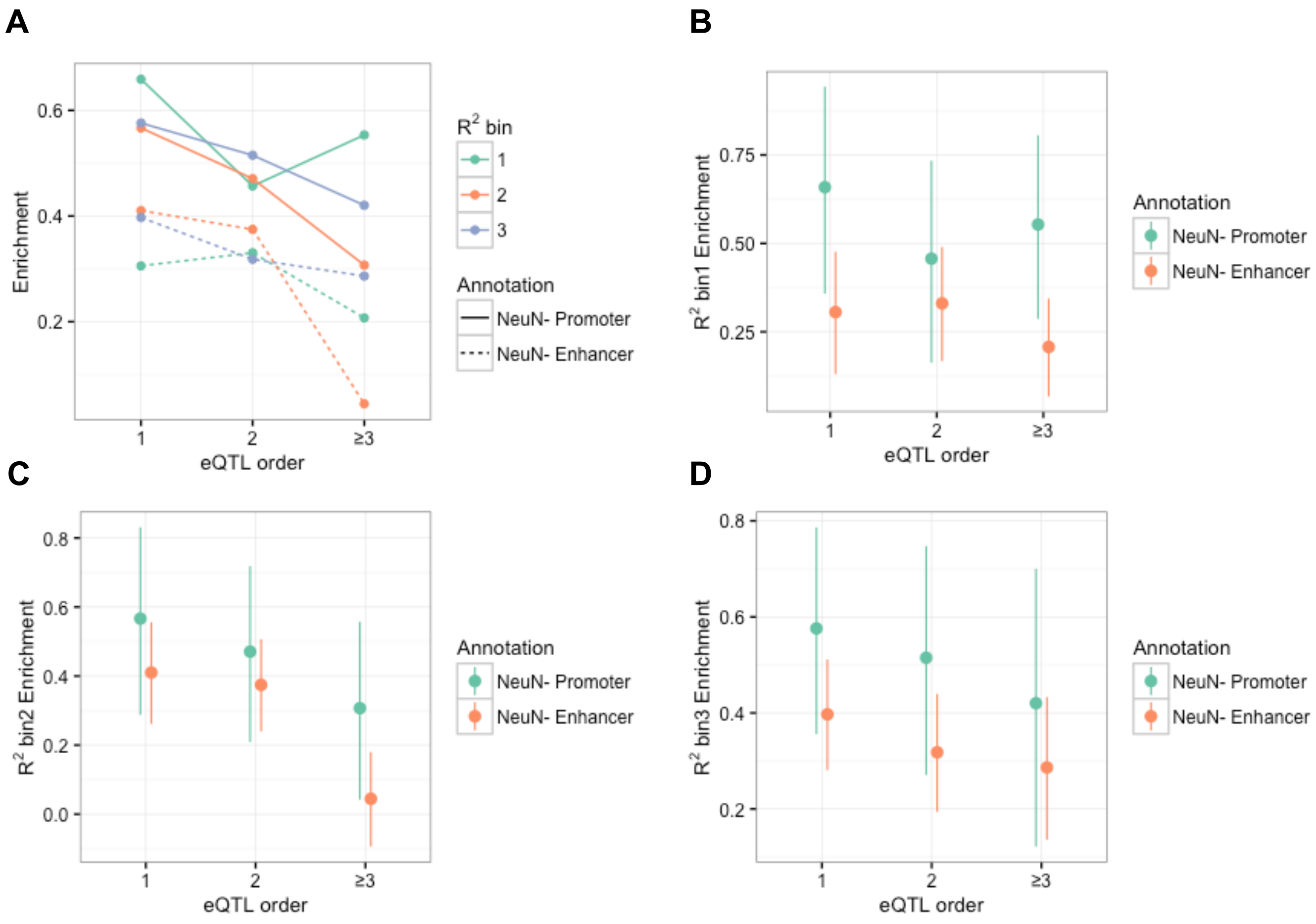

Figure S12. Variance-stratified Enrichments of Primary and Conditional eQTL in DLPFC Non-neuronal Promoters and Enhancers

Plotted are enrichments (regression coefficient estimate ± 95% CI from logistic regression, y-axes) of primary (x-axis eQTL order = 1) and conditional (eQTL order = 2, ≥3) eQTL, stratified by variance in expression explained (R^2) by each eQTL, in DLPFC non-neuronal (NeuN-) promoters and enhancers. Primary and conditional eQTL were binned by R^2 value, where bin 1 consists of eQTL with the smallest R^2 estimates, and bin 3 consists of those with the highest. Panel (A) shows enrichments for all R^2 bins. Panels (B-D) show enrichments for R^2 bins 1, 2, and 3, respectively.

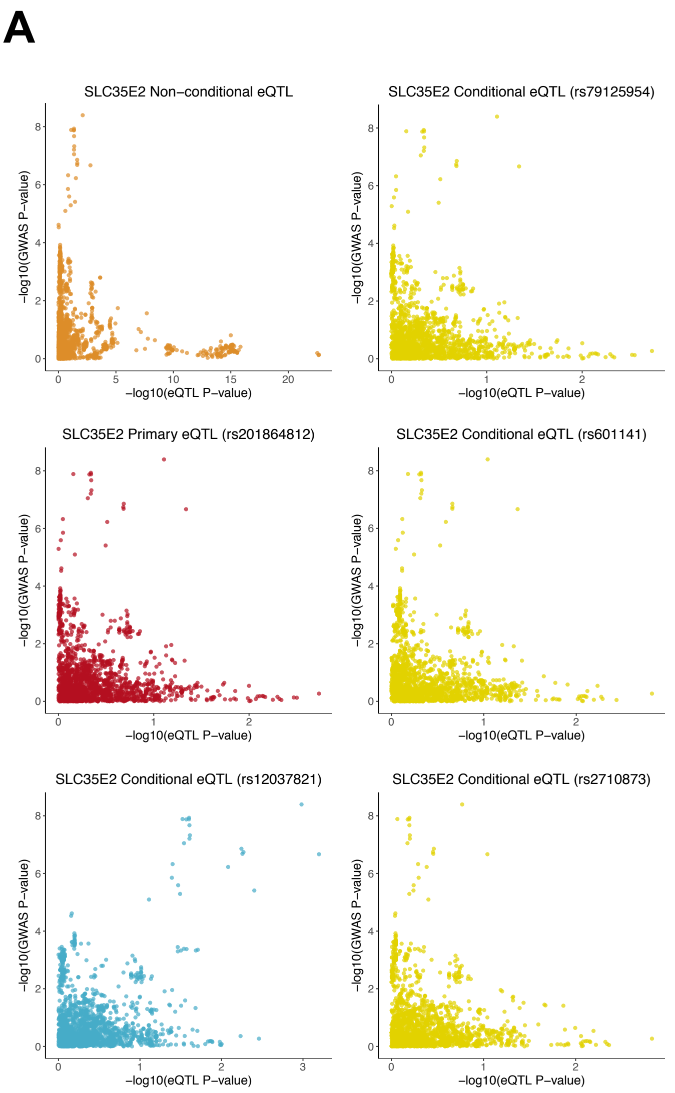

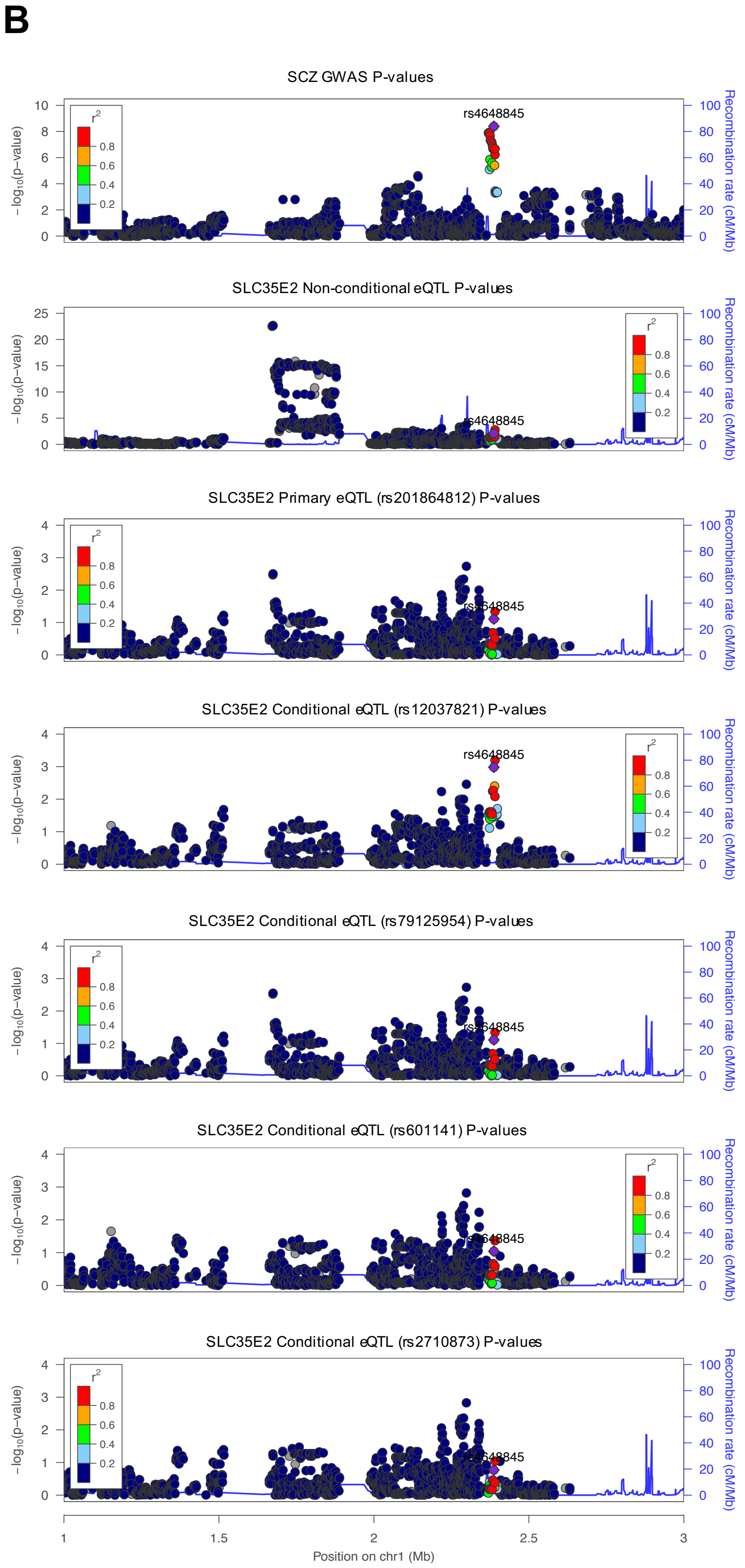

Figure S13. GWAS Signature for *SLC35E2* Co-localizes with the rs12037821 Conditional eQTL Signature

P-P plots (A) comparing GWAS P-values (y-axes) with eQTL P-values resulting from the “all-but-one” conditional analysis (x-axes) show the highest correlation to be that between the GWAS P-values and the rs12037821 conditional eQTL P-values (bottom left, blue). LocusZoom plots for the *SLC35E2* locus (B) show the GWAS signal (top) to more closely resemble the rs12037821 conditional eQTL signal (fourth from top) than the primary eQTL signal (rs201864812, third from top), non-conditional eQTL signal (second from top), or additional conditional eQTL signals. For all LocusZoom plots LD is colored with respect to the GWAS lead SNP.

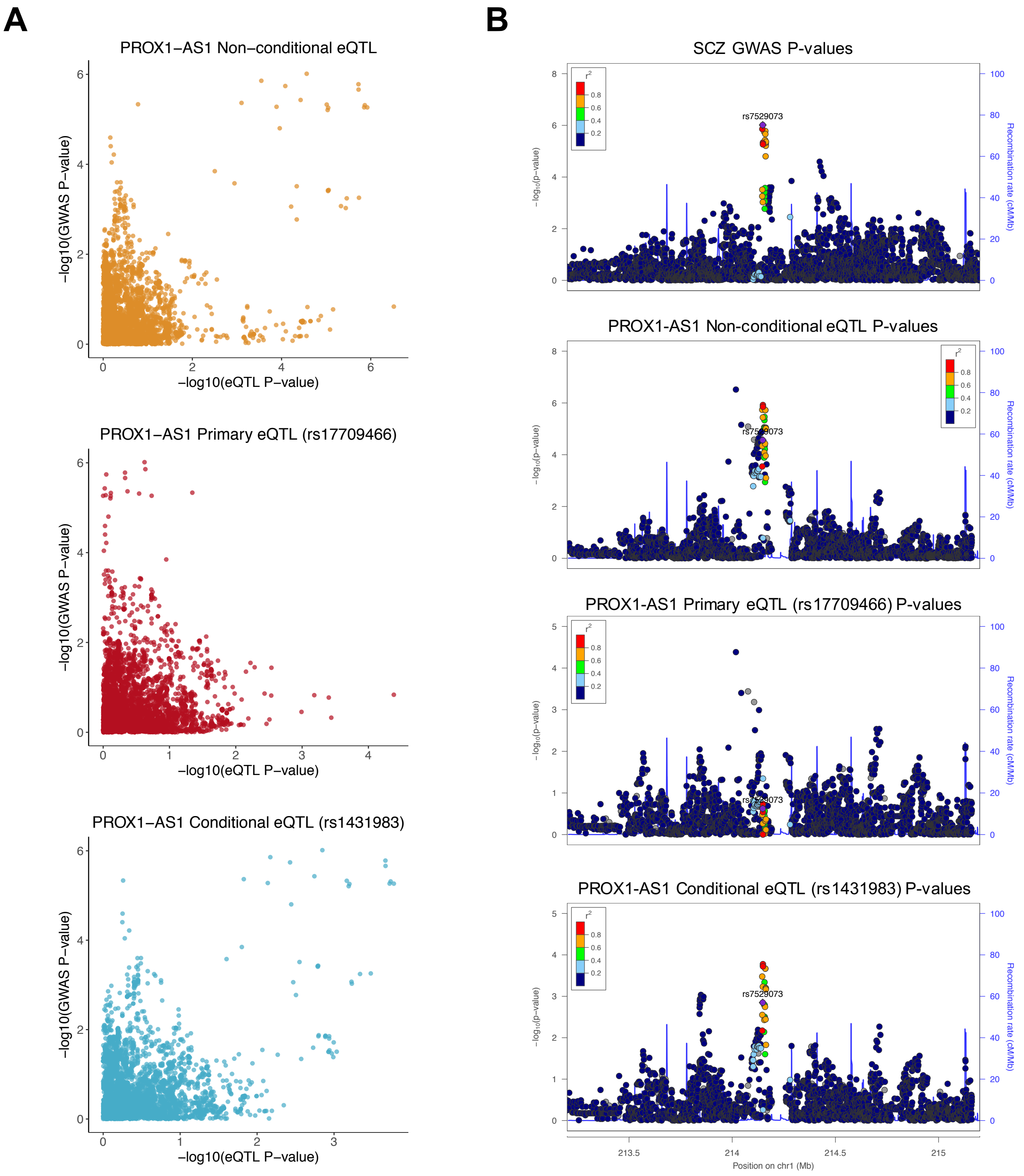

Figure S14. GWAS Signature for *PROX1-AS1* Co-localizes with the Conditional eQTL Signature

P-P plots (A) comparing GWAS P-values (y-axes) with eQTL P-values resulting from the “all-but-one” conditional analysis (x-axes) show the highest correlation to be that between the GWAS P-values and the rs1431983 conditional eQTL P-values (blue, bottom). LocusZoom plots for the *PROX1-AS1* locus (B) show the GWAS signal (top) to more closely resemble the conditional eQTL signal (rs1431983, bottom) than the primary eQTL signal (rs17709466, third from top) or non-conditional eQTL signal (second from top). For all LocusZoom plots LD is colored with respect to the GWAS lead SNP.

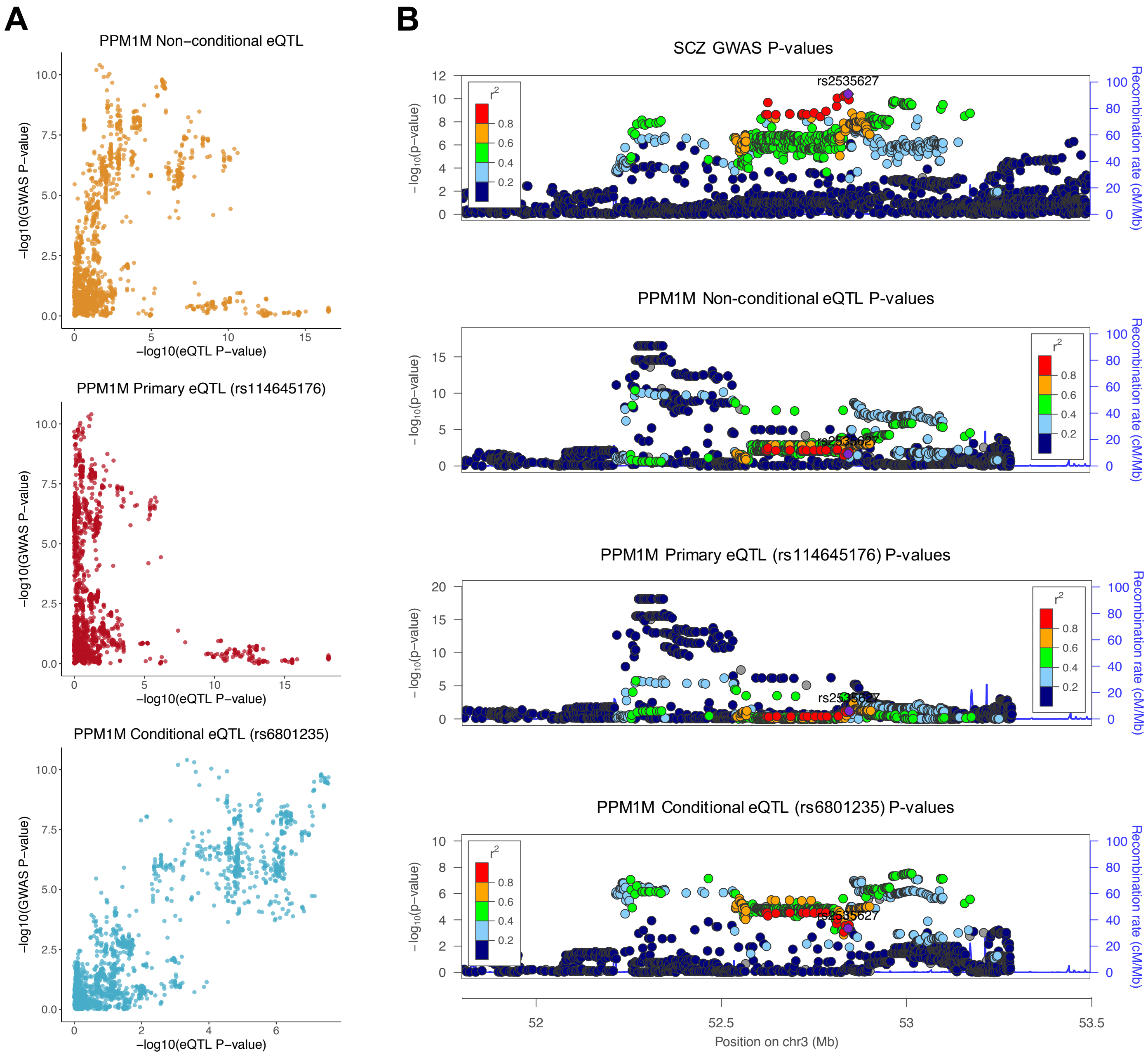

Figure S15. GWAS Signature for *PPM1M* Co-localizes with the Conditional eQTL Signature

P-P plots (A) comparing GWAS P-values (y-axes) with eQTL P-values resulting from the “all-but-one” conditional analysis (x-axes) show the highest correlation to be that between the GWAS P-values and the rs6801235 conditional eQTL P-values (blue, bottom). LocusZoom plots for the *PPM1M* locus (B) show the GWAS signal (top) to more closely resemble the conditional eQTL signal (rs6801235, bottom) than the primary eQTL signal (rs114645176, third from top) or non-conditional eQTL signal (second from top). For all LocusZoom plots LD is colored with respect to the GWAS lead SNP.

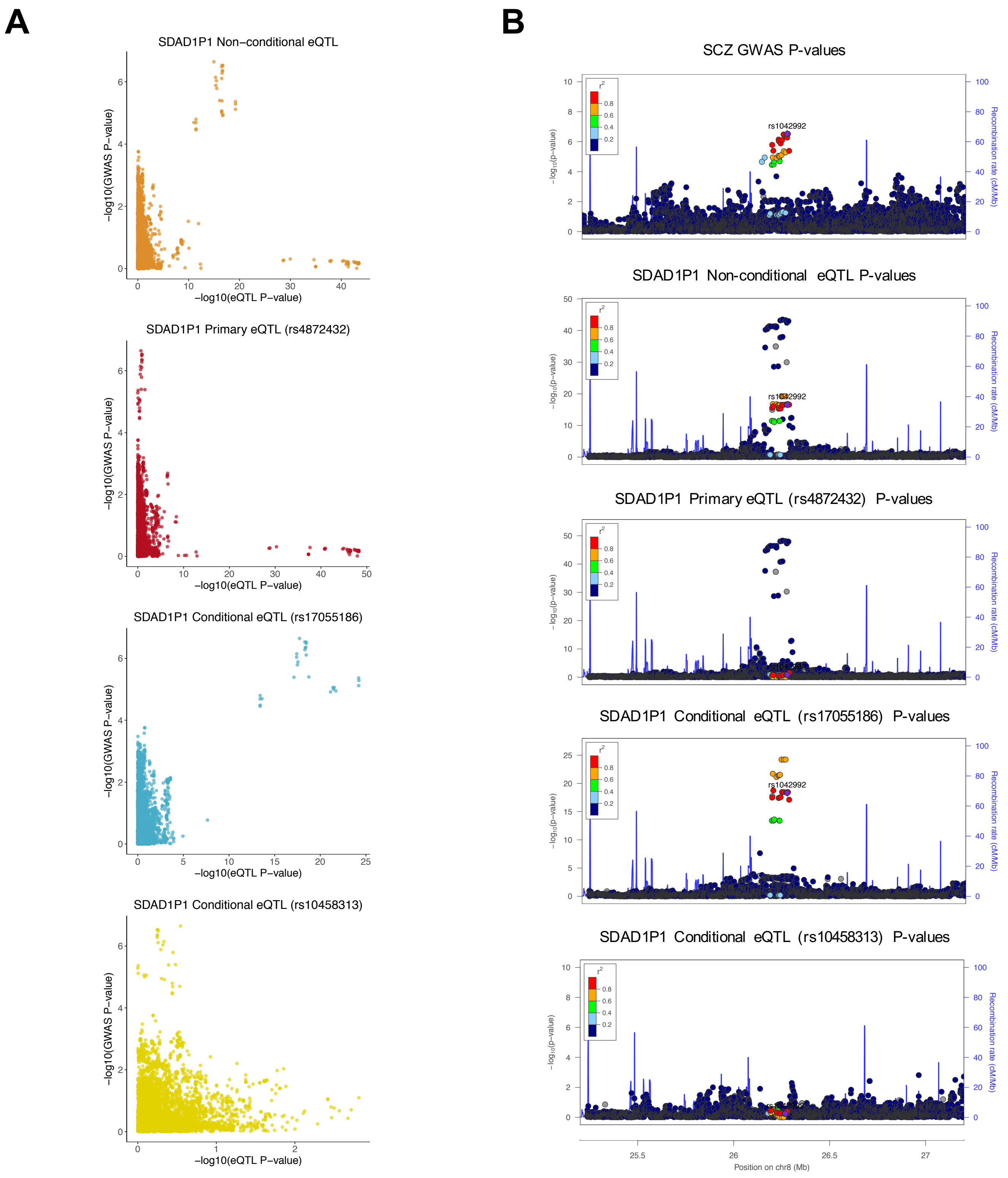

Figure S16. GWAS Signature for *SDAD1P1* Co-localizes with the Conditional eQTL Signature

P-P plots (A) comparing GWAS P-values (y-axes) with eQTL P-values resulting from the “all-but-one” conditional analysis (x-axes) shows the highest correlation to be that between the GWAS P-values and the rs17055186 conditional eQTL P-values (blue, third from top). LocusZoom plots for the *SDAD1P1* locus (B) show the GWAS signal (top) to more closely resemble the rs17055186 conditional eQTL signal (fourth from top) than the primary eQTL signal (rs4872432, third from top), non-conditional eQTL signal (second from top), or rs10458313 conditional eQTL signal. For all LocusZoom plots LD is colored with respect to the GWAS lead SNP.

A

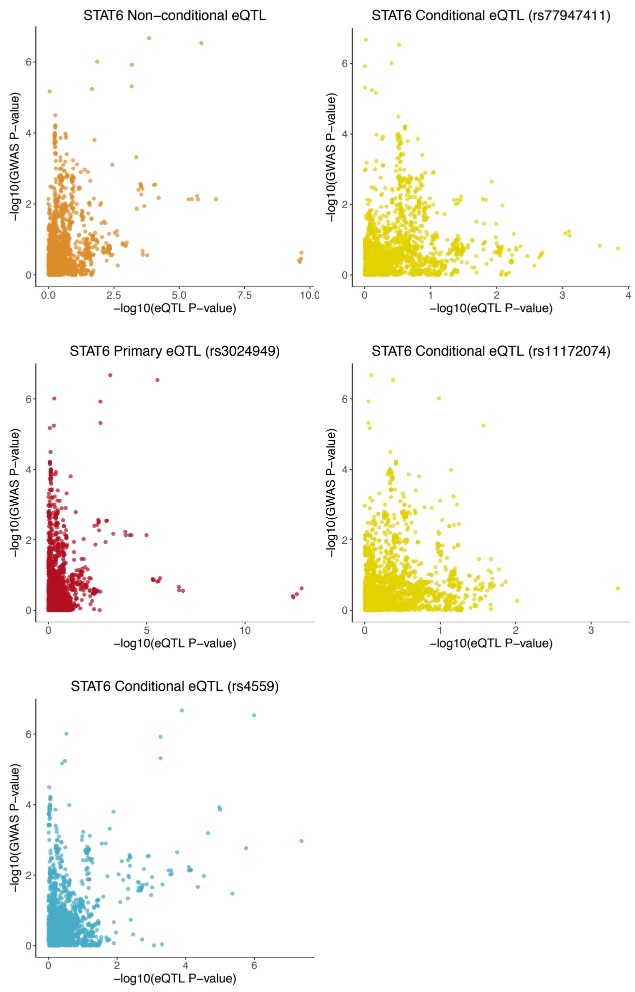

B

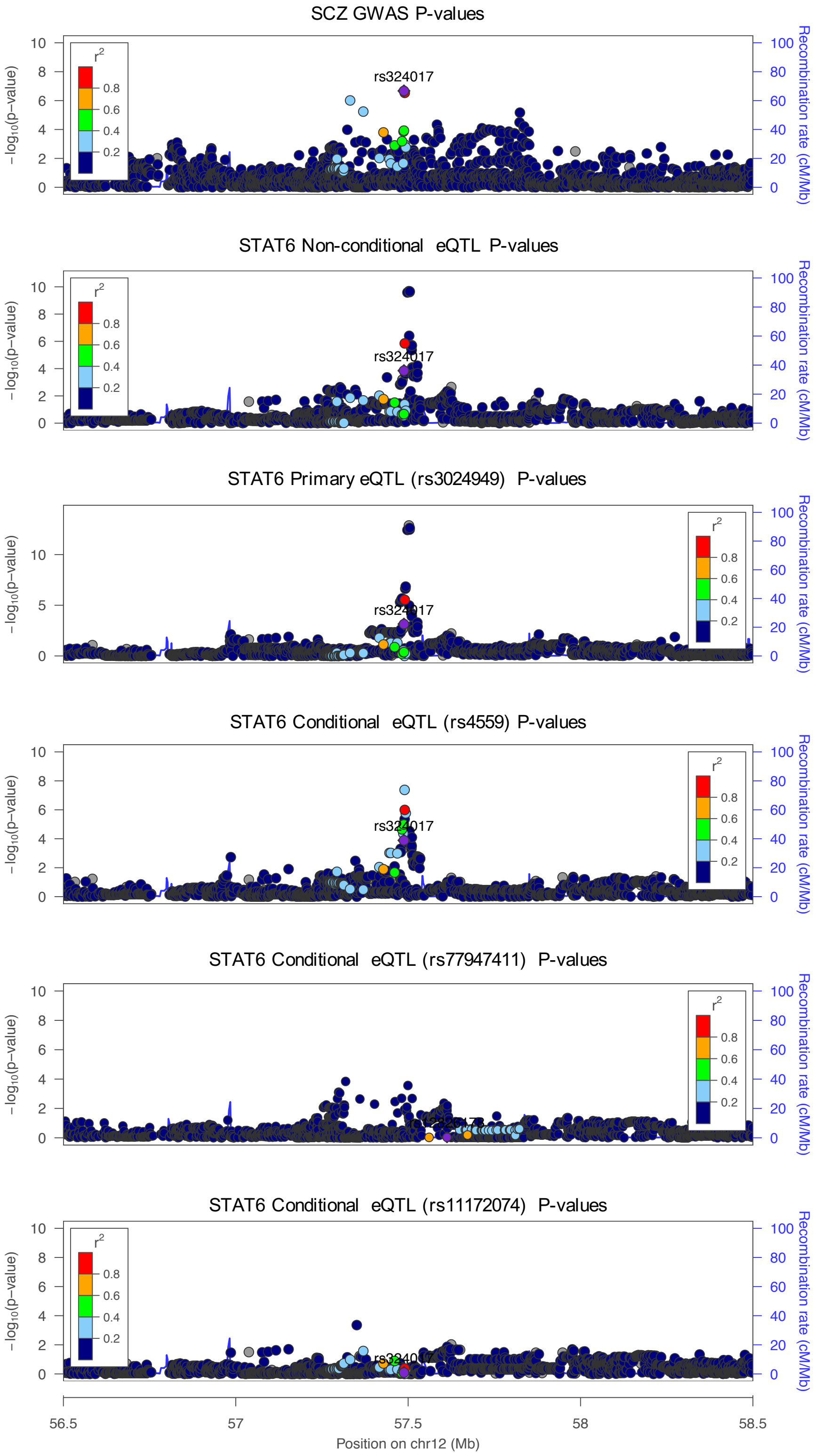

Figure S17. GWAS Signature for *STAT6* Co-localizes with the rs4559 Conditional eQTL Signature

P-P plots (A) comparing GWAS P-values (y-axes) with eQTL P-values resulting from the “all-but-one” conditional analysis (x-axes) show the highest correlation to be that between the GWAS P-values and the rs4559 conditional eQTL P-values blue, bottom left). LocusZoom plots for the *STAT6* locus (B) show the GWAS signal (top) to more closely resemble the rs4559 conditional eQTL signal (fourth from top) than the primary eQTL signal (rs3024949, third from top), non-conditional eQTL signal (second from top), or other conditional eQTL signals. For all LocusZoom plots LD is colored with respect to the GWAS lead SNP.

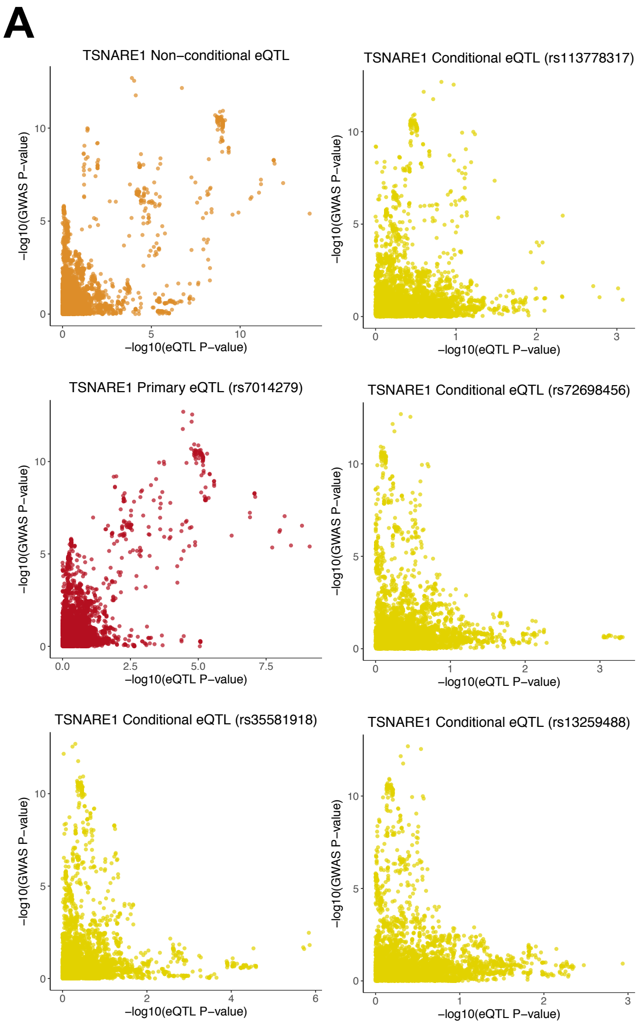

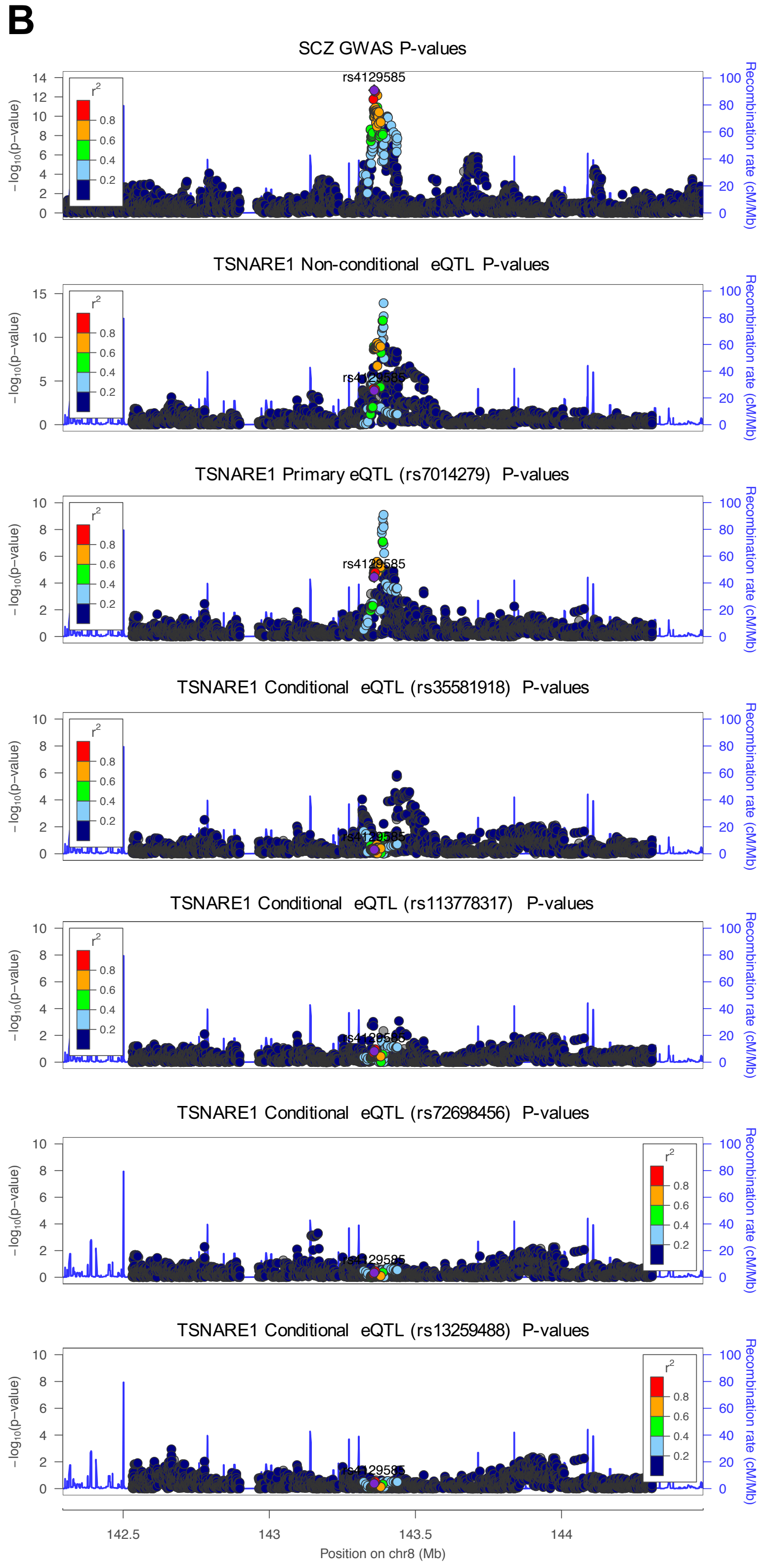

Figure S18. Lack of Co-localization Between *TSNARE1* eQTL and GWAS Signatures

P-P plots (A) comparing GWAS P-values (y-axes) with eQTL P-values resulting from the “all-but-one” conditional analysis (x-axes) suggest co-localization between the GWAS and primary eQTL (rs7014279, red) signals, but LocusZoom plots for the *TSNARE1* locus (B) show neither the non-conditional eQTL signal (second from top) nor the primary eQTL signal (third from top) to co-localize with the GWAS signal (top). For all LocusZoom plots LD is colored with respect to the GWAS lead SNP.
